# Propofol and survival: an updated meta-analysis of randomized clinical trials

**DOI:** 10.1186/s13054-023-04431-8

**Published:** 2023-04-12

**Authors:** Yuki Kotani, Alessandro Pruna, Stefano Turi, Giovanni Borghi, Todd C. Lee, Alberto Zangrillo, Giovanni Landoni, Laura Pasin

**Affiliations:** 1 Department of Anesthesia and Intensive Care, San Raffaele Hospital, IRCCS San Raffaele Scientific Institute, Via Olgettina, 60-20132 Milan, Italy; 2 School of Medicine, Vita-Salute San Raffaele University, Milan, Italy; 3 Department of Intensive Care Medicine, Kameda Medical Center, Kamogawa, Japan; 4 Division of Infectious Diseases, Department of Medicine, McGill University, Montreal, QC Canada; 5 Anesthesia and Intensive Care Unit, Padua University Hospital, Padua, Italy

**Keywords:** Systematic review, Meta-analysis, Propofol, Anesthesia, Hypnotics, Mortality, Volatile anesthetics, Organ protection

## Abstract

**Background:**

Propofol is one of the most widely used hypnotic agents in the world. Nonetheless, propofol might have detrimental effects on clinically relevant outcomes, possibly due to inhibition of other interventions' organ protective properties. We performed a systematic review and meta-analysis of randomized controlled trials to evaluate if propofol reduced survival compared to any other hypnotic agent in any clinical setting.

**Methods:**

We searched eligible studies in PubMed, Google Scholar, and the Cochrane Register of Clinical Trials. The following inclusion criteria were used: random treatment allocation and comparison between propofol and any comparator in any clinical setting. The primary outcome was mortality at the longest follow-up available. We conducted a fixed-effects meta-analysis for the risk ratio (RR). Using this RR and 95% confidence interval, we estimated the probability of any harm (RR > 1) through Bayesian statistics. We registered this systematic review and meta-analysis in PROSPERO International Prospective Register of Systematic Reviews (CRD42022323143).

**Results:**

We identified 252 randomized trials comprising 30,757 patients. Mortality was higher in the propofol group than in the comparator group (760/14,754 [5.2%] vs. 682/16,003 [4.3%]; RR = 1.10; 95% confidence interval, 1.01–1.20; p = 0.03; I^2^ = 0%; number needed to harm = 235), corresponding to a 98.4% probability of any increase in mortality. A statistically significant mortality increase in the propofol group was confirmed in subgroups of cardiac surgery, adult patients, volatile agent as comparator, large studies, and studies with low mortality in the comparator arm.

**Conclusions:**

Propofol may reduce survival in perioperative and critically ill patients. This needs careful assessment of the risk versus benefit of propofol compared to other agents while planning for large, pragmatic multicentric randomized controlled trials to provide a definitive answer.

**Graphical Abstract:**

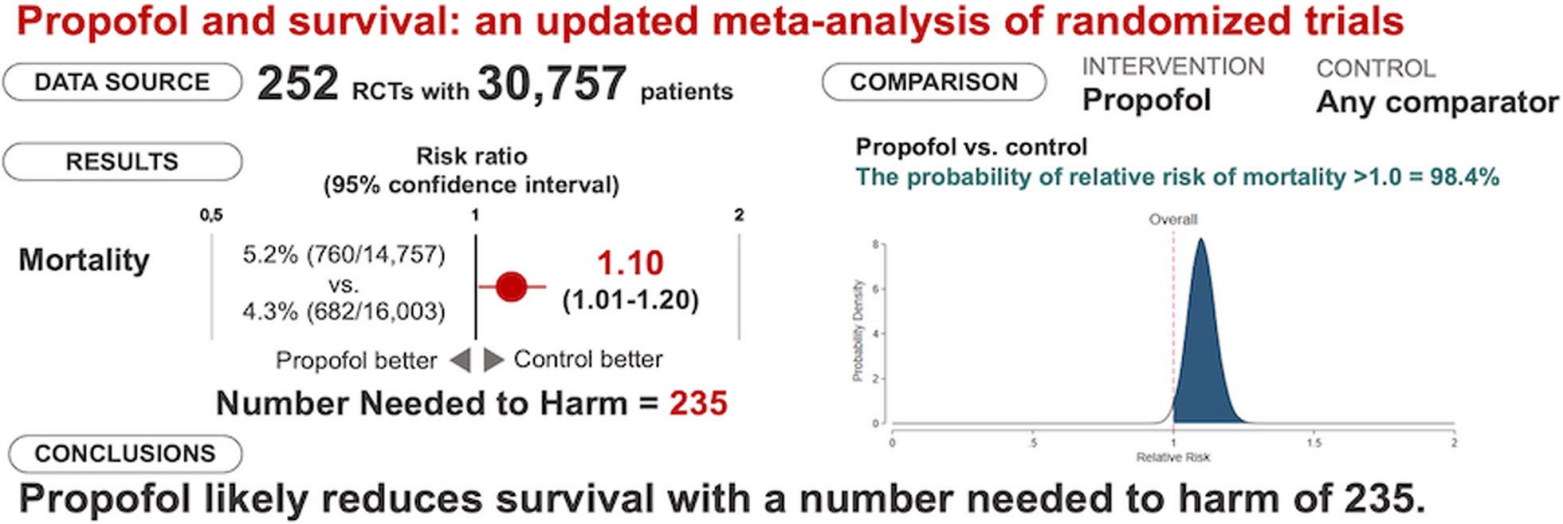

**Supplementary Information:**

The online version contains supplementary material available at 10.1186/s13054-023-04431-8.

## Background

Propofol, 2,6-diisopropylphenol, was developed in the Imperial Chemical Industries by replacing the hydrogen at the 2 position of the chemical solvent 1,3-diisopropylbenzene by a hydroxy group [1]. The unique characteristics of propofol include fast onset and rapid elimination, short duration of action, rapid recovery from anesthesia, very low incidence of adverse effects, and no mutagenic and teratogenic effects [2], all of which make propofol an ideal hypnotic agent. Considering that more than 300 million surgeries and 13–20 million intensive care unit (ICU) patients experience mechanical ventilation per year worldwide [3, 4], it is realistic to assume that hundreds of millions of patients annually receive some form of anesthesia and sedation with propofol.

On the other hand, accumulating evidence suggests the harm of propofol. Hypnotics are helpful and unavoidable in certain situations, but they also have their intrinsic adverse effects. For example, studies have shown that anesthesia depth is inversely related to the outcome of patients admitted to ICUs, irrespective of the type of hypnotics [5–7]. In addition to propofol infusion syndrome [8] and accidental microbial contamination [9–11], propofol can inhibit the organ-protective effects of different drugs and techniques, such as halogenated agents and remote ischemic preconditioning [12–14]. Furthermore, several meta-analyses showed that total intravenous anesthesia increased mortality compared with volatile anesthetic agents in cardiac surgery populations [15, 16]. Although a randomized trial found an increase in the mortality rate among children receiving sedation with propofol in pediatric intensive care units [17], leading to safety warnings from the Food and Drug Administration [18], there are numerous reports on the use of propofol for long-term sedation in critically ill children [19–21]. We performed a meta-analysis of randomized controlled trials (RCTs) on propofol versus any comparator in postoperative and critically ill patients to test the hypothesis that propofol could increase mortality.

## Methods

We registered the present meta-analysis in the PROSPERO International prospective register of systematic reviews (CRD42022323143). We used the following PICO (Population, Intervention, Comparison, Outcome, Study design) criteria: patients receiving general anesthesia or sedation (P); propofol (I); any comparator drug (C); mortality at the longest follow-up available (O); randomized clinical trials (S). We followed the Preferred Reporting Items for Systematic Reviews and Meta-Analyses (PRISMA) guidelines (see Additional file 1: Table S1 for completed checklist) [22].

### Search strategy and selection criteria

Four researchers independently searched PubMed, Google Scholar, the Cochrane Central Register of Controlled Trials, and ClinicalTrials.gov to identify relevant studies from inception to August 19, 2022. We also searched for abstracts of major congresses within these three years to minimize the risk of missing eligible but not yet published studies. The complete PubMed search strategy we used to identify studies of interest is presented in Additional file 1. All RCTs comparing propofol versus any comparator in any clinical setting were considered eligible. Exclusion criteria were randomized trials with a non-parallel design (i.e., cross-over), overlapping publications, non-human experimental studies, studies where the comparator was loco-regional anesthesia, studies where propofol was used for palliative or end-of-life care, or studies where propofol was used as a single bolus in the intervention arm or during different minor procedures (e.g., gastrointestinal endoscopies, bronchoscopies, painful procedures requiring sedation). No restriction was applied to patient age or language. After removing duplicates, two investigators assessed eligibility at the title/abstract level and downloaded the full text of all potentially relevant articles. In case of disagreement, the final assessment was discussed with two other senior investigators.

### Data collection and risk of bias assessment

Data of interest were extracted by two investigators and collected in a standardized data collection form. The following variables were collected: PubMed unique Identifier, journal, first author, year of publication, name of the corresponding author, number of patients who received propofol, number of patients who received the comparator, type of control drug, number of deaths in each group, settings (ICU, non-cardiac surgery, cardiac surgery, adult or pediatric population). The primary outcome was all-cause mortality at the longest follow-up available. After extracting data from the manuscripts, we realized that a minimal percentage (less than 10%) of included studies reported 30-day mortality and that available literature supports that time points do not influence pooled point estimates of the effects on mortality in critical care randomized trials [23]. We therefore decided to shift from the originally planned 30-day mortality to the more consistent longest follow-up available mortality as the primary outcome.

The risk of bias of each included RCT was assessed using the Cochrane risk-of-bias tool for randomized trials version 2 (RoB 2) [24]. The publication bias was evaluated by visual inspection of the funnel plot.

### Data analysis and synthesis

Computations were performed using IBM SPSS Statistics for Macintosh, Version 28.0. We planned to use a fixed-effects Mantel–Haenszel model on the Relative Risk (RR) scale if heterogeneity was low (≤ 25%) and a random-effects Mantel–Haenszel model if heterogeneity was high (> 25%). Heterogeneity was quantified using I squared (I^2^) and Tau 2.

The following formula was used to calculate the number needed to harm (NNH) for mortality: NNH = 1/(the weighted risk ratio of the propofol group—the absolute risk of the control group). We reported NNH as positive whole numbers with all decimals rounded up. Unadjusted p values were reported throughout the manuscript. Statistical significance was set at the two-tailed 0.05 level for hypothesis testing. The following subgroup analyses pre-specified in the PROSPERO were performed: different clinical settings (cardiac surgery, non-cardiac surgery, or ICU), surgical or non-surgical, and use of propofol bolus infusion in the comparator arm. Although not pre-registered in the PROSPERO, the following sensitivity analyses were also conducted to assess the robustness of the main analysis: pediatric or adult population, different comparators (volatile, intravenous, or miscellaneous), large (randomizing ≥ 500 patients) or small (< 500 patients) studies, studies with higher (> 4.5%) or lower (≤ 4.5%) mortality, exclusion of high risk of bias studies, studies published after 2005, using the Peto method, using a random-effects model, studies reporting hospital or long-term mortality (i.e., mortality at ≥ 30 days after randomization), and different timepoints of mortality (hospital, periprocedural, intensive care unit, 30 days, and 1 year).

To contextualize and visualize the main findings, the relative risk (RR) and 95% CIs for mortality were used, simulating 100,000 trials on the log scale and generating a representative probability density function on the risk ratio scale using kernel density estimation, and estimating the probability of any harm (RR ≥ 1) using STATA v.17 (STATACorp, College Station, USA). This analysis was repeated for the cardiac surgery, non-cardiac surgery, and ICU subgroups, respectively. As a post hoc sensitivity analysis, we also conducted a Bayesian meta-analysis using R [25] and the MetaStan package [26] with a binomial-normal hierarchical model and weakly informative priors [mu N ~ (0, 10); theta N ~ (0, 2.82); tau half-normal (0.5)] [27]. This method provides results on the log-odds ratio scale which were exponentiated to estimate the posterior probability of harm (odds ratio > 1). Unlike the Mantel–Haenszel method, this method incorporates trials with zero events in both arms without requiring continuity corrections.

A trial sequential analysis (TSA) was also conducted for mortality with a diversity-adjusted information size calculated using a two-sided alpha of 0.05 and a power of 80%. We assumed a relative risk increase of 10% and derived the control event proportion from included studies. We used the TSA Viewer software (Version 0.9 0.5 0.10 Beta. Copenhagen Trial Unit, Centre for Clinical Intervention Research, Rigshospitalet, Copenhagen, Denmark).

## Results

**Fig. 1:**
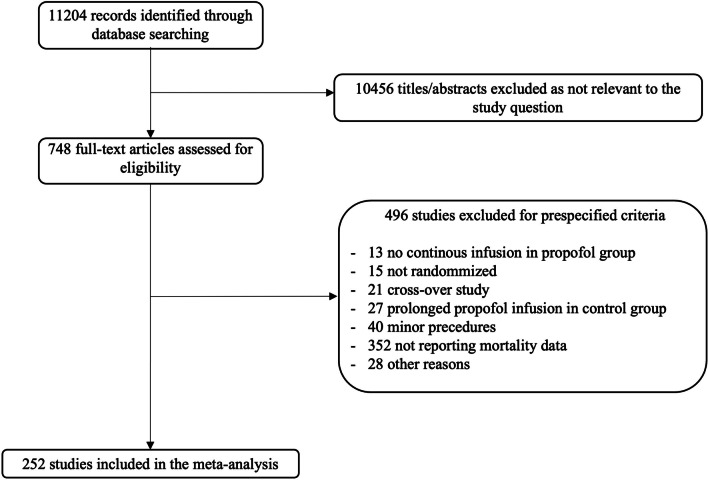
Flow chart of study selection

From 11,204 records identified through literature search, we included 252 RCTs with a total of 30,757 patients (Fig. 1). Studies were published between 1987 and 2022, and mostly enrolled adult patients (235 studies). The most common setting was non-cardiac surgery (153 studies), followed by intensive care (52 studies) and cardiac surgery (47 studies). Volatile anesthetics were used as a comparator in 172 studies, while intravenous agents were assessed in 71 studies. Hospital mortality was the most frequently reported timepoint of mortality reported in the included studies (69 studies (27%), Additional file 1: Table S2 and Table S3). The complete reference list of included studies is available in Additional file 2.

**Fig. 2:**
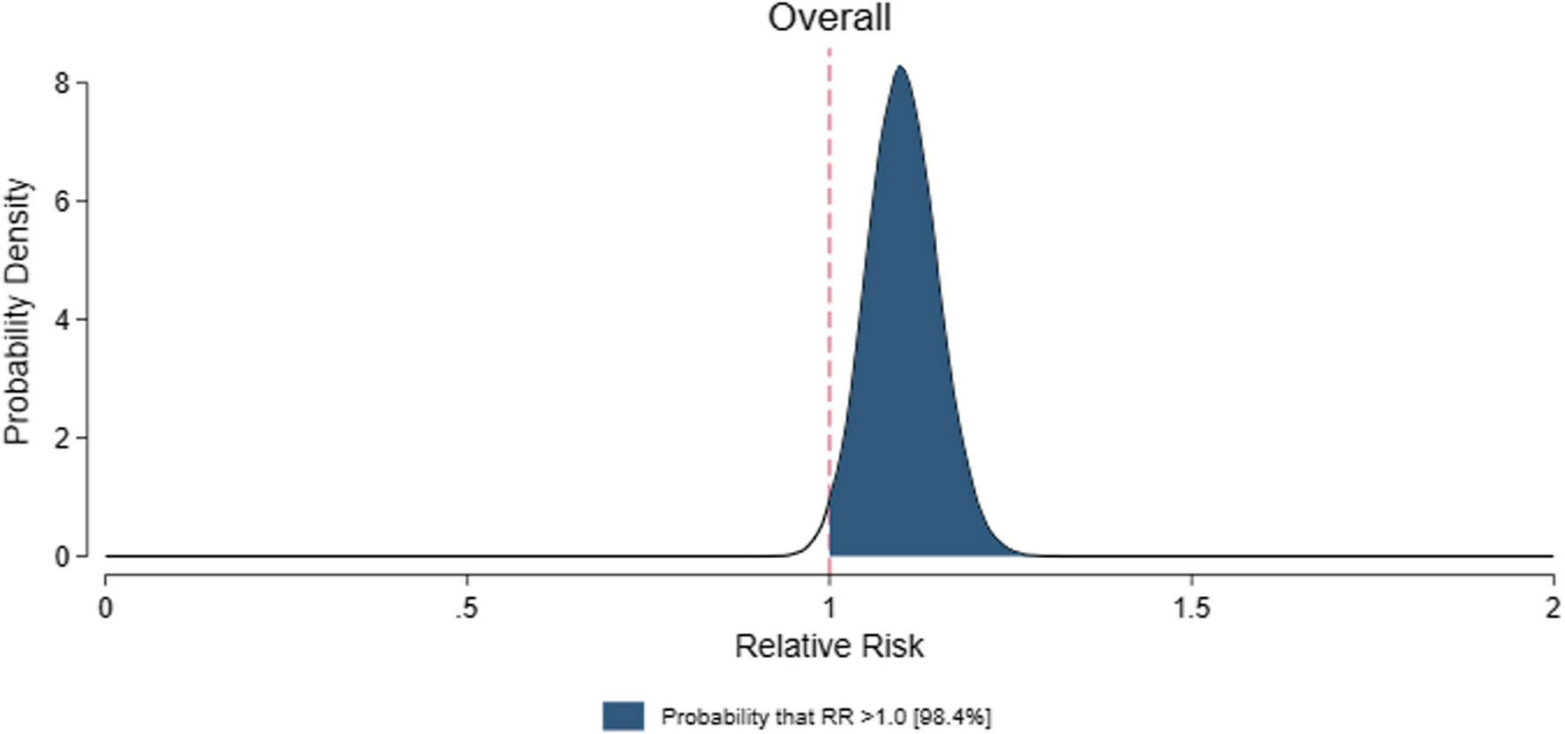
Probability density function for combined posterior distribution of the difference in mortality in the overall population

**Fig. 3:**
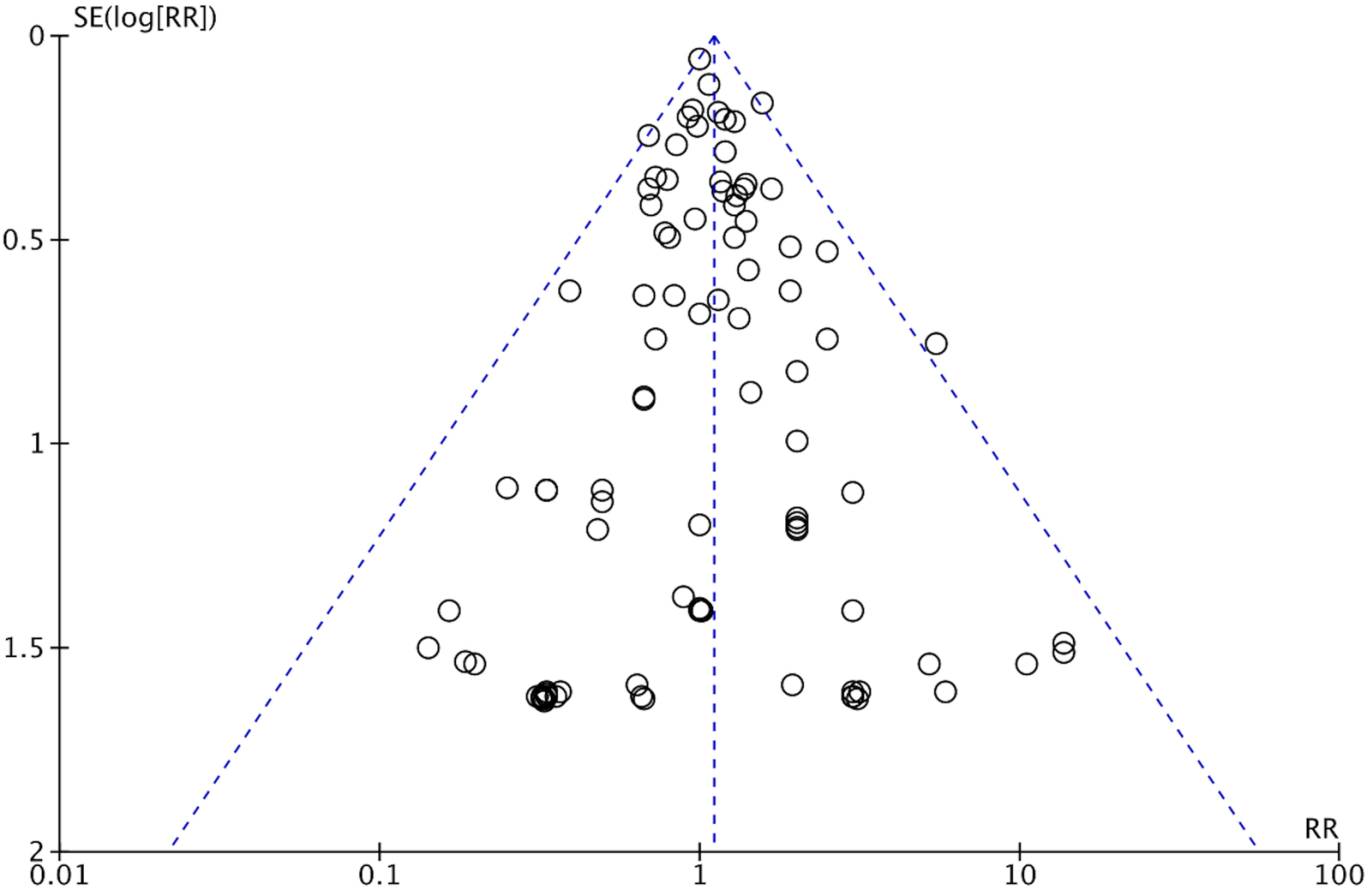
Funnel plot for mortality in the overall population

Mortality was higher in the propofol group than in the comparator group (760/14,754 [5.2%] vs. 682/16,003 [4.3%]; RR = 1.10; 95% CI, 1.01–1.20; p = 0.03; I^2^ = 0%; NNH = 235) (Table 1 and Additional file 1: Fig. S1). A Bayesian approach showed that the probability of any increase in mortality was 98.4% (Fig. 2). Funnel plots found no major asymmetry (Fig. 3). Bubble plots showed no relationship between the year of publication and the effect of propofol on mortality (Additional file 1: Fig. S2).

**Table 1:** Effect of propofol on mortality in the overall population and subgroups

	No. of studies	Propofol	Control	Risk ratio (95% CI)	P value	I^2^ (%)
Overall population	252	760/14,754 (5.2%)	682/16,003 (4.3%)	1.10 (1.01–1.20)	0.03	0
Subgroup analyses						
Cardiac surgery vs. non-cardiac surgery vs. intensive care unit						
Surgical	200	294/11,617 (2.5%)	241/12,650 (1.9%)	1.21 (1.04–1.41)	0.01	0
Cardiac surgery	47	118/2,591 (4.6%)	83/2,927 (2.8%)	1.46 (1.13–1.89)	0.004	0
Non-cardiac surgery	153	176/9,026 (1.9%)	158/9,723 (1.6%)	1.09 (0.90–1.31)	0.39	0
Intensive care unit	52	466/3,137 (15%)	441/3,353 (13%)	1.04 (0.93–1.16)	0.50	0
Adult vs. pediatric^§^						
Adult	235	690/14,044 (4.9%)	628/15,312 (4.1%)	1.10 (1.00–1.21)	0.04	0
Pediatric	17	70/710 (9.9%)	54/691 (7.8%)	1.12 (0.83–1.52)	0.46	24
Comparator: volatile anesthesia vs. total intravenous anesthesia vs. miscellaneous^§^						
Volatile anesthesia	172	251/9,186 (2.7%)	197/9,764 (2.0%)	1.25 (1.06–1.47)	0.009	11
Total intravenous anesthesia	71	438/4,900 (8.9%)	420/5,161 (8.1%)	1.03 (0.92–1.15)	0.65	0
Miscellanea	9	71/668 (11%)	65/1,078 (6.0%)	1.13 (0.86–1.48)	0.37	31
Bolus use of propofol in the comparator arm*						
Yes	75	146/4,076 (3.6%)	105/4,635 (2.3%)	1.33 (0.82–2.16)	0.26	64
No	166	586/9,376 (6.3%)	567/10,567 (5.4%)	1.03 (0.94–1.14)	0.50	0
Large vs. small^§^						
Large (≥ 500 patients)	6	107/2,918 (3.7%)	74/2,915 (2.5%)	1.45 (1.10–1.92)	0.009	0
Small (< 500 patients)	246	653/11,836 (5.5%)	608/13,088 (4.6%)	1.06 (0.97–1.16)	0.23	0
High vs. low mortality in the comparator arm^§^						
High (≥ 4.5%)	51	646/3,193 (20%)	599/3,214 (19%)	1.06 (0.97–1.17)	0.20	0
Low (< 4.5%)	201	114/11,561 (1.0%)	682/16,003 (0.6%)	1.35 (1.03–1.76)	0.03	0
Exclusion of high risk of bias studies^**§**^	211	662/12,990 (5.1%)	586/14,057 (4.2%)	1.12 (1.02–1.23)	0.02	0

*CI* confidence interval, *N/A* not applicable

^§^These sensitivity analyses were not pre-specified in the PROSPERO registration

^*^No information was available in 11 studies

A statistically significant mortality increase in the propofol group was also found in the subgroups of cardiac surgery, surgical settings, adults, volatile agent as a comparator, studies enrolling ≥ 500 patients, and studies with low mortality in the comparator arm. The other subgroup and sensitivity analyses observed a treatment effect toward harm of propofol without significant differences (Table 1). In particular, the probabilities of harm in the cardiac, non-cardiac surgery, and ICU groups were 99.8%, 81.6%, and 75.7%, respectively, confirmed by a sensitivity analysis with a binomial model. The results of the subgroup and sensitivity analyses and Bayesian figures are in Additional file 1: Fig. S3-29 and Table S4.

TSA was inconclusive, as the Z-curve did not cross either O'Brien-Fleming alpha-spending boundary (Additional file 1: Fig. S30).

According to the RoB 2 assessment, 97 (38%) were judged at low risk of bias, 114 (45%) had some concerns, and 41 (16%) were at high risk of bias (Additional file 1: Table S2).

## Discussion

### Key findings

This meta-analysis of 252 randomized trials found that propofol significantly increases mortality compared with other hypnotic agents. Of note, the magnitude and direction of the treatment effect were maintained in all analyzed subgroups. The harm of propofol was remarkable in the cardiac surgical setting and in those studies with volatile agents as comparator.

### Relationship with previous literature

Propofol is a short-acting hypnotic agent with reasonable costs and acceptable hemodynamic impact. There are 300 million patients undergoing surgeries and 13–20 million ICU patients experiencing mechanical ventilation worldwide annually [3, 4]. Since these patients receive hypnotics during the most fragile period of their life, even a small benefit or harm of these drugs would represent a public health issue. However, no megatrial comparing different anesthetics exists, and large randomized trials performed so far did not provide a definitive answer due to possible confounders and study design. As a result, anesthesiologists and intensivists choose the hypnotic agents based on pharmacokinetic and pharmacodynamic properties, pathophysiology, and surrogate endpoints such as hemodynamic parameters, mild symptoms and complications (e.g., postoperative nausea and vomiting), and costs. However, when pooling all available randomized evidence, it is possible to identify even subtle survival differences of each anesthetic agent in the existing literature.

The possible role of sedative techniques on survival is already supported by clinical trials and guidelines. In fact, although mechanically ventilated patients commonly receive hypnotic agents to enhance synchronicity or prevent the risk of agitation-related complications [28, 29], current evidence supports that reducing exposure to hypnotic agents can improve clinical outcomes [30–32]. International guidelines suggest the use of light sedation compared to deep sedation in critically ill patients [30], and two RCTs showed that daily sedation interruption reduced the duration of ventilation compared to no sedation interruption [31, 32]. In addition to sedation depth, the choice of hypnotics may make a difference. A recent network meta-analysis documented that propofol prolonged the duration of mechanical ventilation and increased the risk of delirium compared to dexmedetomidine [33]. Therefore, it would be plausible to expect that the use of hypnotic agents can affect patient outcomes.

The present manuscript can be compared with our previous meta-analysis on propofol, which was published in 2015 and included 133 studies randomizing 14,516 patients [34]. While in the 2015 meta-analysis, we found only a trend toward an increased mortality in patients receiving propofol versus any comparator (5.0% vs. 4.5%; RR = 1.05; 95% CI, 0.93–1.18), in the present manuscript, the increased number of studies and more than doubled patients allowed us to find a statistically significant difference (5.2% vs. 4.3%; RR = 1.10; 95% CI, 1.01–1.20; p = 0.03). The inclusion of pediatric the population did not affect the findings. Since we understood that pediatric patients can be more affected by the detrimental effects of propofol, we decided to included them, but the mortality differences in the subgroup of pediatric patients did not reach a statistically significance.

Although the comparison between volatile agents and total intravenous anesthetics has been extensively investigated over the decades, no established evidence in terms of survival exists. The cardioprotective effects of volatile anesthetics were well described, especially in patients undergoing cardiac surgery [12, 13]. Possible beneficial survival effects of volatile anesthetics in cardiac surgical settings were suggested in several meta-analyses [15, 16, 35, 36]. Among 252 studies included in our meta-analysis, 172 assessed volatile agents as comparator. The subgroup analysis of studies using volatile agents as comparator found a statistically significant mortality increase in patients randomized to propofol, which contributed to the significant mortality difference in the overall analysis. Of note, the recent largest RCT found no difference in mortality or other organ dysfunction between volatile and total intravenous anesthesia [37]. We hypothesize that the use of propofol in the majority of patients who were randomized to the volatile group blunted the detrimental effect of propofol on survival in this trial [37]. In our meta-analysis, several cardiac surgery trials randomized only intraoperative hypnotic agents and few of them used propofol in the propofol and comparator arms after surgery [38–40], which might attenuate mortality increase by propofol in the cardiac surgery subgroup.

Several observational studies and a meta-analysis suggested propofol could increase recurrence-free survival compared to volatile anesthesia in cancer patients [41–43]. However, the present meta-analysis found an increased mortality risk in the propofol arm among patients undergoing non-cardiac surgery (mostly oncologic surgery), even though the difference was not statistically significant. Given that previous evidence was mainly based on non-randomized studies and our analysis includes only randomized trials, the available evidence does not support propofol in cancer surgery while waiting for ongoing large randomized trials [44, 45].

We did not find a significant mortality difference in the subgroup analysis of ICU studies only. There are several possible explanations for why the detrimental effect of propofol was mitigated in ICU settings (1.7% absolute risk increase and 13% non-significant relative risk increase in mortality in the propofol group). ICU patients are generally heterogeneous in terms of chronic comorbidities and acute pathology that led to their admission to intensive care, and their overall mortality is higher than other populations. These characteristics make it difficult to detect a mortality difference in randomized trials [46]. Propofol is associated with a shorter duration of mechanical ventilation than benzodiazepines, which might result in less ventilator-acquired pneumonia and better clinical outcomes [30]. In recent large randomized studies on propofol in intensive care patients, mortality was a secondary end-point [47–49], which might explain a non-significant difference.

Although the detrimental effect of propofol was expected to be remarkable in studies not using propofol in the comparator, the RR for mortality was larger in studies where propofol bolus was used in the comparator than in those without propofol in the comparator. One possible explanation might be that hypnotic agents used in the propofol arm were more detrimental than propofol as an induction agent in the comparator arm, which confounded the increased mortality risk of propofol. For example, etomidate is one of the most common induction agents and may worsen mortality mainly due to adrenal insufficiency [50].

The detrimental effect of propofol on survival documented in the present meta-analysis is not attributable to immediate or acute reactions such as allergic reactions. In the literature, we identified four possible mechanisms of action which might explain the detrimental effect of propofol on survival. First, propofol can cause “propofol infusion syndrome” with metabolic acidosis, rhabdomyolysis, hyperlipidemia, and hepatomegaly [8]. A high-dose (> 4 mg/kg/h) and long-term (> 48 h) administration are well-known risk factors. Despite its wide recognition, there are still recent reports of propofol infusion syndrome in intensive care settings [51]. Second, propofol can increase the risk of infection due to its lipophilic nature supporting bacterial growth at room temperature [52], demonstrated by 58 reports of propofol-related infection outbreaks during 1989–2014 [53]. This might still be an issue even if ethylenediamine tetraacetic acid-containing propofol started to be used to inhibit the bacterial growth around 2000 [9, 54], and the methods of preparation and administration have been improved to reduce the risk of contamination [43]. In fact, the risk of bacterial growth was reduced but not eliminated and low-quality of practice (e.g., preparing perfusions in advance) might be associated with bacterial contamination [53]. Nonetheless, our sensitivity analyses showed that the increased mortality risk was constant over the decades. Third, propofol might inhibit the organ protective properties of other interventions (e.g., volatile organ protection, remote ischemic preconditioning). As a phenolic derivative, propofol acts as a free radical scavenger [55] and in animal models it inhibits preconditioning in rabbits [56]. Since this mechanism is not demonstrated in elderly patients, further research is necessary to confirm this mechanism of action in clinical settings. Fourth, propofol can induce hemodynamic instability through vasodilation and reduced myocardial contractility [57]. Since even a short duration of hypotension is associated with mortality [58, 59], hemodynamic impairment could contribute to the mortality increase.

We also acknowledge that several beneficial effects of propofol exist including but not limited to antioxidant properties, suppression of apoptosis, and anti-inflammatory effects, all of which might have organ protective effects [60–64].

### Limitations

Our study has limitations. First, the risk of bias was judged high in 16% of included studies but notably the statistically significant increase in mortality was confirmed after excluding studies with a high risk of bias. Further concerns for risk of bias were mainly due to the technical impossibility of a double-blind design in such complicated settings (e.g., perioperative and ICU). Second, we acknowledge that meta-analytic findings are often considered hypothesis-generating especially because they often include several small studies. In addition, our meta-analysis pooled mortality data from very different settings (e.g., anesthesia in several surgeries versus deep or light sedation in the ICU) and at different timepoints. However, more than half of worldwide ICU admissions pertain to postoperative patients [65–67] and all our subgroup analyses and in particular the one including large studies only followed the same magnitude and direction of the primary analysis. Since the 252 studies included in our meta-analysis reported mortality at 17 different follow-up times, restriction to only one predefined timepoint would exclude most studies. We therefore used mortality at the longest follow-up available, which is an accepted approach to increase the number of eligible studies without influencing pooled point estimates of the effects at the study level [23]. Furthermore, the symmetrical funnel plot allowed us to exclude small studies publication bias. Third, many included studies reported no death in at least one arm, leading to a wide confidence interval of the treatment effect. However, the robustness of our findings was supported by the low heterogeneity and consistency of the primary and subgroup analyses. Fourth, we did not perform the predetermined subgroup analysis on duration of drug infusion, but these data were not available to be extracted and we decided to introduce a subgroup analysis with ICU studies only.

### Future perspectives

Since not all previously conducted large RCTs limited the choice of intravenous hypnotics to propofol and avoided the use of propofol in the comparator arm, future trials should compare propofol with a propofol-free anesthesia strategy to confirm the provocative and hypothesis-generating findings of the present meta-analysis. Such a megatrial should be multinational, investigator-initiated, and no-profit to minimize the risk of bias and increase the external validity. Although ethical issues of conducting a clinical trial to test harm need to be considered, it is a matter of fact that propofol is already administered to hundreds of millions of patients annually, which might correspond to thousands of deaths based on our findings.

While waiting for large, randomized trials, we suggest physicians to consider alternative hypnotic agents when available and feasible, to implement hypnotic rotation strategies in the ICU, and to attempt propofol dose reduction whenever is possible.

## Conclusions

This meta-analysis of randomized trials suggests that propofol increases mortality by 10% when compared to other hypnotic agents. Large-scale prospective studies are warranted to confirm our findings.

## Supplementary Information

**Additional file 1.** Search strategy, supplemental Tables, and supplemental Figures.

**Additional file 2.** Complete reference list of included studies.

## Data Availability

We collected the summary data from published randomized trials. This published article and its supplementary files include all the data generated or analyzed for this study. Further information is available from the corresponding authors upon reasonable request.

## Abbreviations

CI: Confidence interval
ICU: Intensive care unit
NNH: Number needed to harm
PRISMA: Preferred Reporting Items for Systematic Reviews and Meta-Analyses
RCT: Randomized controlled trial
RR: Risk ratio
TSA: Trial sequential analysis
