# Supplementary material for: Propofol and survival: an updated meta-analysis of randomized clinical trials
Source: Crit Care. 2023 Apr 12;27:139. doi: 10.1186/s13054-023-04431-8 (PMC10099692; doi:10.1186/s13054-023-04431-8)
Supplement: Supplementary file 1 — Additional file 1. Search strategy, supplemental Tables, and supplemental Figures. [file 13054_2023_4431_MOESM1_ESM.docx]

**Additional file 1**

**Propofol and survival. An updated meta-analysis of randomized clinical trials.**

**Authors**

Yuki Kotani, MD, Alessandro Pruna, MD, Stefano Turi, MD, Giovanni Borghi, MD, Todd C Lee, MPH, Alberto Zangrillo, MD, Giovanni Landoni, MD, Laura Pasin, MD

**Table of contents**

Search strategy for systematic literature review 4

Fig. S1. Forest plot for mortality in the overall population. 5

Fig. S2. Bubble plots for mortality over the decades. 6

Fig. S3. Forest plot for mortality in the surgical setting. 7

Fig. S4. Forest plot for mortality in the cardiac surgery setting. 8

Fig. S5. Forest plot for mortality in the non-cardiac surgery setting. 9

Fig. S6. Forest plot for mortality in the intensive care unit setting. 10

Fig. S7. Forest plot for mortality in adult patients 11

Fig. S8. Forest plot for mortality in pediatric patients. 12

Fig. S9. Forest plot for mortality in studies where volatile agents were used as comparator. 13

Fig. S10. Forest plot for mortality in studies where intravenous hypnotics were used as comparator. 14

Fig. S11. Forest plot for mortality in studies where miscellaneous anesthetics were used as comparator. 15

Fig. S12. Forest plot for mortality in studies where propofol was used as bolus in the comparator arm. 16

Fig. S13. Forest plot for mortality in studies where propofol was not used in the comparator arm. 17

Fig. S14. Forest plot for mortality in large studies enrolling ≥500 patients. 18

Fig. S15. Forest plot for mortality in small studies enrolling <500 patients. 19

Fig. S16. Forest plot for mortality in studies where mortality was ≥4.5% in the comparator arm. 20

Fig. S17. Forest plot for mortality in studies where mortality was <4.5% in the comparator arm. 21

Fig. S18. Forest plot for mortality excluding studies with high risk of bias. 22

Fig. S19. Forest plot for mortality including studies published after 2005. 23

Fig. S20. Forest plot for mortality with the Peto method. 24

Fig. S21. Forest plot for mortality using a random-effects model. 25

Fig. S22. Forest plot for mortality including studies reporting hospital or long-term mortality. 26

Fig. S23. Probability density functions for combined posterior distributions of the difference in mortality in the cardiac surgery setting. 27

Fig. S24. Probability density functions for combined posterior distributions of the difference in mortality in the non-cardiac surgery setting. 28

Fig. S25. Probability density functions for combined posterior distributions of the difference in mortality in the intensive care unit setting. 29

Fig. S26. Probability density functions for combined posterior distributions of the difference in mortality for all studies with a binomial model. 30

Fig. S27. Probability density functions for combined posterior distributions of the difference in mortality in cardiac surgery settings with a binomial model. 31

Fig. S28. Probability density functions for combined posterior distributions of the difference in mortality in non-cardiac surgery settings with a binomial model. 32

Fig. S29. Probability density functions for combined posterior distributions of the difference in mortality in intensive care settings with a binomial model. 33

Fig. S30. Trial sequential analysis for mortality. 34

Table S1. PRISMA 2020 checklist. 35

Table S2. Timing of mortality and risk of bias assessment of the included studies in order of publication year 38

Table S3. Timepoints of mortality reported as reported in the 252 studies 53

Table S4: Mortality at different timepoints reported in included studies 54

# Search strategy for systematic literature review

PubMed

propofol AND (randomized controlled trial[pt] OR controlled clinical trial[pt] OR randomized controlled trials[mh] OR random allocation[mh] OR double-blind method[mh] OR single-blind method[mh] OR clinical trial[pt] OR clinical trials[mh] OR (clinical trial[tw] OR ((singl*[tw] OR doubl*[tw] OR trebl*[tw] OR tripl*[tw]) AND (mask*[tw] OR blind[tw])) OR (latin square[tw]) OR placebos[mh] OR placebo*[tw] OR random*[tw] OR research design[mh:noexp] OR follow-up studies[mh] OR prospective studies[mh] OR cross-over studies[mh] OR control*[tw] OR prospectiv*[tw] OR volunteer*[tw]) NOT (animal[mh] NOT human[mh]) NOT (comment[pt] OR editorial[pt] OR meta-analysis[pt] OR practice-guideline[pt] OR review[pt]))

CENTRAL

#1 propofol

#2 MeSH descriptor: [Propofol] explode all trees

#3 MeSH descriptor: [Hypnotics and Sedatives] explode all trees

#4 #1 or #2 or #3

ClinicalTrials.gov

https://clinicaltrials.gov/

Advanced Search >

Study type: All studies

Study Results: All studies

Intervention: Propofol

# Fig. S1. Forest plot for mortality in the overall population.


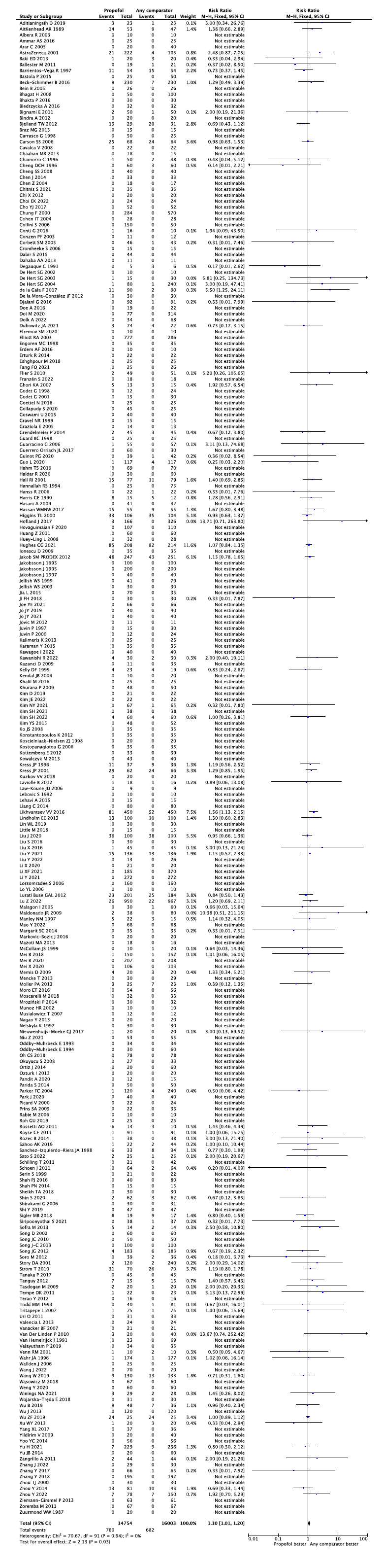


# Fig. S2. Bubble plots for mortality over the decades.


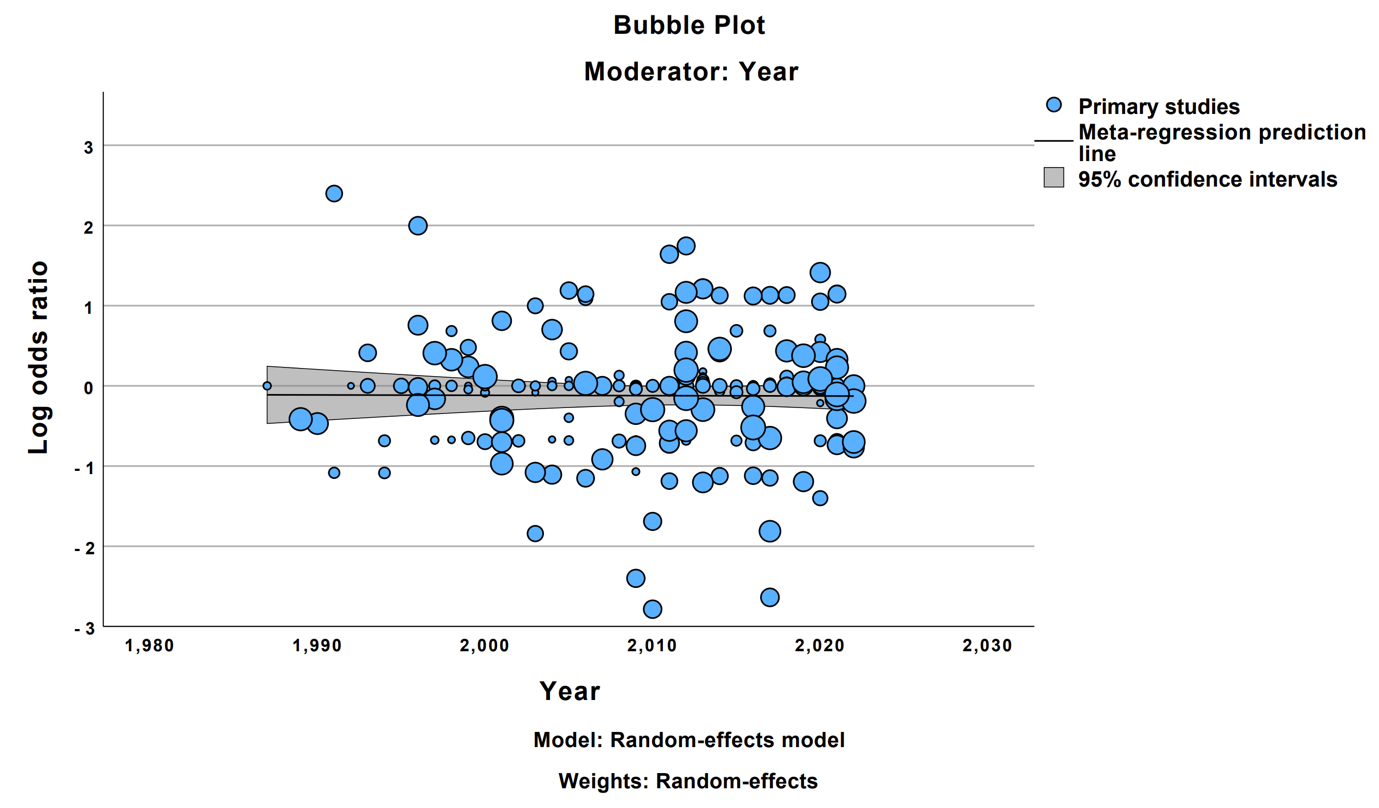


# Fig. S3. Forest plot for mortality in the surgical setting.


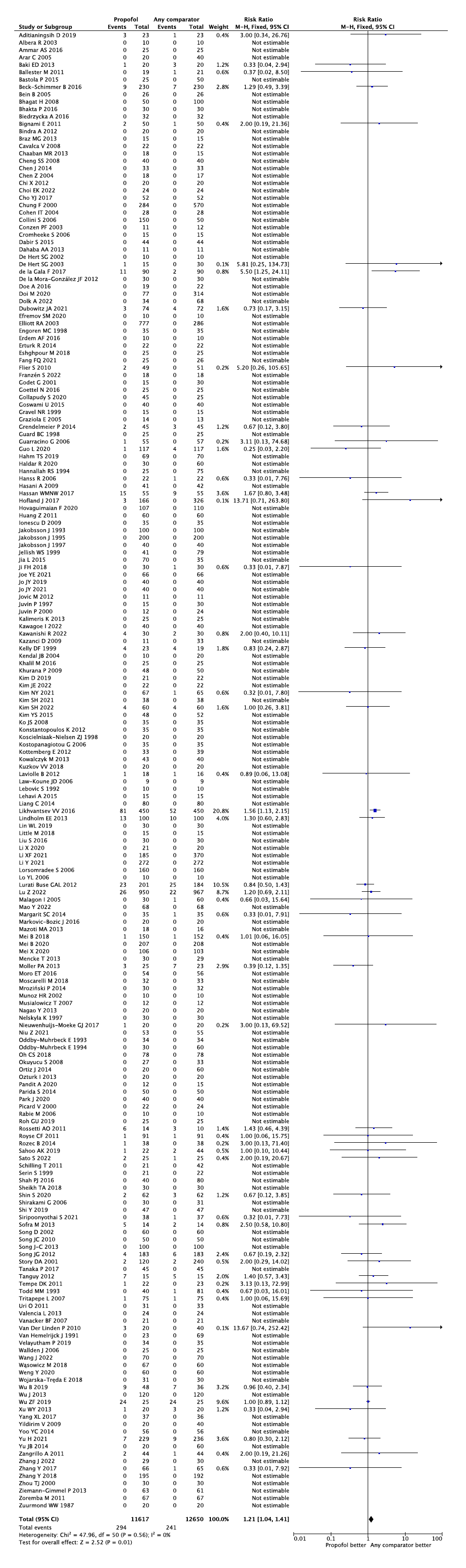


# Fig. S4. Forest plot for mortality in the cardiac surgery setting.


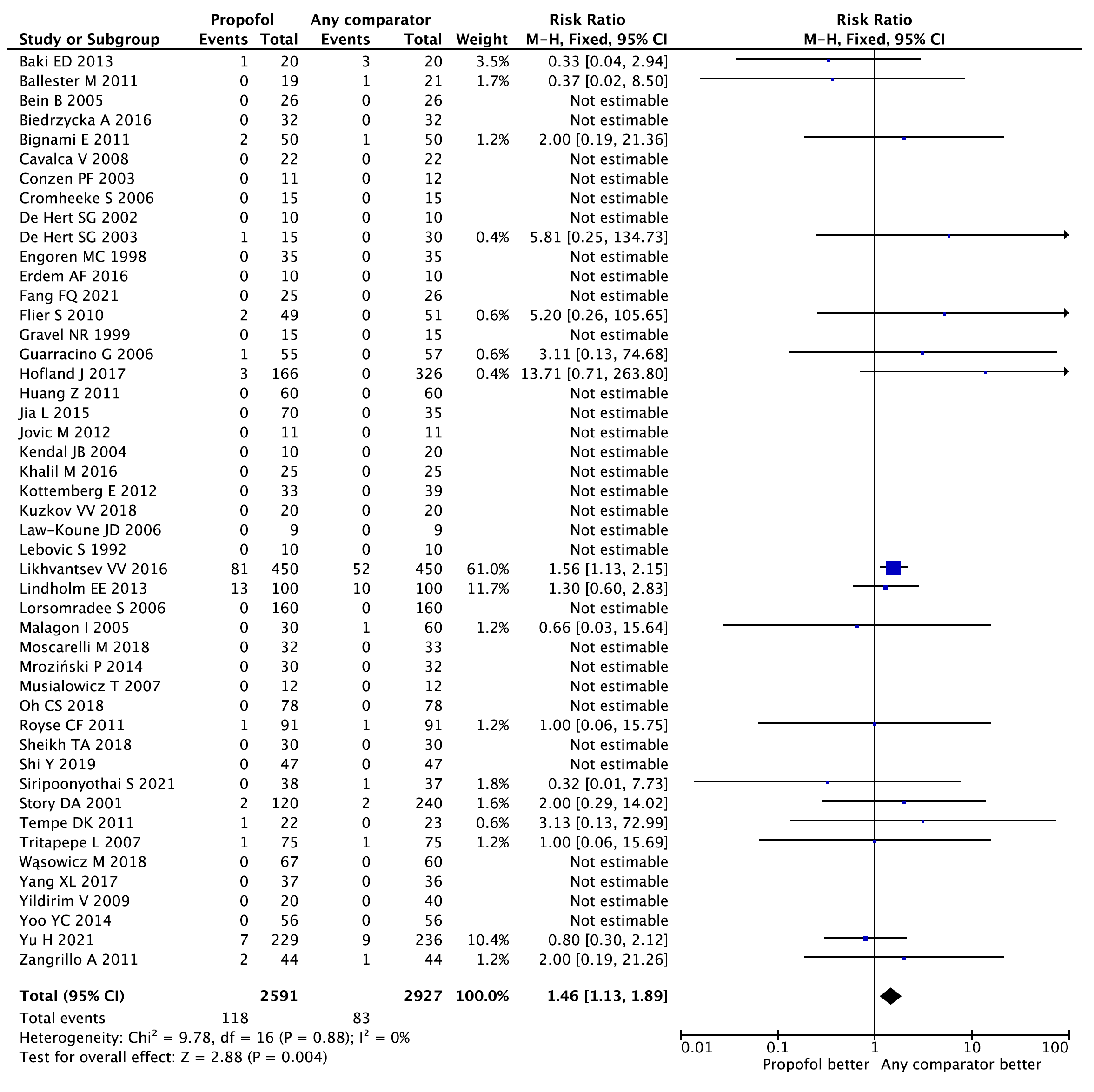


# Fig. S5. Forest plot for mortality in the non-cardiac surgery setting.


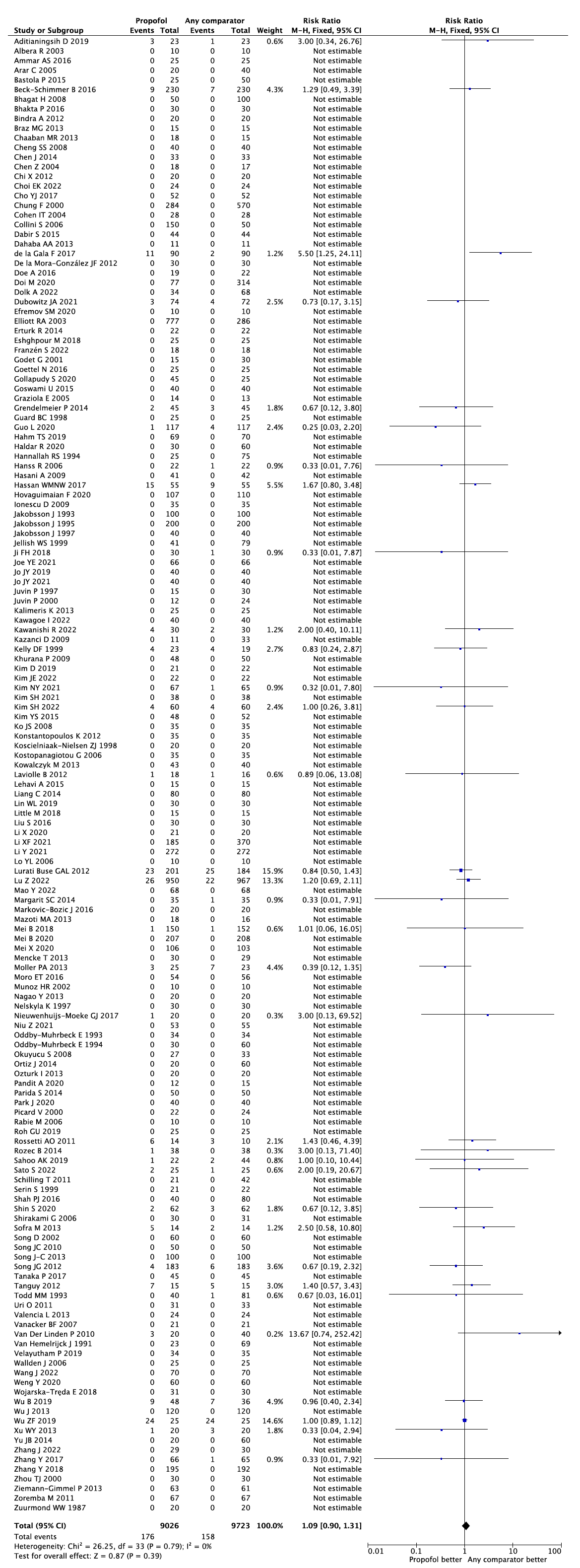


# Fig. S6. Forest plot for mortality in the intensive care unit setting.


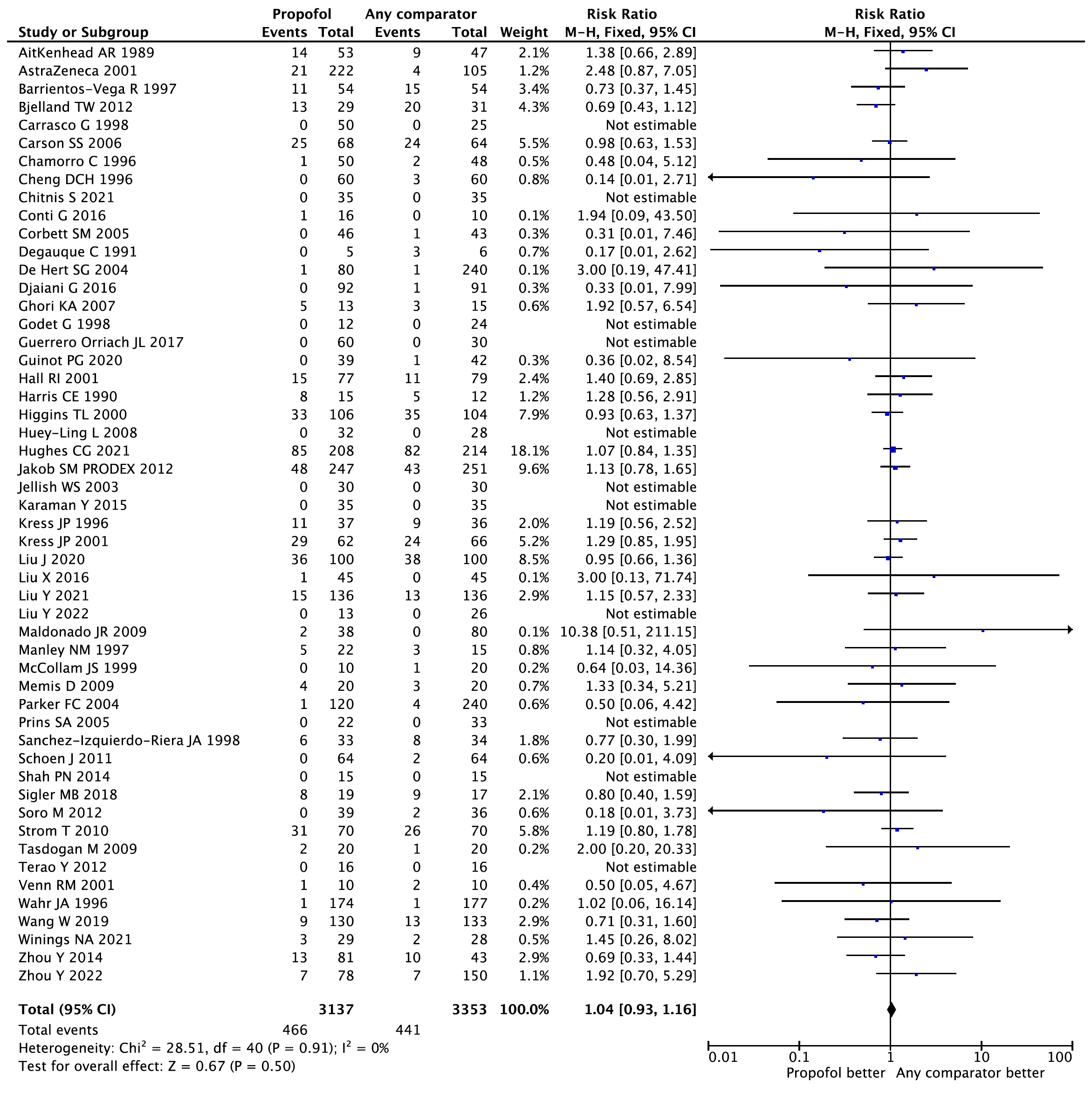


# Fig. S7. Forest plot for mortality in adult patients


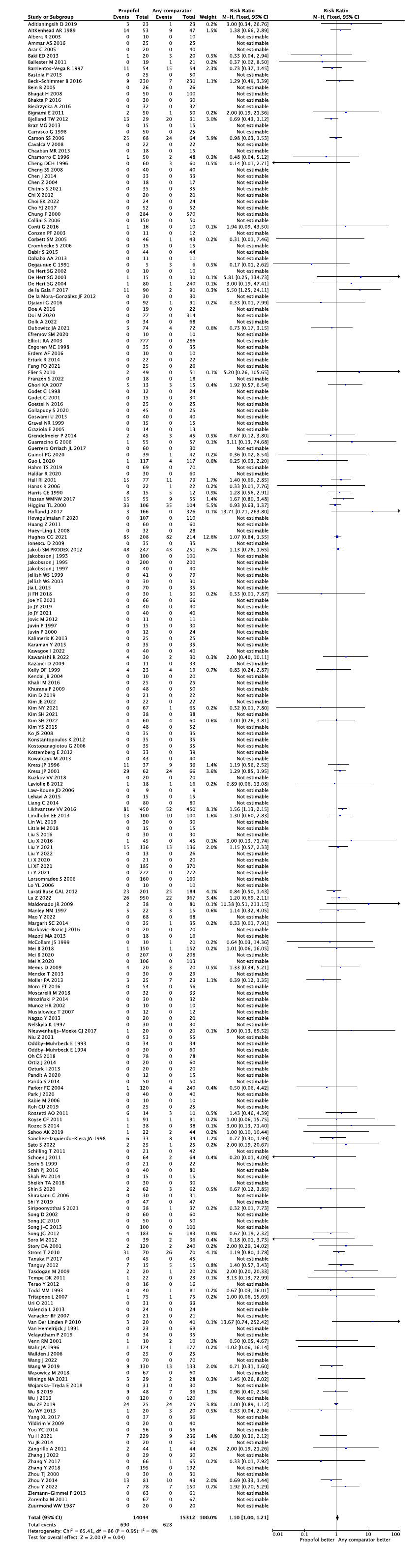


# Fig. S8. Forest plot for mortality in pediatric patients.


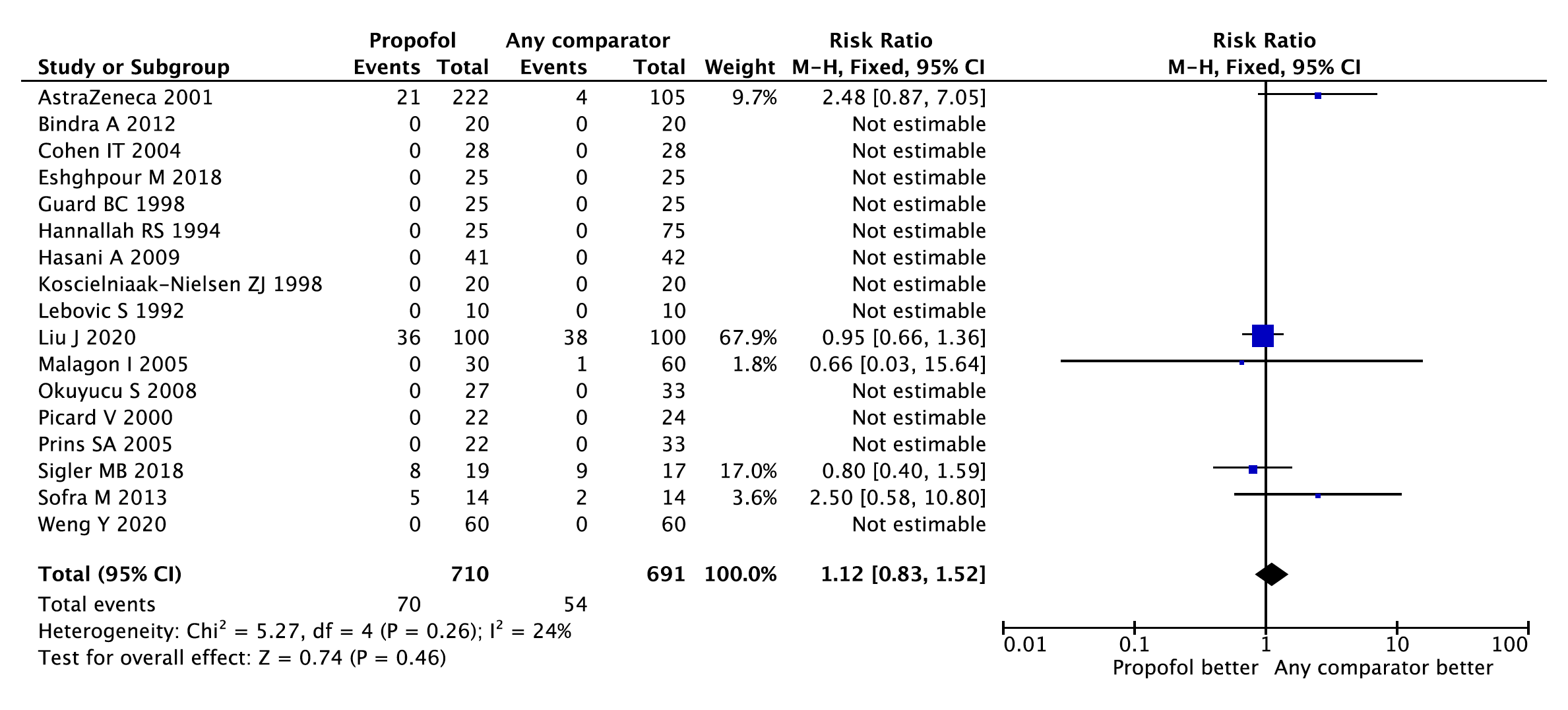


# Fig. S9. Forest plot for mortality in studies where volatile agents were used as comparator.


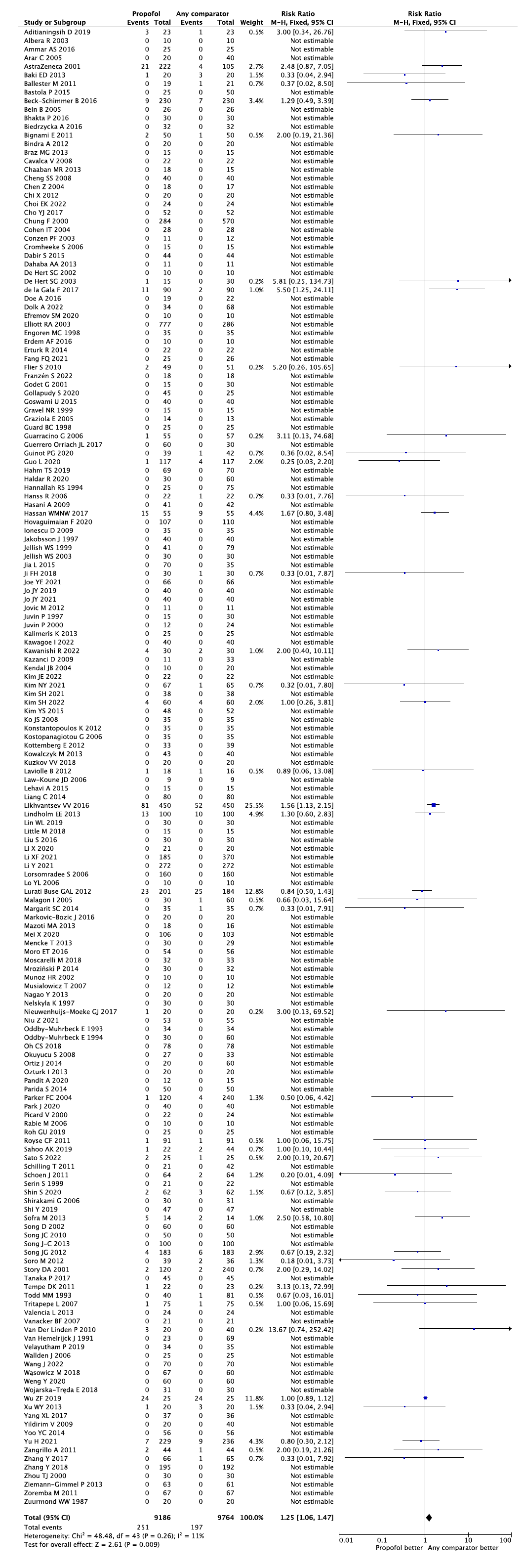


# Fig. S10. Forest plot for mortality in studies where intravenous hypnotics were used as comparator.


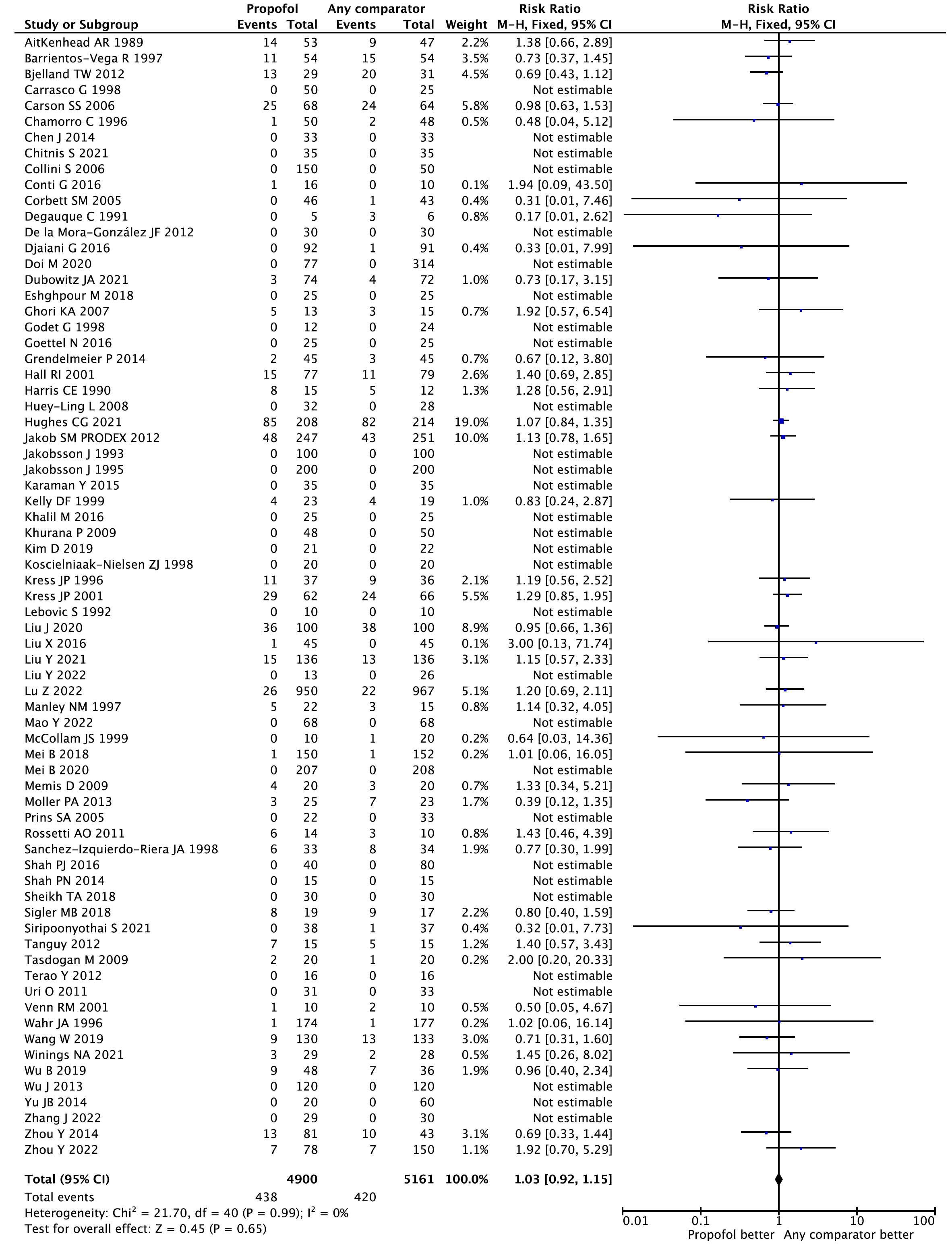


# Fig. S11. Forest plot for mortality in studies where miscellaneous anesthetics were used as comparator.


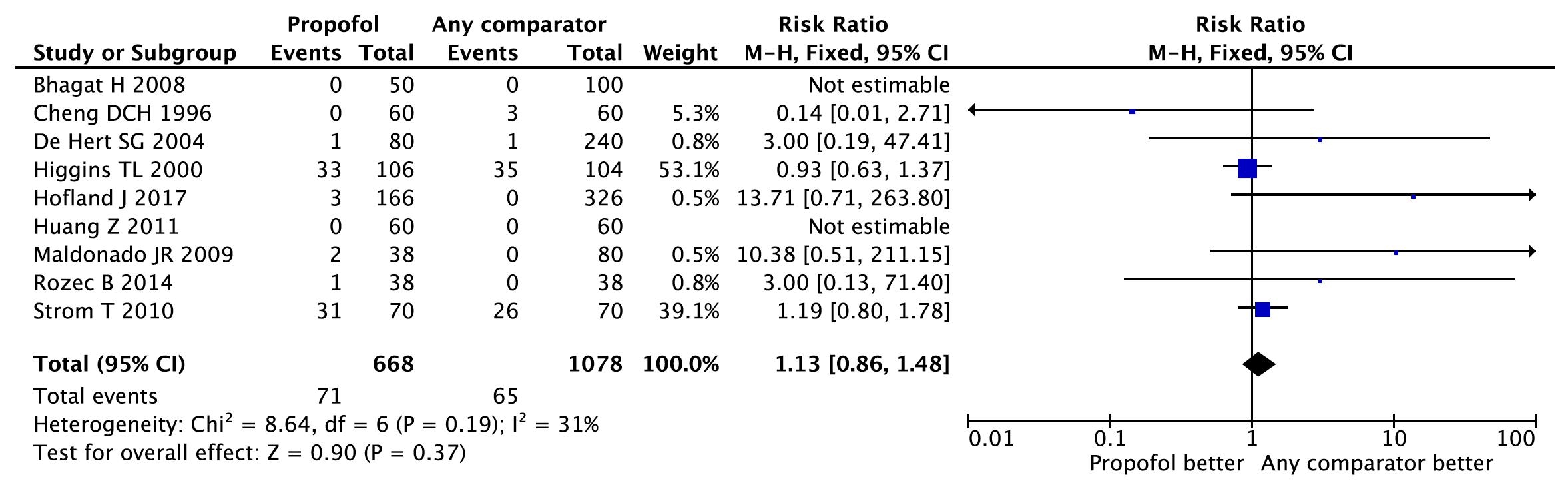


# Fig. S12. Forest plot for mortality in studies where propofol was used as bolus in the comparator arm.


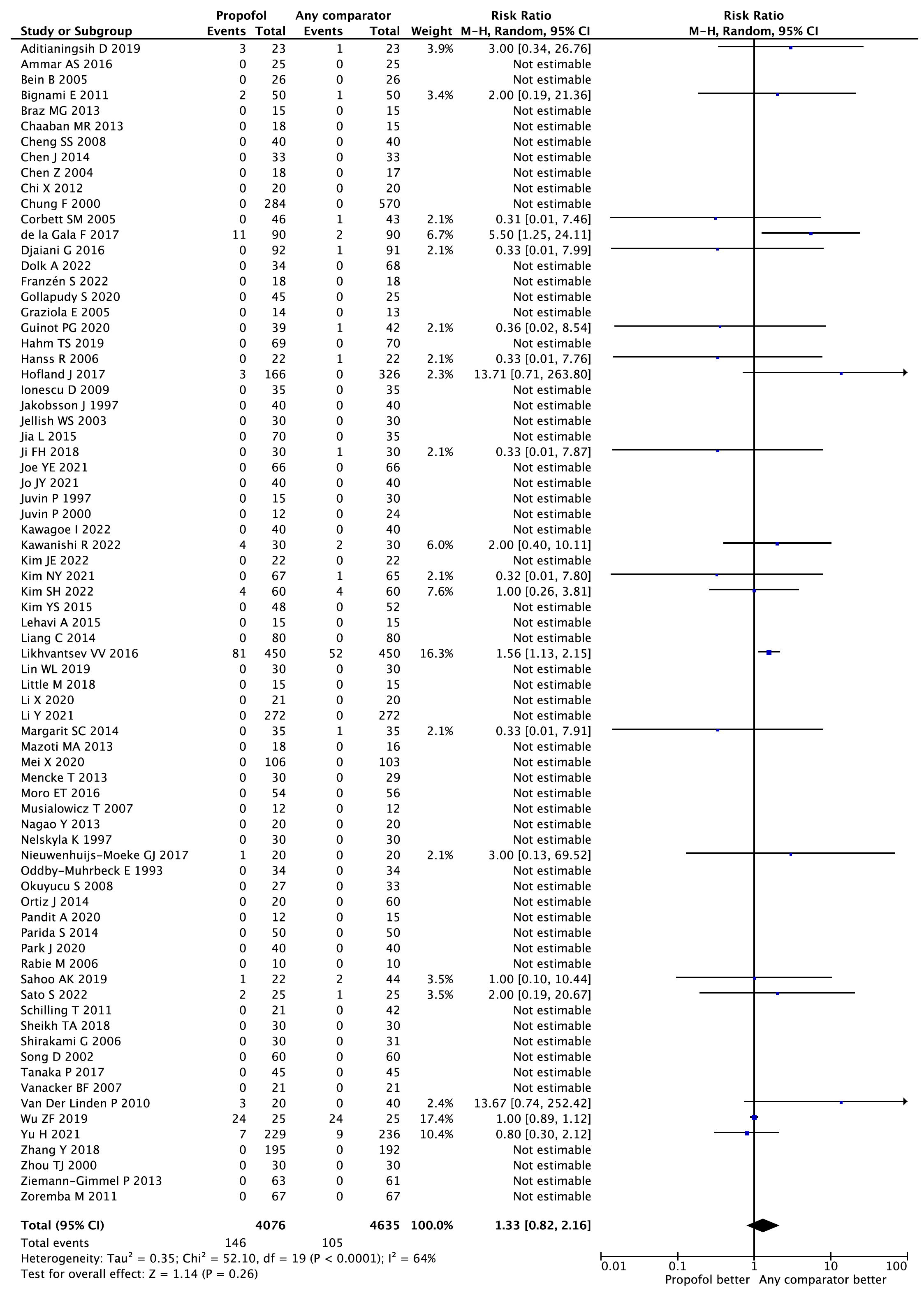


# Fig. S13. Forest plot for mortality in studies where propofol was not used in the comparator arm.


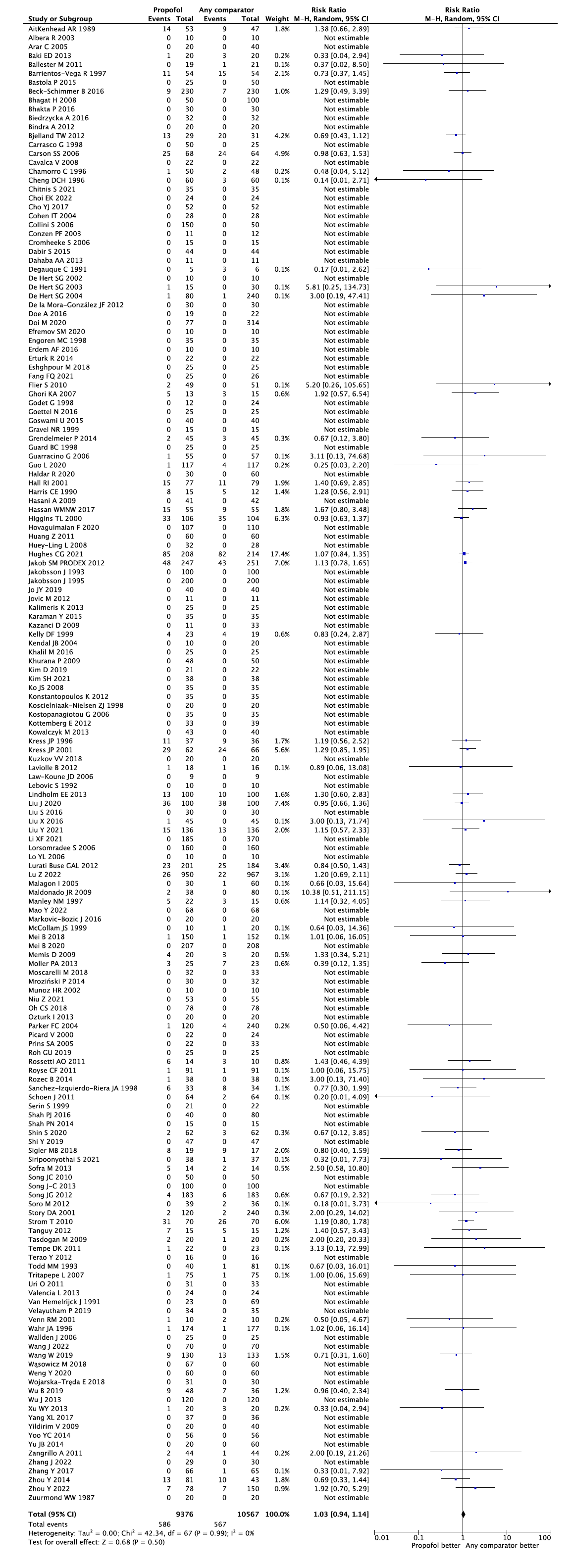


# Fig. S14. Forest plot for mortality in large studies enrolling ≥500 patients.


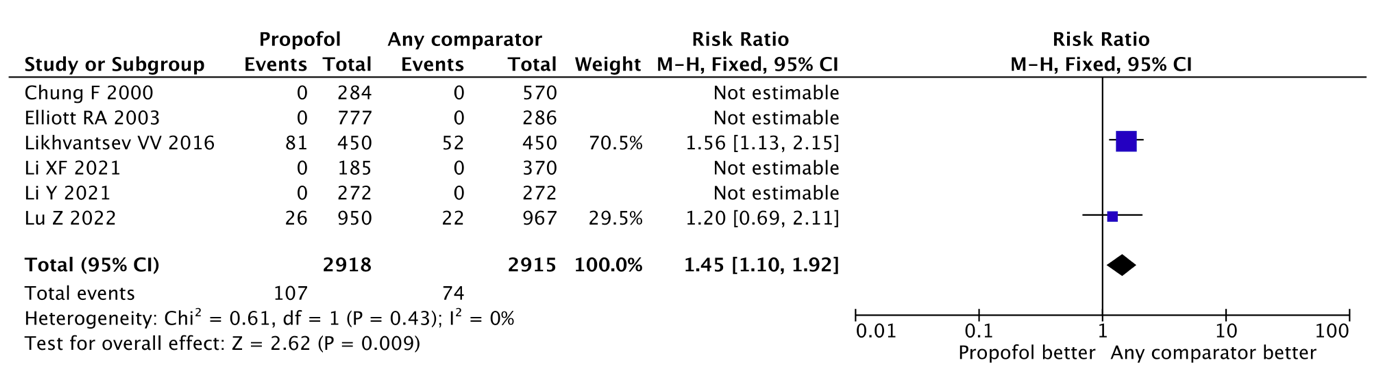


# Fig. S15. Forest plot for mortality in small studies enrolling <500 patients.


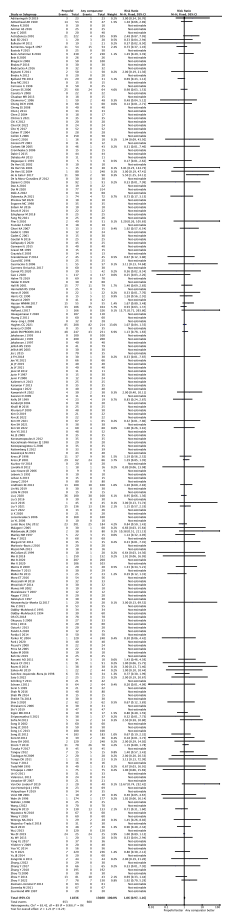


# Fig. S16. Forest plot for mortality in studies where mortality was ≥4.5% in the comparator arm.


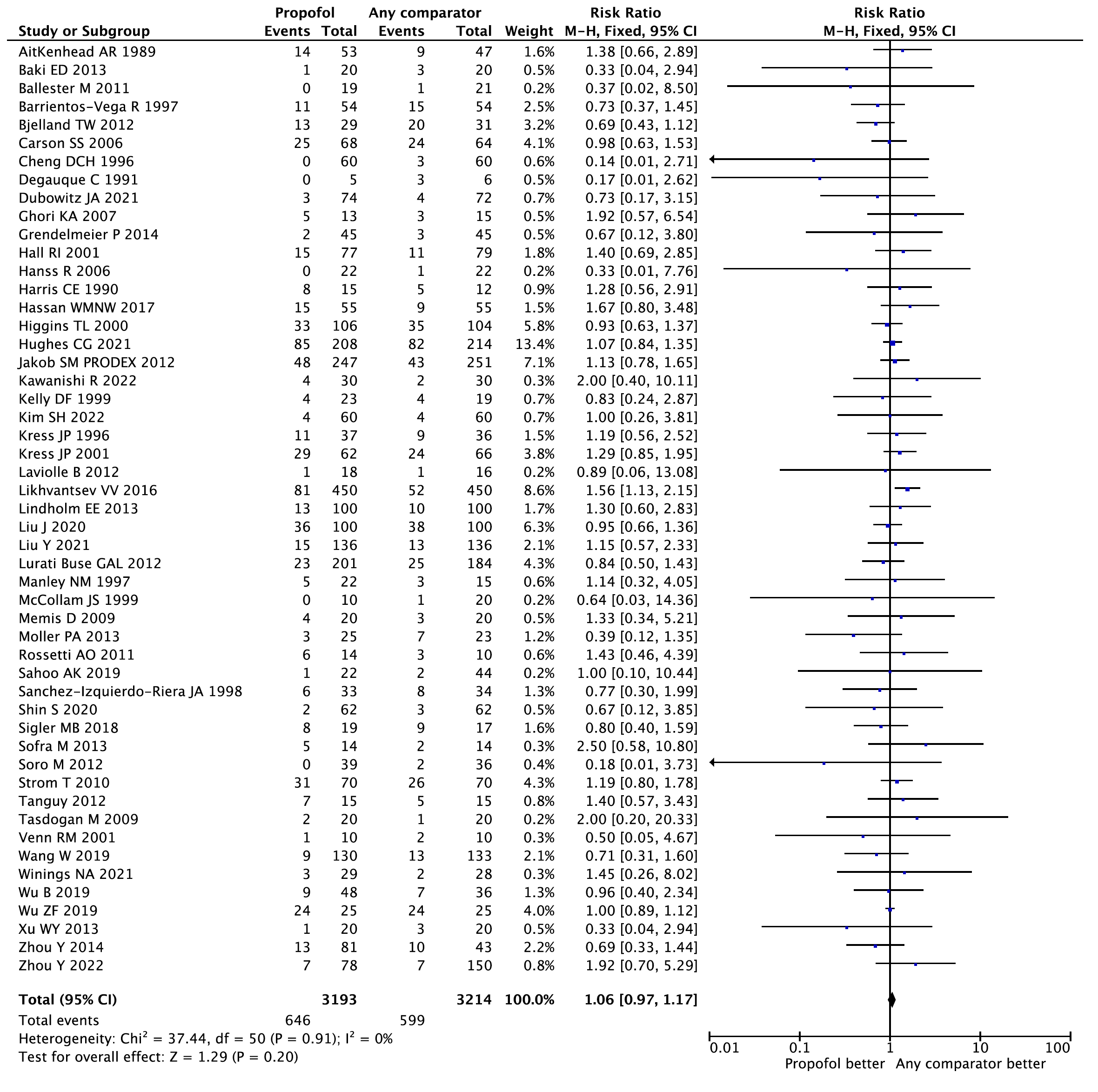


# Fig. S17. Forest plot for mortality in studies where mortality was <4.5% in the comparator arm.


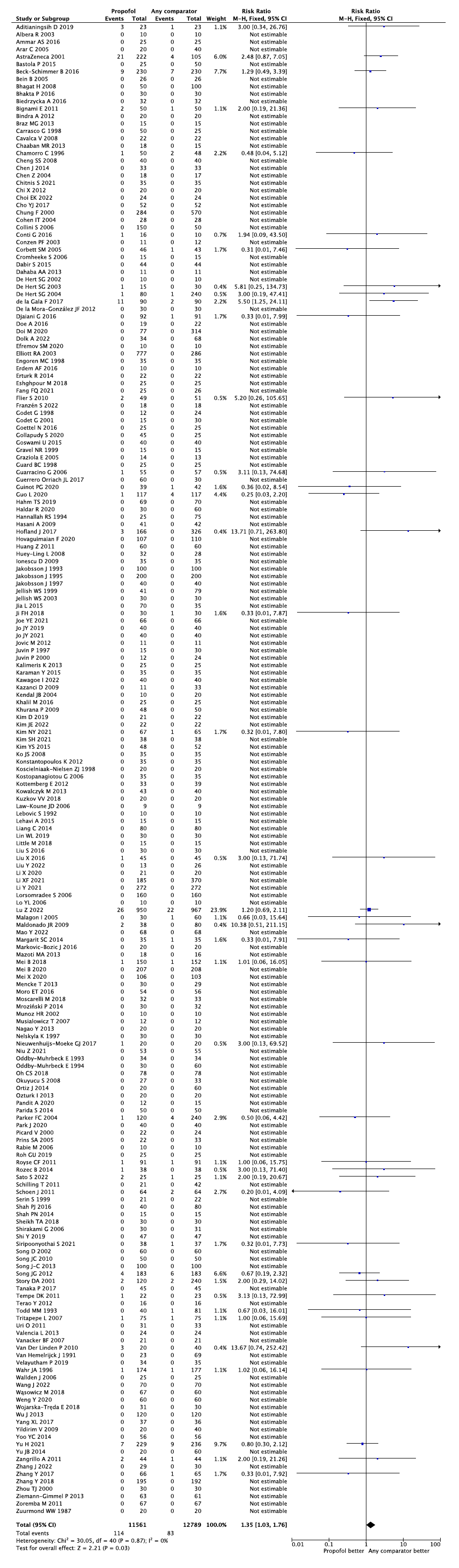


# Fig. S18. Forest plot for mortality excluding studies with high risk of bias.


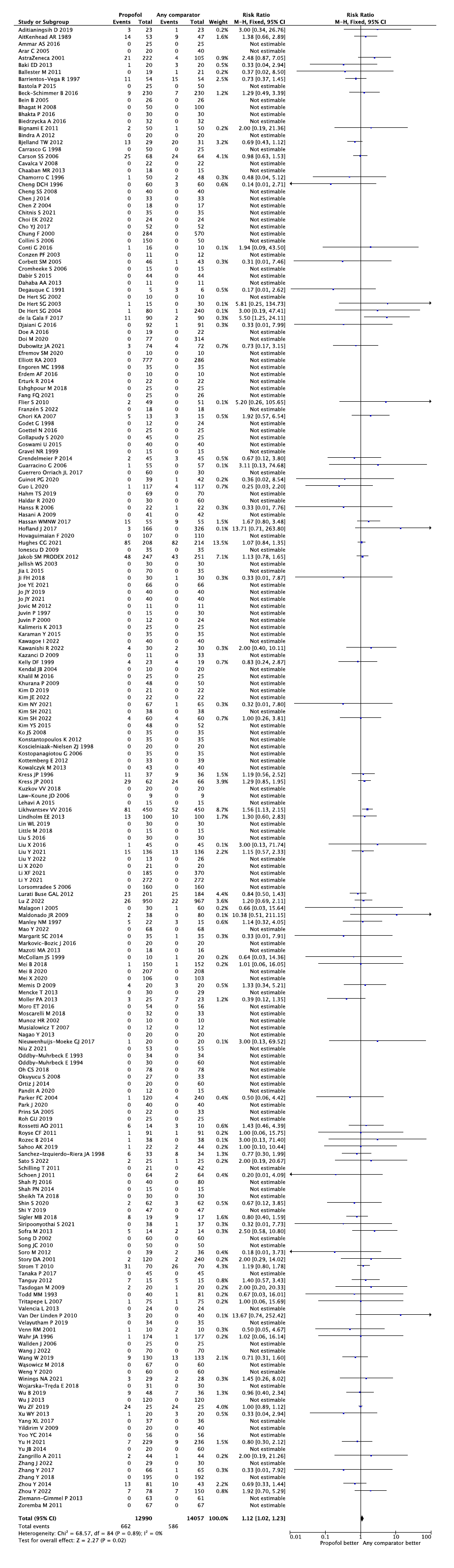


# Fig. S19. Forest plot for mortality including studies published after 2005.

# Fig. S20. Forest plot for mortality with the Peto method.

# Fig. S21. Forest plot for mortality using a random-effects model.

# Fig. S22. Forest plot for mortality including studies reporting hospital or long-term mortality.

# Fig. S23. Probability density functions for combined posterior distributions of the difference in mortality in the cardiac surgery setting.


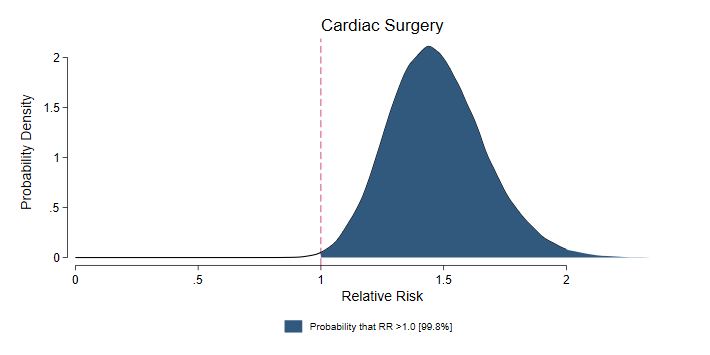


# Fig. S24. Probability density functions for combined posterior distributions of the difference in mortality in the non-cardiac surgery setting.


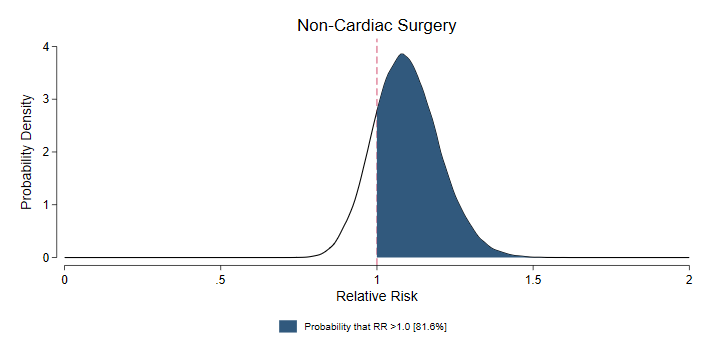


Fig. S25. Probability density functions for combined posterior distributions of the difference in mortality in the intensive care unit setting.


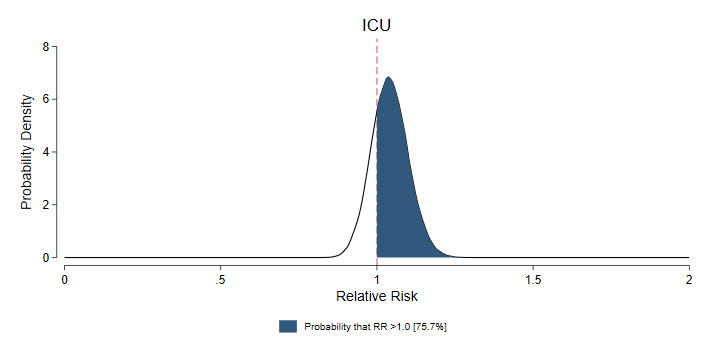


Fig. S26. Probability density functions for combined posterior distributions of the difference in mortality for all studies with a binomial model.


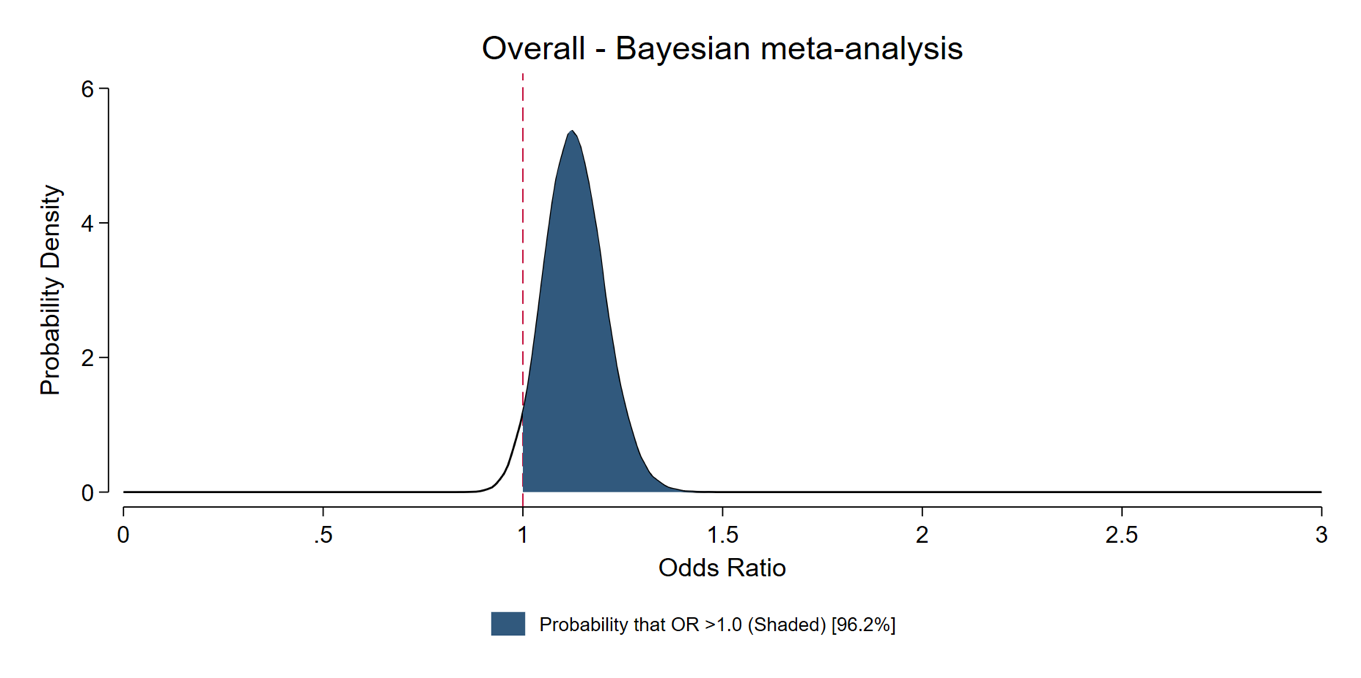


Fig. S27. Probability density functions for combined posterior distributions of the difference in mortality in cardiac surgery settings with a binomial model.

**
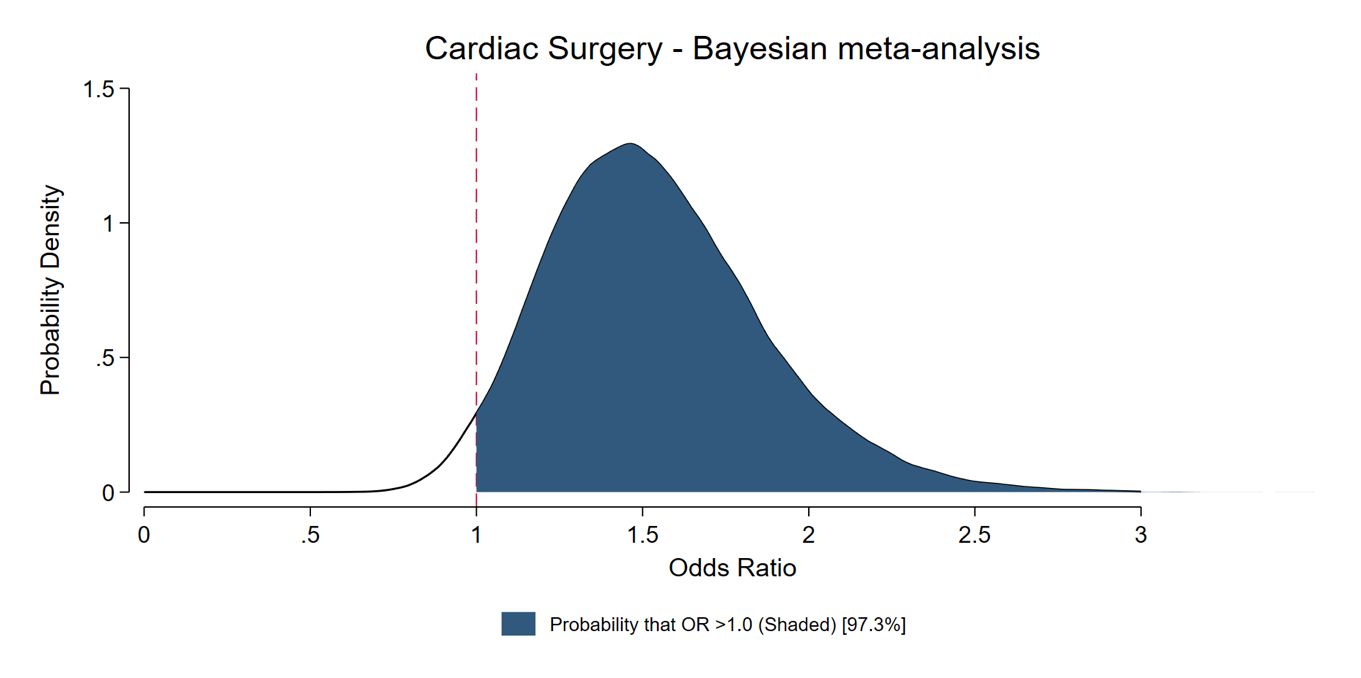
**

Fig. S28. Probability density functions for combined posterior distributions of the difference in mortality in non-cardiac surgery settings with a binomial model.

**
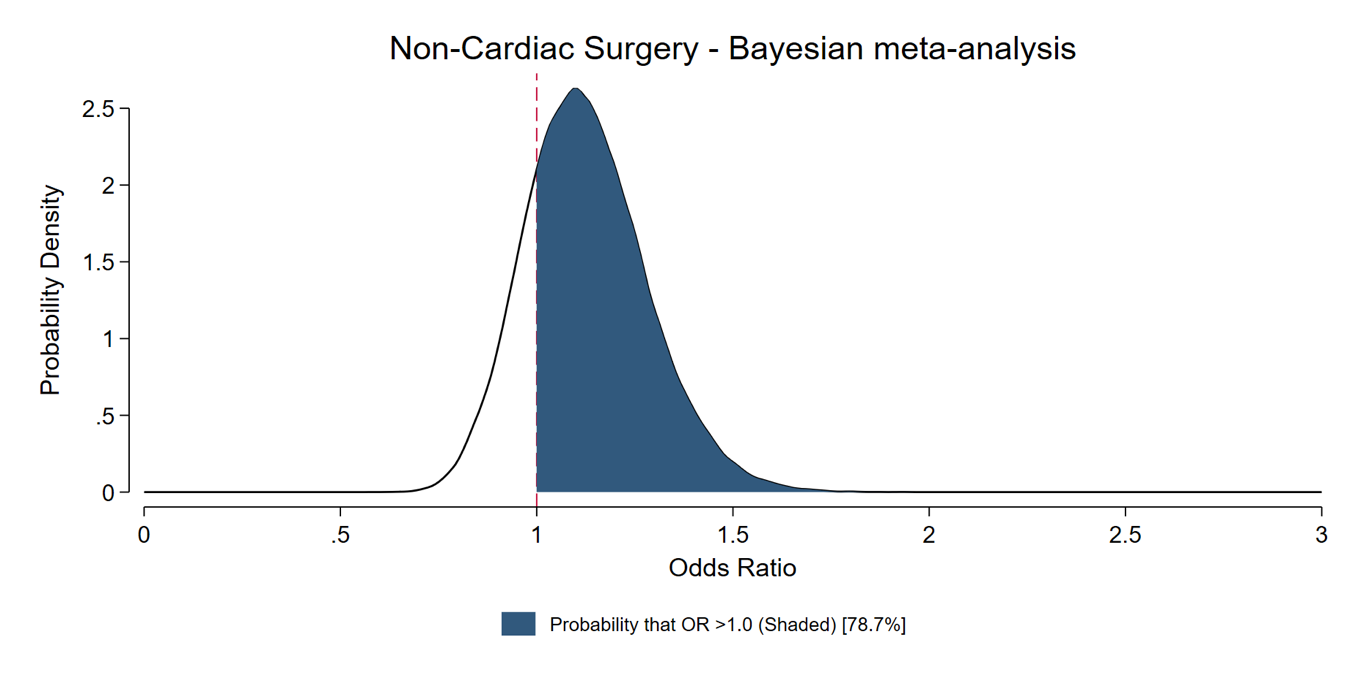
**

Fig. S29. Probability density functions for combined posterior distributions of the difference in mortality in intensive care settings with a binomial model.

**
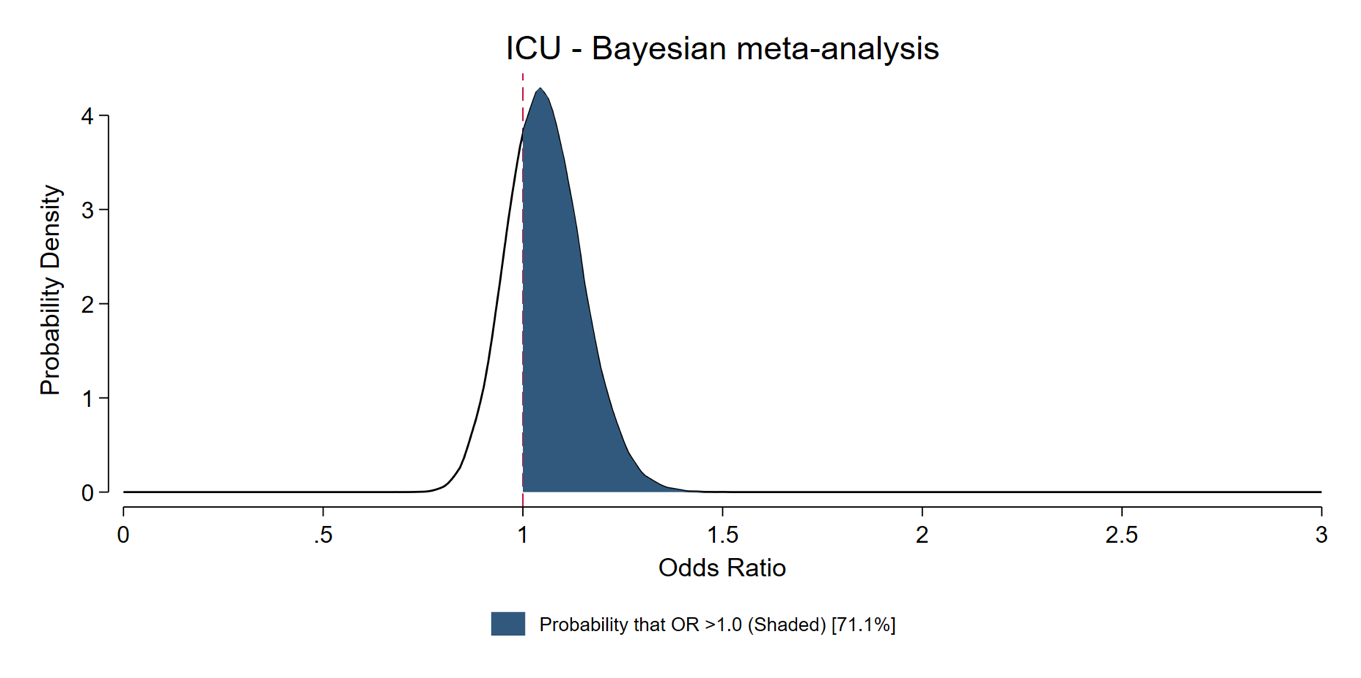
**

# Fig. S30. Trial sequential analysis for mortality.


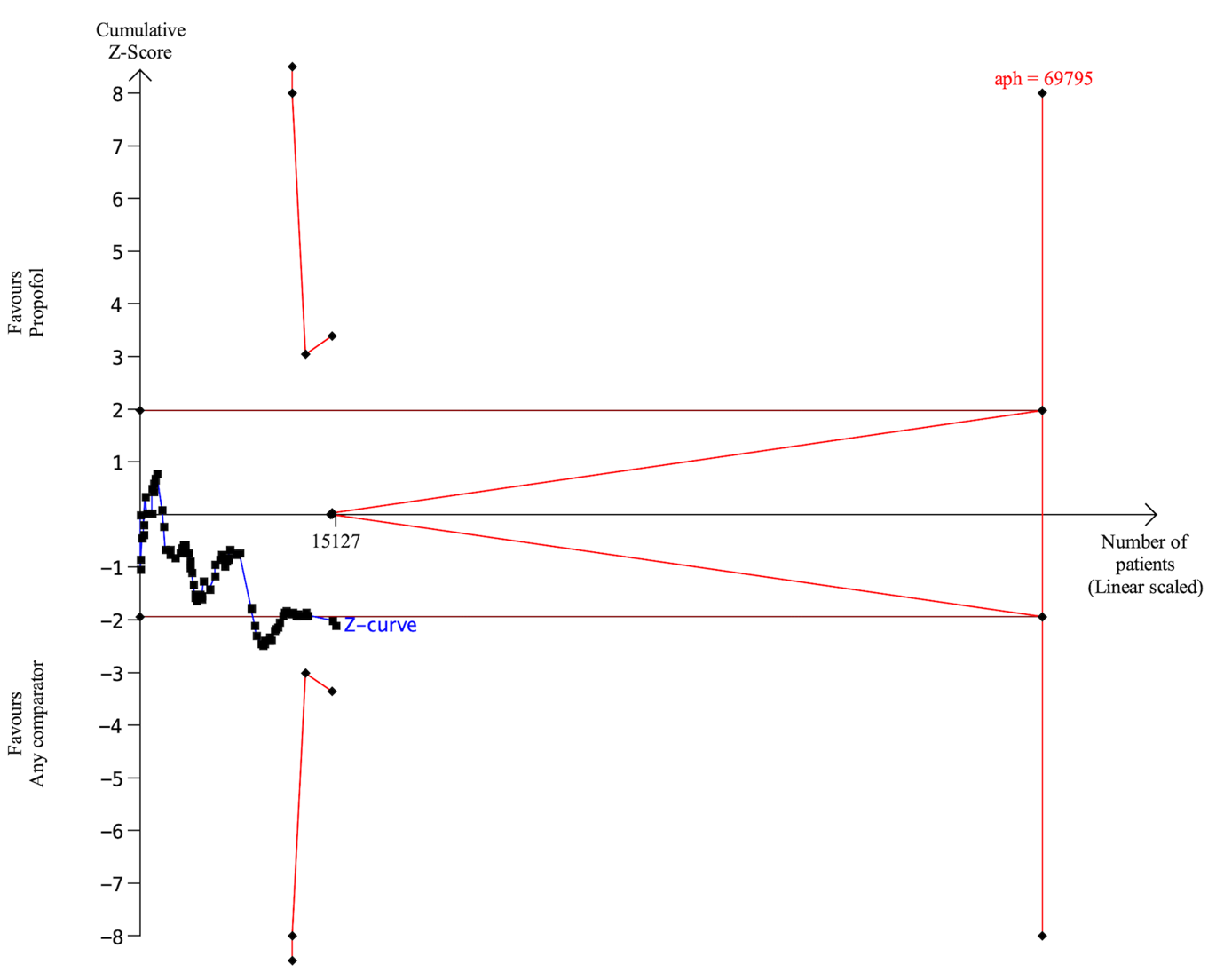


Alpha error = 5%, power = 80%, relative risk increase = 10%, diversity = 0%

#

# Table S1. PRISMA 2020 checklist.

| **Section and Topic** | **Item #** | **Checklist item** | **Location where item is reported** |
| --- | --- | --- | --- |
| **TITLE** | | |  |
| Title | 1 | Identify the report as a systematic review. | 1 |
| **ABSTRACT** | | |  |
| Abstract | 2 | See the PRISMA 2020 for Abstracts checklist. | 3,4 |
| **INTRODUCTION** | | |  |
| Rationale | 3 | Describe the rationale for the review in the context of existing knowledge. | 5 |
| Objectives | 4 | Provide an explicit statement of the objective(s) or question(s) the review addresses. | 6 |
| **METHODS** | | |  |
| Eligibility criteria | 5 | Specify the inclusion and exclusion criteria for the review and how studies were grouped for the syntheses. | 6-7 |
| Information sources | 6 | Specify all databases, registers, websites, organisations, reference lists and other sources searched or consulted to identify studies. Specify the date when each source was last searched or consulted. | 6 |
| Search strategy | 7 | Present the full search strategies for all databases, registers and websites, including any filters and limits used. | Supplementary material |
| Selection process | 8 | Specify the methods used to decide whether a study met the inclusion criteria of the review, including how many reviewers screened each record and each report retrieved, whether they worked independently, and if applicable, details of automation tools used in the process. | 6-7 |
| Data collection process | 9 | Specify the methods used to collect data from reports, including how many reviewers collected data from each report, whether they worked independently, any processes for obtaining or confirming data from study investigators, and if applicable, details of automation tools used in the process. | 7-8 |
| Data items | 10a | List and define all outcomes for which data were sought. Specify whether all results that were compatible with each outcome domain in each study were sought (e.g. for all measures, time points, analyses), and if not, the methods used to decide which results to collect. | 7-8 |
|  | 10b | List and define all other variables for which data were sought (e.g. participant and intervention characteristics, funding sources). Describe any assumptions made about any missing or unclear information. | 7-8 |
| Study risk of bias assessment | 11 | Specify the methods used to assess risk of bias in the included studies, including details of the tool(s) used, how many reviewers assessed each study and whether they worked independently, and if applicable, details of automation tools used in the process. | 7 |
| Effect measures | 12 | Specify for each outcome the effect measure(s) (e.g. risk ratio, mean difference) used in the synthesis or presentation of results. | 7-8 |
| Synthesis methods | 13a | Describe the processes used to decide which studies were eligible for each synthesis (e.g. tabulating the study intervention characteristics and comparing against the planned groups for each synthesis (item #5)). | 7-8 |
|  | 13b | Describe any methods required to prepare the data for presentation or synthesis, such as handling of missing summary statistics, or data conversions. | 7-8 |
|  | 13c | Describe any methods used to tabulate or visually display results of individual studies and syntheses. | 7-8 |
|  | 13d | Describe any methods used to synthesize results and provide a rationale for the choice(s). If meta-analysis was performed, describe the model(s), method(s) to identify the presence and extent of statistical heterogeneity, and software package(s) used. | 7-8 |
|  | 13e | Describe any methods used to explore possible causes of heterogeneity among study results (e.g. subgroup analysis, meta-regression). | 7-8 |
|  | 13f | Describe any sensitivity analyses conducted to assess robustness of the synthesized results. | 7-8 |
| Reporting bias assessment | 14 | Describe any methods used to assess risk of bias due to missing results in a synthesis (arising from reporting biases). | 7 |
| Certainty assessment | 15 | Describe any methods used to assess certainty (or confidence) in the body of evidence for an outcome. | 7 |
| **RESULTS** | | |  |
| Study selection | 16a | Describe the results of the search and selection process, from the number of records identified in the search to the number of studies included in the review, ideally using a flow diagram. | 8 |
|  | 16b | Cite studies that might appear to meet the inclusion criteria, but which were excluded, and explain why they were excluded. | Figure 1 |
| Study characteristics | 17 | Cite each included study and present its characteristics. | Supplementary material 2 |
| Risk of bias in studies | 18 | Present assessments of risk of bias for each included study. | 9 |
| Results of individual studies | 19 | For all outcomes, present, for each study: (a) summary statistics for each group (where appropriate) and (b) an effect estimate and its precision (e.g. confidence/credible interval), ideally using structured tables or plots. | 8 |
| Results of syntheses | 20a | For each synthesis, briefly summarise the characteristics and risk of bias among contributing studies. | 8 |
|  | 20b | Present results of all statistical syntheses conducted. If meta-analysis was done, present for each the summary estimate and its precision (e.g. confidence/credible interval) and measures of statistical heterogeneity. If comparing groups, describe the direction of the effect. | 8 |
|  | 20c | Present results of all investigations of possible causes of heterogeneity among study results. | 8 |
|  | 20d | Present results of all sensitivity analyses conducted to assess the robustness of the synthesized results. | 8-9 |
| Reporting biases | 21 | Present assessments of risk of bias due to missing results (arising from reporting biases) for each synthesis assessed. | 9 |
| Certainty of evidence | 22 | Present assessments of certainty (or confidence) in the body of evidence for each outcome assessed. | 9 |
| **DISCUSSION** | | |  |
| Discussion | 23a | Provide a general interpretation of the results in the context of other evidence. | 9-12 |
|  | 23b | Discuss any limitations of the evidence included in the review. | 12 |
|  | 23c | Discuss any limitations of the review processes used. | 12 |
|  | 23d | Discuss implications of the results for practice, policy, and future research. | 12-13 |
| **OTHER INFORMATION** | | |  |
| Registration and protocol | 24a | Provide registration information for the review, including register name and registration number, or state that the review was not registered. | 5 |
|  | 24b | Indicate where the review protocol can be accessed, or state that a protocol was not prepared. | 5 |
|  | 24c | Describe and explain any amendments to information provided at registration or in the protocol. | 12 |
| Support | 25 | Describe sources of financial or non-financial support for the review, and the role of the funders or sponsors in the review. | 14 |
| Competing interests | 26 | Declare any competing interests of review authors. | 14 |
| Availability of data, code and other materials | 27 | Report which of the following are publicly available and where they can be found: template data collection forms; data extracted from included studies; data used for all analyses; analytic code; any other materials used in the review. | 13 |

# Table S2. Timing of mortality and risk of bias assessment of the included studies in order of publication year

| **Author** | **Year** | **Timing of mortality at the longest follow-up available** | D1 | D2 | D3 | D4 | D5 | Overall |
| --- | --- | --- | --- | --- | --- | --- | --- | --- |
| Zuurmond WW | 1987 | Hospital | Some concerns | High risk | Low | Low | Low | High risk |
| AitKenhead AR | 1989 | 28 days | Some concerns | Low | Some concerns | Some concerns | Low | Some concerns |
| Harris CE | 1990 | ICU | High risk | Some concerns | High risk | High risk | Some concerns | High risk |
| Van Hemelrijck J | 1991 | Periprocedural | Some concerns | High risk | Some concerns | Some concerns | Low | High risk |
| Degauque C | 1991 | Not specified | Low | Low | Low | Low | Low | Low |
| Lebovic S | 1992 | Hospital | Some concerns | High risk | Low | Some concerns | High risk | High risk |
| Oddby-Muhrbeck E | 1993 | Not specified | Some concerns | Some concerns | Low | Some concerns | Some concerns | Some concerns |
| Todd MM | 1993 | Hospital | Some concerns | Low | Low | Some concerns | Low | Some concerns |
| Jakobsson J | 1993 | Unclear | Some concerns | High risk | Low | Low | Low | High risk |
| Hannallah RS | 1994 | Hospital | High risk | High risk | Some concerns | High risk | Some concerns | High risk |
| Oddby-Muhrbeck E | 1994 | Not specified | Low | Low | Low | Low | Low | Low |
| Jakobsson J | 1995 | Not specified | Some concerns | Some concerns | Some concerns | Some concerns | Low | High risk |
| Kress JP | 1996 | ICU | Some concerns | Some concerns | Some concerns | Low | Low | Some concerns |
| Cheng DCH | 1996 | Hospital | Low | Some concerns | Some concerns | Some concerns | Low | Some concerns |
| Wahr JA | 1996 | Not specified | Some concerns | Low | Some concerns | Low | Low | Some concerns |
| Chamorro C | 1996 | Hospital | Low | Low | Low | Some concerns | Low | Some concerns |
| Juvin P | 1997 | Not specified | Some concerns | Some concerns | Some concerns | Low | Low | Some concerns |
| Nelskyla K | 1997 | ICU | Some concerns | High risk | High risk | Some concerns | Some concerns | High risk |
| Barrientos-Vega R | 1997 | Not specified | Some concerns | Some concerns | Low | Some concerns | Low | Some concerns |
| Manley NM | 1997 | Hospital | Low | Some concerns | Some concerns | Some concerns | Low | Some concerns |
| Jakobsson J | 1997 | Not specified | Some concerns | Some concerns | Some concerns | Some concerns | Low | High risk |
| Carrasco G | 1998 | ICU | Low | Low | Some concerns | Low | Low | Some concerns |
| Guard BC | 1998 | Hospital | Low | Low | Some concerns | High risk | High risk | High risk |
| Koscielniak-Nielsen ZJ | 1998 | Not specified | Some concerns | Low | Some concerns | Low | Low | Some concerns |
| Sanchez-Izquierdo-Riera JA | 1998 | Hospital | Low | Low | Low | Some concerns | Low | Some concerns |
| Godet G | 1998 | Periprocedural | Low | Some concerns | Some concerns | Some concerns | Low | Some concerns |
| Engoren MC | 1998 | Hospital | Low | Low | Low | Some concerns | Low | Some concerns |
| McCollam JS | 1999 | 10 days | Some concerns | Low | Low | Some concerns | Low | Some concerns |
| Kelly DF | 1999 | 6 months | Some concerns | Some concerns | Some concerns | Low | Low | Some concerns |
| Serin S | 1999 | Not specified | Some concerns | High risk | Some concerns | Low | Low | High risk |
| Jellish WS | 1999 | Hospital | Some concerns | High risk | Some concerns | Low | Low | High risk |
| Gravel NR | 1999 | Hospital | Low | Low | Low | Some concerns | Low | Some concerns |
| Juvin P | 2000 | Not specified | Some concerns | Some concerns | Low | Low | Low | Some concerns |
| Chung F | 2000 | Not specified | Low | Some concerns | Low | Some concerns | Low | Some concerns |
| Picard V | 2000 | Periprocedural | Low | Low | Low | Some concerns | High risk | High risk |
| Zhou TJ | 2000 | Hospital | Some concerns | Low | Some concerns | High risk | Low | High risk |
| Higgins TL | 2000 | 28 days | Some concerns | Low | Low | High risk | Some concerns | High risk |
| AstraZeneca | 2001 | 28 days | Some concerns | Some concerns | Low | Low | Low | Some concerns |
| Venn RM | 2001 | ICU | Low | Low | Some concerns | Some concerns | Low | Some concerns |
| Story DA | 2001 | 72 hours | Some concerns | Low | Low | Low | Low | Some concerns |
| Hall RI | 2001 | ICU | Low | Low | High risk | High risk | Some concerns | High risk |
| Godet G | 2001 | Periprocedural | Low | Some concerns | High risk | High risk | Low | High risk |
| Kress JP | 2001 | ICU | Low | Low | Low | Low | Low | Low |
| Munoz HR | 2002 | Not specified | Some concerns | Low | Some concerns | Some concerns | Low | Some concerns |
| De Hert SG | 2002 | Not specified | Some concerns | Some concerns | Low | Some concerns | Low | Some concerns |
| Song D | 2002 | Not specified | Low | Low | Low | Low | Low | Low |
| Dolk A | 2002 | Not specified | Some concerns | Low | Low | High risk | Low | High risk |
| Albera R | 2003 | Not specified | Some concerns | Some concerns | Some concerns | Some concerns | Some concerns | High risk |
| Conzen PF | 2003 | Hospital | Some concerns | Low | Low | Low | Low | Some concerns |
| De Hert SG | 2003 | Hospital | Some concerns | Some concerns | Low | Low | Low | Some concerns |
| Jellish WS | 2003 | Not specified | Some concerns | Low | Some concerns | Low | Low | Some concerns |
| Elliott RA | 2003 | Not specified | Low | Low | Low | Some concerns | Low | Some concerns |
| Parker FC | 2004 | ICU | Low | Some concerns | Some concerns | Low | Low | Some concerns |
| Chen Z | 2004 | Periprocedural | Low | Low | Low | Low | Low | Low |
| Kendal JB | 2004 | Hospital | Low | Some concerns | Low | Low | Low | Some concerns |
| De Hert SG | 2004 | Hospital | Low | Some concerns | Low | Low | Low | Some concerns |
| Cohen IT | 2004 | Periprocedural | Some concerns | Low | Some concerns | High risk | Low | High risk |
| Arar C | 2005 | Not specified | Some concerns | Some concerns | Low | Some concerns | Low | Some concerns |
| Bein B | 2005 | Not specified | Low | Some concerns | Low | Low | Low | Some concerns |
| Corbett SM | 2005 | ICU | Low | Some concerns | Low | Some concerns | Low | Some concerns |
| Malagon I | 2005 | 30 days | Low | Low | Low | Low | Low | Low |
| Prins SA | 2005 | Periprocedural | Some concerns | Low | Low | Low | Low | Some concerns |
| Graziola E | 2005 | Hospital | Some concerns | Some concerns | High risk | High risk | Low | High risk |
| Kostopanagiotou G | 2006 | Not specified | Low | Low | Some concerns | Low | Low | Some concerns |
| Law-Koune JD | 2006 | Hospital | Some concerns | Some concerns | Some concerns | Some concerns | Low | Some concerns |
| Lo YL | 2006 | Not specified | Some concerns | High risk | Low | Low | Some concerns | High risk |
| Lorsomradee S | 2006 | Hospital | Low | Some concerns | Some concerns | Low | Low | Some concerns |
| Carson SS | 2006 | Hospital | Low | Low | Low | Low | Low | Low |
| Cromheeke S | 2006 | Not specified | Low | Some concerns | Some concerns | Low | Low | Some concerns |
| Shirakami G | 2006 | Hospital | Low | High risk | Some concerns | Low | Low | High risk |
| [Hanss R](http://www.ncbi.nlm.nih.gov/pubmed?term=Hanss%20R%5BAuthor%5D&cauthor=true&cauthor_uid=16500952) | 2006 | 6 months | Some concerns | Low | Some concerns | Low | Low | Some concerns |
| Guarracino F | 2006 | 30 days | Low | Low | Low | Low | Low | Low |
| Wallden | 2006 | Not specified | Low | Low | Some concerns | Low | Low | Low |
| Collini S | 2006 | Periprocedural | Low | Low | Low | Low | Low | Low |
| Rabie M | 2006 | Not specified | Some concerns | Some concerns | Some concerns | High risk | Low | High risk |
| Musialowicz T | 2007 | Not specified | Some concerns | Some concerns | Some concerns | Low | Low | Some concerns |
| Vanacker BF | 2007 | Periprocedural | High risk | Low | Some concerns | Low | High risk | High risk |
| Tritapepe L | 2007 | 30 days | Low | Low | Low | Low | Low | Low |
| Ghori KA | 2007 | 3 months | Low | Some concerns | Some concerns | Low | Low | Some concerns |
| Bhagat H | 2008 | Not specified | Some concerns | Some concerns | Some concerns | Low | Low | Some concerns |
| Cavalca V | 2008 | Periprocedural | Low | Low | Low | Low | Low | Low |
| Cheng SS | 2008 | Periprocedural | Some concerns | Low | Some concerns | Low | Low | Some concerns |
| Ko JS | 2008 | 30 days | Low | Some concerns | Low | Low | Low | Some concerns |
| Okuyucu S | 2008 | Periprocedural | Low | Low | Low | Low | Low | Low |
| Huey-Ling L | 2008 | ICU | Some concerns | High risk | Some concerns | High risk | Low | High risk |
| Maldonado JR | 2009 | Hospital | Some concerns | Some concerns | Some concerns | Some concerns | Low | Some concerns |
| Memis D. | 2009 | ICU | Low | Low | Low | Some concerns | Low | Some concerns |
| Kazanci D | 2009 | Periprocedural | Some concerns | Some concerns | Low | Low | Low | Some concerns |
| Khurana P | 2009 | Not specified | Some concerns | Low | Some concerns | Some concerns | Low | Some concerns |
| Hasani A | 2009 | Hospital | Low | Low | Low | Low | Low | Low |
| Tasdogan M | 2009 | ICU | Low | Low | Low | Low | Low | Low |
| Yildirim V | 2009 | 30 days | Low | Some concerns | Low | Some concerns | Low | Some concerns |
| Ionescu D | 2009 | Not specified | Low | Low | Low | Low | Low | Low |
| Van Der Linden P | 2010 | Hospital | Low | Some concerns | Some concerns | Low | Low | Some concerns |
| Strom T | 2010 | Hospital | Low | Low | Low | Some concerns | Low | Some concerns |
| Song JC | 2010 | Hospital | Low | Some concerns | Low | Low | Low | Some concerns |
| Flier S* | 2010 | 1 year | Low | Low | Low | Low | Low | Low |
| Ballester M | 2011 | 49 days | Low | Some concerns | Low | Low | Low | Some concerns |
| Bignami E | 2011 | 1 year | Low | Low | Low | Low | Low | Low |
| Uri O | 2011 | Not specified | Some concerns | High risk | Low | High risk | Low | High risk |
| Zoremba M | 2011 | Not specified | Some concerns | Low | Low | Low | Low | Some concerns |
| Tempe DK | 2011 | 7 days | High risk | High risk | Some concerns | Some concerns | Low | High risk |
| Schoen J | 2011 | Hospital | Low | Low | Low | Low | Low | Low |
| Schilling T | 2011 | Hospital | Low | Low | Low | Low | Low | Low |
| Royse CF | 2011 | Not specified | Low | Low | Low | Low | Low | Low |
| Rossetti AO | 2011 | 3 months | Low | High risk | High risk | Low | Low | Low |
| Zangrillo A* | 2011 | 1 year | Low | Low | Low | Low | Low | Low |
| Huang Z | 2011 | Hospital | Low | High risk | Low | Low | Low | High risk |
| Kottemberg E | 2012 | Hospital | Low | Low | Low | Low | Low | Low |
| Laviolle B | 2012 | Not specified | High risk | Some concerns | Low | Low | Low | High risk |
| Lurati Buse GAL | 2012 | 1 year | Low | Low | Low | Low | Low | Low |
| Moller Petrun A | 2012 | Not specified | Some concerns | Low | Low | Low | Low | Some concerns |
| Nagao Y | 2012 | Not specified | Low | Low | Low | Low | Low | Low |
| Bjelland TW | 2012 | Not specified | Low | Some concerns | Some concerns | Some concerns | Low | Some concerns |
| Chi X | 2012 | Not specified | Some concerns | Some concerns | Low | High risk | Low | High risk |
| Konstantopoulos K | 2012 | Not specified | Low | Some concerns | Some concerns | Low | Low | Some concerns |
| Bindra A | 2012 | Periprocedural | Low | Low | Low | Low | Low | Low |
| Terao Y | 2012 | Hospital | Low | Low | Some concerns | High risk | Low | High risk |
| Tanguy | 2012 | Hospital | Some concerns | Low | Low | Low | Low | Some concerns |
| Soro M | 2012 | Hospital | Low | Low | Low | Low | Low | Low |
| Song JG | 2012 | Not specified | Low | High risk | Some concerns | Low | Low | High risk |
| Jakob SM PRODEX | 2012 | 6 months | Low | Low | Low | Some concerns | Low | Some concerns |
| Wu J | 2012 | Not specified | Low | Low | Low | Low | Some concerns | Some concerns |
| De la Mora-González JF | 2012 | Not specified | Some concerns | Some concerns | Some concerns | High risk | Low | High risk |
| Jovic M | 2012 | Hospital | Some concerns | Some concerns | Low | Some concerns | Low | Some concerns |
| Xu WY | 2013 | Hospital | Low | Some concerns | Low | Low | Some concerns | Some concerns |
| Braz MG | 2013 | Not specified | Some concerns | Some concerns | Some concerns | High risk | Low | High risk |
| Kowalczyk M | 2013 | Not specified | Some concerns | Some concerns | Some concerns | Some concerns | Low | Some concerns |
| Mazoti MA | 2013 | Hospital | Low | Some concerns | Some concerns | Low | Low | Some concerns |
| Mencke T | 2013 | Not specified | Low | Low | Low | Low | Low | Low |
| Kalimeris K | 2013 | Not specified | Low | Some concerns | Some concerns | Some concerns | Low | Some concerns |
| Ozturk I | 2013 | Periprocedural | Some concerns | Some concerns | Some concerns | Some concerns | High risk | High risk |
| Baki ED | 2013 | Not specified | Some concerns | Some concerns | Low | Some concerns | Low | Some concerns |
| Chaaban MR | 2013 | Not specified | Low | Some concerns | Some concerns | Some concerns | Low | Some concerns |
| Dahaba AA | 2013 | Not specified | Low | Low | Low | Low | Low | Low |
| Lindholm EE* | 2013 | 3 years | Low | Low | Low | Low | Low | Low |
| Sofra M | 2013 | Not specified | Some concerns | Low | Low | Low | Low | Some concerns |
| Song J-C | 2013 | Hospital | Low | High risk | Some concerns | Some concerns | Low | High risk |
| Ziemann-Gimmel P | 2013 | Not specified | Low | Low | Low | Low | Low | Low |
| Valencia L | 2013 | Not specified | Low | Some concerns | Some concerns | Low | Low | Some concerns |
| Grendelmeier P | 2014 | 28 days | Low | Low | Low | Low | Low | Low |
| Zhou Y | 2014 | Hospital | Low | Low | Low | Low | Low | Low |
| Shah PN | 2014 | ICU | Low | Low | Low | Low | Some concerns | Some concerns |
| Mroziński P | 2014 | Not specified | High risk | Low | Low | High risk | Some concerns | High risk |
| Erturk E | 2014 | Periprocedural | Low | Some concerns | Low | Low | Some concerns | Some concerns |
| Chen J | 2014 | Periprocedural | Low | Some concerns | Low | Low | Some concerns | Some concerns |
| Yoo YC | 2014 | Hospital | Low | Low | Low | Low | Low | Low |
| Rozec B | 2014 | Hospital | Low | Low | Low | Low | Low | Low |
| Liang C | 2014 | Periprocedural | Some concerns | Some concerns | High risk | Low | Low | High risk |
| Margarit SC | 2014 | Periprocedural | Low | Low | Some concerns | Some concerns | Low | Some concerns |
| Ortiz J | 2014 | Not specified | Low | Some concerns | Low | Low | Low | Some concerns |
| Parida S | 2014 | Not specified | Low | High risk | Low | Low | Some concerns | High risk |
| Yu JB | 2014 | Not specified | Low | Some concerns | Some concerns | Some concerns | Low | Some concerns |
| Jia L | 2015 | Hospital | Low | Low | Low | Low | Low | Low |
| Bastola P | 2015 | Periprocedural | Low | Low | Low | Low | Low | Low |
| Goswami U | 2015 | Not specified | Low | Low | Low | Low | Low | Low |
| Kim YS | 2015 | 6 months | Low | Low | Low | Low | Low | Low |
| Lehavi A | 2015 | Periprocedural | Low | Some concerns | Low | Low | Some concerns | Some concerns |
| Karaman Y | 2015 | ICU | Low | Low | Low | Low | Low | Low |
| Dabir S | 2015 | Periprocedural | Low | Low | Low | Low | Low | Low |
| Liu S | 2016 | Hospital | Low | Low | Low | Low | Low | Low |
| Moro ET | 2016 | Hospital | Low | Low | Low | Low | Low | Low |
| Liu X | 2016 | Hospital | Low | Low | Low | Low | Low | Low |
| Doe A | 2016 | Hospital | Low | Low | Low | Low | Low | Low |
| Likhvantsev VV* | 2016 | 1 year | Low | Low | Low | Low | Low | Low |
| Biedrzycka A | 2016 | Not specified | Some concerns | Low | Low | Some concerns | Low | Some concerns |
| Ammar AS | 2016 | 30 days | Low | Low | Low | Low | Low | Low |
| Conti G | 2016 | ICU | Low | Low | Low | Low | Low | Low |
| Khalil M | 2016 | Hospital | Low | Some concerns | Low | Low | Some concerns | Some concerns |
| Shah PJ | 2016 | Periprocedural | Low | Some concerns | Low | Low | Some concerns | Some concerns |
| Beck-Schimmer B | 2016 | Not specified | Low | Low | Low | Low | Low | Low |
| Goettel N | 2016 | Hospital | Low | Low | Low | Low | Low | Low |
| Markovic-Bozic J | 2016 | 15 days | Low | Low | Low | Low | Low | Low |
| Erdem AF | 2016 | Periprocedural | Low | Some concerns | Low | Low | Some concerns | Some concerns |
| Djaiani G | 2016 | Periprocedural | Low | Low | Low | Low | Low | Low |
| Bhakta P | 2016 | Not specified | Low | Some concerns | Some concerns | Low | Low | Some concerns |
| Hassan WMNW | 2017 | Hospital | Low | Low | Low | Low | Low | Low |
| de la Gala F* | 2017 | 1 year | Low | Low | Low | Low | Low | Low |
| Hofland J | 2017 | Hospital | Low | Low | Low | Low | Low | Low |
| Zhang Y | 2017 | 3 months | Low | Low | Low | Low | Low | Low |
| Guerrero Orriach JL | 2017 | ICU | Low | Low | Low | Low | Low | Low |
| Tanaka P | 2017 | 2 days | Low | Low | Low | Low | Low | Low |
| Yang XL | 2017 | Hospital | Low | Low | Low | Low | Low | Low |
| Cho YJ | 2017 | Periprocedural | Low | Low | Low | Low | Low | Low |
| Nieuwenhuijs-Moeke GJ | 2017 | Not specified | Low | Low | Low | Low | Low | Low |
| Little M | 2018 | Periprocedural | Low | Low | Low | Low | Low | Low |
| Zhang Y | 2018 | 3 months | Low | Low | Low | Low | Low | Low |
| Ji FH | 2018 | 30 days | Low | Low | Low | Low | Low | Low |
| Oh CS | 2018 | Hospital | Low | Low | Low | Low | Low | Low |
| Sheikh TA | 2018 | Hospital | Low | Low | Low | Low | Low | Low |
| Wojarska-Tręda E | 2018 | Periprocedural | Some concerns | Some concerns | Low | Low | Some concerns | Some concerns |
| Wąsowicz M | 2018 | Hospital | Low | Low | Low | Low | Low | Low |
| Mei B | 2018 | 30 days | Low | Low | Low | Low | Low | Low |
| Moscarelli M | 2018 | Hospital | Low | Low | Low | Low | Low | Low |
| Kuzkov VV | 2018 | Hospital | Low | Some concerns | Low | Low | Some concerns | Some concerns |
| Eshghpour M | 2018 | Periprocedural | Low | Low | Low | Low | Low | Low |
| Sigler MB | 2018 | 28 days | Low | Some concerns | Low | Some concerns | Low | Some concerns |
| Sahoo AK | 2019 | Not specified | Low | Low | Low | Some concerns | Low | Some concerns |
| Aditianingsih D | 2019 | 1 year | Some concerns | Low | Low | Some concerns | Low | Some concerns |
| Wu ZF | 2019 | 3 years | Low | Some concerns | Low | Low | Some concerns | Some concerns |
| Hahm TS | 2019 | Periprocedural | Low | Low | Low | Low | Low | Low |
| Kim D | 2019 | Periprocedural | Low | Low | Low | Low | Low | Low |
| Wu B | 2019 | 3 months | Low | Low | Low | Low | Low | Low |
| Roh GU | 2019 | Hospital | Some concerns | Low | Low | Low | Low | Some concerns |
| Velayutham P | 2019 | Periprocedural | Low | Low | Low | Low | Low | Low |
| Shi Y | 2019 | Hospital | Low | Some concerns | Low | Low | Some concerns | Some concerns |
| Wang W | 2019 | 28 days | Low | Some concerns | Low | Low | Some concerns | Some concerns |
| Lin WL | 2019 | 72 hours | Low | Low | Low | Low | Some concerns | Some concerns |
| Jo JY | 2019 | Periprocedural | Low | Low | Low | Low | Low | Low |
| Guinot PG | 2020 | 30 days | Low | Some concerns | Low | Low | Low | Some concerns |
| Park J | 2020 | Hospital | Low | Some concerns | Low | Low | Low | Some concerns |
| Haldar R | 2020 | Hospital | Low | Low | Low | Low | Low | Low |
| Pandit A | 2020 | Not specified | Some concerns | Low | Low | Low | Low | Some concerns |
| Mei X | 2020 | Periprocedural | Low | Low | Low | Low | Low | Low |
| Hovaguimian F | 2020 | Not specified | Low | Low | Low | Low | Low | Low |
| Shin S* | 2020 | 90 days | Low | Low | Low | Low | Low | Low |
| Doi M | 2020 | Periprocedural | Some concerns | Low | Low | Some concerns | Low | Some concerns |
| Li X | 2020 | Periprocedural | Low | Low | Low | Some concerns | Low | Some concerns |
| Efremov SM | 2020 | Not specified | Low | Low | Low | Low | Low | Low |
| Guo L* | 2020 | 3 months | Low | Low | Low | Low | Low | Low |
| Mei B | 2020 | Not specified | Some concerns | Low | Low | Low | Low | Some concerns |
| Weng Y | 2020 | 30 days | Some concerns | Some concerns | Low | Low | Low | Some concerns |
| Gollapudy S | 2020 | Not specified | Low | Low | Low | Low | Low | Low |
| Liu J | 2020 | 28 days | Some concerns | Some concerns | Some concerns | Some concerns | Low | High risk |
| Chitnis S | 2021 | Hospital | Low | Low | Low | Some concerns | Low | Some concerns |
| Niu Z | 2021 | Hospital | Low | Low | Low | Low | Low | Low |
| Joe YE | 2021 | 7 days | Low | Low | Low | Low | Low | Low |
| Sato S | 2021 | Not specified | Low | Low | Low | Some concerns | Low | Some concerns |
| Siripoonyothai S | 2021 | 1 day | Low | Low | Low | Some concerns | Low | Some concerns |
| Kim SH | 2021 | Hospital | Low | Low | Low | Low | Low | Low |
| Li Y | 2021 | Hospital | Low | Low | Low | Low | Low | Low |
| Li XF | 2021 | 30 days | Low | Low | Low | Low | Low | Low |
| Fang FQ | 2021 | 30 days | Low | Some concerns | Low | Low | Low | Some concerns |
| Yu H | 2021 | 1 year | Low | Low | Low | Low | Low | Low |
| Jo JY | 2021 | Periprocedural | Low | Low | Low | Low | Low | Low |
| Winings NA | 2021 | ICU | Some concerns | Low | Low | Low | Low | Some concerns |
| Hughes CG | 2021 | 28 days | Low | Low | Low | Low | Low | Low |
| Kim NY | 2021 | Not specified | Some concerns | Low | Low | Some concerns | Low | Some concerns |
| Liu Y | 2021 | 28 days | Low | Some concerns | Low | Low | Some concerns | Some concerns |
| Dubowitz JA | 2021 | Not specified | Low | Low | Low | Low | Low | Low |
| Kawagoe I | 2022 | Periprocedural | Low | Low | Low | Some concerns | Low | Some concerns |
| Mao Y | 2022 | Periprocedural | Low | Low | Low | Low | Some concerns | Some concerns |
| Zhou Y | 2022 | Hospital | Low | Low | Low | Some concerns | Some concerns | Some concerns |
| Kawanishi R | 2022 | 5 years | Low | Low | Some concerns | Low | Some concerns | Some concerns |
| Zhang J | 2022 | Periprocedural | Some concerns | Low | Low | Low | Low | Some concerns |
| Kim JE | 2022 | Periprocedural | Low | Low | Low | Some concerns | Low | Some concerns |
| Choi EK | 2022 | Periprocedural | Low | Low | Low | Low | Low | Low |
| Kim SH | 2022 | 6 months | Low | Low | Low | Low | Low | Low |
| Franzén S | 2022 | Not specified | Low | Low | Low | Low | Low | Low |
| Wang J | 2022 | 7 days | Low | Low | Low | Low | Low | Low |
| Liu Y | 2022 | Periprocedural | Low | Low | Low | Some concerns | Low | Some concerns |
| Lu Z | 2022 | 1 year | Low | Low | Low | Low | Low | Low |

Abbreviations: ICU = intensive care unit

* indicates studies reporting mortality at 30-day and at a longer timepoint.

# Table S3. Timepoints of mortality as reported in the 252 studies

| **Timing of mortality at the longest follow-up available** | **Number of studies (%)** |
| --- | --- |
| Hospital | 69 (27) |
| Periprocedural | 43 (17) |
| Intensive care unit | 22 (8.7) |
| 30 days | 20 (7.9) |
| 1 year | 10 (4.0) |
| 28 days | 9 (3.6) |
| 6 months | 8 (3.2) |
| 3 months | 6 (2.4) |
| 7 days | 4 (1.6) |
| 3 years | 2 (0.8) |
| 72 hours | 2 (0.8) |
| 5 years | 1 (0.4) |
| 49 days | 1 (0.4) |
| 15 days | 1 (0.4) |
| 10 days | 1 (0.4) |
| 2 days | 1 (0.4) |
| 1 day | 1 (0.4) |
| Not specified | 71 (28) |

# Table S4: Mortality at different timepoints reported in the included studies

| Timepoint of mortality | No. of studies | Propofol | Control | Risk ratio  (95% CI) | P value | I^2^ |
| --- | --- | --- | --- | --- | --- | --- |
| Hospital | 69 | 130/3,112 (4.2%) | 112/3,679 (3.0%) | 1.15 (0.93–1.41) | 0.21 | 0% |
| Periprocedural | 43 | 0/1,574 (0%) | 2/1,900 (0.1%) | 0.33 (0.04–3.14) | 0.34 | 0% |
| Intensive care unit | 22 | 132/960 (14%) | 107/1,043 (10%) | 1.23 (0.99–1.53) | 0.07 | 0% |
| 30 days | 20 | 32/1,836 (1.7%) | 31/2,078 (1.5%) | 1.05 (0.66–1.67) | 0.83 | 0% |
| 1 year | 10 | 157/2,111 (7.4%) | 113/2,121 (5.3%) | 1.37 (1.09–1.73) | 0.007 | 21% |

We considered mortality data at all timepoints reported in the eligible studies.
