# Supplementary material for: Propofol and survival: an updated meta-analysis of randomized clinical trials
Source: Crit Care. 2023 Apr 12;27:139. doi: 10.1186/s13054-023-04431-8 (PMC10099692; doi:10.1186/s13054-023-04431-8)
Supplement: Supplementary file 2 — Additional file 2. Complete reference list of included studies. [file 13054_2023_4431_MOESM2_ESM.docx]

**Additional file 2: complete reference list of included studies**

**Propofol and survival. An updated meta-analysis of randomized clinical trials.**

**Authors**

Yuki Kotani, MD, Alessandro Pruna, MD, Stefano Turi, MD, Giovanni Borghi, MD, Todd C Lee, MPH, Alberto Zangrillo, MD, Giovanni Landoni, MD, Laura Pasin, MD

# References of 252 included studies

* indicates that the author provided additional data on the study.

1. Zuurmond WW, van Leeuwen L, Helmers JH. Recovery from propofol infusion as the main agent for outpatient arthroscopy. A comparison with isoflurane. Anaesthesia 1987;42(4):356-9. (In eng). DOI: 10.1111/j.1365-2044.1987.tb03974.x.

2. Aitkenhead AR, Pepperman ML, Willatts SM, et al. Comparison of propofol and midazolam for sedation in critically ill patients. Lancet 1989;2(8665):704-9. (In eng). DOI: 10.1016/s0140-6736(89)90770-8.

3. Harris CE, Grounds RM, Murray AM, Lumley J, Royston D, Morgan M. Propofol for long-term sedation in the intensive care unit. A comparison with papaveretum and midazolam. Anaesthesia 1990;45(5):366-72. (In eng). DOI: 10.1111/j.1365-2044.1990.tb14777.x.

4. A study to compare the use of propofol and midazolam for the sedatory of patients with acute respiratory failure. Journal of Drug Development 1992;4(Suppl 3):95-97. (https://eurekamag.com/research/029/810/029810070.php).

5. Van Hemelrijck J, Van Aken H, Merckx L, Mulier J. Anesthesia for craniotomy: total intravenous anesthesia with propofol and alfentanil compared to anesthesia with thiopental sodium, isoflurane, fentanyl, and nitrous oxide. J Clin Anesth 1991;3(2):131-6. (In eng). DOI: 10.1016/0952-8180(91)90010-k.

6. Lebovic S, Reich DL, Steinberg LG, Vela FP, Silvay G. Comparison of propofol versus ketamine for anesthesia in pediatric patients undergoing cardiac catheterization. Anesth Analg 1992;74(4):490-4. (In eng). DOI: 10.1213/00000539-199204000-00003.

7*. Jakobsson J, Oddby E, Rane K. Patient evaluation of four different combinations of intravenous anaesthetics for short outpatient procedures. Anaesthesia 1993;48(11):1005-7. (In eng). DOI: 10.1111/j.1365-2044.1993.tb07485.x.

8*. Oddby-Muhrbeck E, Jakobsson J. Recall of music: a comparison between anaesthesia with propofol and isoflurane. Acta Anaesthesiol Scand 1993;37(1):33-7. (In eng). DOI: 10.1111/j.1399-6576.1993.tb03594.x.

9. Todd MM, Warner DS, Sokoll MD, et al. A prospective, comparative trial of three anesthetics for elective supratentorial craniotomy. Propofol/fentanyl, isoflurane/nitrous oxide, and fentanyl/nitrous oxide. Anesthesiology 1993;78(6):1005-20. (In eng). DOI: 10.1097/00000542-199306000-00002.

10. Hannallah RS, Britton JT, Schafer PG, Patel RI, Norden JM. Propofol anaesthesia in paediatric ambulatory patients: a comparison with thiopentone and halothane. Can J Anaesth 1994;41(1):12-8. (In eng). DOI: 10.1007/bf03009654.

11*. Oddby-Muhrbeck E, Jakobsson J, Andersson L, Askergren J. Postoperative nausea and vomiting. A comparison between intravenous and inhalation anaesthesia in breast surgery. Acta Anaesthesiol Scand 1994;38(1):52-6. (In eng). DOI: 10.1111/j.1399-6576.1994.tb03837.x.

12*. Jakobsson J, Rane K. Anaesthesia for short outpatient procedures. A comparison between thiopentone and propofol in combination with fentanyl or alfentanil. Acta Anaesthesiol Scand 1995;39(4):503-7. (In eng). DOI: 10.1111/j.1399-6576.1995.tb04108.x.

13. Chamorro C, de Latorre FJ, Montero A, et al. Comparative study of propofol versus midazolam in the sedation of critically ill patients: results of a prospective, randomized, multicenter trial. Crit Care Med 1996;24(6):932-9. (In eng). DOI: 10.1097/00003246-199606000-00010.

14. Cheng DC, Karski J, Peniston C, et al. Morbidity outcome in early versus conventional tracheal extubation after coronary artery bypass grafting: a prospective randomized controlled trial. J Thorac Cardiovasc Surg 1996;112(3):755-64. (In eng). DOI: 10.1016/s0022-5223(96)70062-4.

15. Kress JP, O'Connor MF, Pohlman AS, et al. Sedation of critically ill patients during mechanical ventilation. A comparison of propofol and midazolam. Am J Respir Crit Care Med 1996;153(3):1012-8. (In eng). DOI: 10.1164/ajrccm.153.3.8630539.

16. Wahr JA, Plunkett JJ, Ramsay JG, et al. Cardiovascular responses during sedation after coronary revascularization. Incidence of myocardial ischemia and hemodynamic episodes with propofol versus midazolam. Institutions of the McSPI Research Group. Anesthesiology 1996;84(6):1350-60. (In eng). DOI: 10.1097/00000542-199606000-00011.

17. Barrientos-Vega R, Mar Sánchez-Soria M, Morales-García C, Robas-Gómez A, Cuena-Boy R, Ayensa-Rincon A. Prolonged sedation of critically ill patients with midazolam or propofol: impact on weaning and costs. Crit Care Med 1997;25(1):33-40. (In eng). DOI: 10.1097/00003246-199701000-00009.

18*. Jakobsson J, Hellquist E, Bastos M. Cost-effective anaesthesia for outpatient arthroscopic surgery: Desflurane versus propofol? Ambulatory Surgery 1997;5(2):67-70. DOI: https://doi.org/10.1016/S0966-6532(97)00036-X.

19*. Juvin P, Servin F, Giraud O, Desmonts JM. Emergence of elderly patients from prolonged desflurane, isoflurane, or propofol anesthesia. Anesth Analg 1997;85(3):647-51. (In eng). DOI: 10.1097/00000539-199709000-00029.

20. Manley NM, Fitzpatrick RW, Long T, Jones PW. A cost analysis of alfentanil+propofol vs morphine+midazolam for the sedation of critically ill patients. Pharmacoeconomics 1997;12(2 Pt 2):247-55. (In eng). DOI: 10.2165/00019053-199712020-00015.

21. Nelskylä K, Eriksson H, Soikkeli A, Korttila K. Recovery and outcome after propofol and isoflurane anesthesia in patients undergoing laparoscopic hysterectomy. Acta Anaesthesiol Scand 1997;41(3):360-3. (In eng). DOI: 10.1111/j.1399-6576.1997.tb04699.x.

22. Carrasco G, Cabré L, Sobrepere G, et al. Synergistic sedation with propofol and midazolam in intensive care patients after coronary artery bypass grafting. Crit Care Med 1998;26(5):844-51. (In eng). DOI: 10.1097/00003246-199805000-00015.

23. Engoren MC, Kraras C, Garzia F. Propofol-based versus fentanyl-isoflurane-based anesthesia for cardiac surgery. J Cardiothorac Vasc Anesth 1998;12(2):177-81. (In eng). DOI: 10.1016/s1053-0770(98)90328-7.

24. Godet G, Gossens S, Prayssac P, et al. Infusion of propofol, sufentanil, or midazolam for sedation after aortic surgery: comparison of oxygen consumption and hemodynamic stability. Anesth Analg 1998;87(2):272-6. (In eng). DOI: 10.1097/00000539-199808000-00007.

25. Guard BC, Sikich N, Lerman J, Levine M. Maintenance and recovery characteristics after sevoflurane or propofol during ambulatory surgery in children with epidural blockade. Can J Anaesth 1998;45(11):1072-8. (In eng). DOI: 10.1007/bf03012394.

26. Koscielniak-Nielsen ZJ, Stens-Pedersen HL, Hesselbjerg L. Midazolam-flumazenil versus propofol anaesthesia for scoliosis surgery with wake-up tests. Acta Anaesthesiol Scand 1998;42(1):111-6. (In eng). DOI: 10.1111/j.1399-6576.1998.tb05090.x.

27. Sanchez-Izquierdo-Riera JA, Caballero-Cubedo RE, Perez-Vela JL, Ambros-Checa A, Cantalapiedra-Santiago JA, Alted-Lopez E. Propofol versus midazolam: safety and efficacy for sedating the severe trauma patient. Anesth Analg 1998;86(6):1219-24. (In eng). DOI: 10.1097/00000539-199806000-00016.

28. Gravel NR, Searle NR, Taillefer J, Carrier M, Roy M, Gagnon L. Comparison of the hemodynamic effects of sevoflurane anesthesia induction and maintenance vs TIVA in CABG surgery. Can J Anaesth 1999;46(3):240-6. (In eng). DOI: 10.1007/bf03012603.

29. Jellish WS, Leonetti JP, Fahey K, Fury P. Comparison of 3 different anesthetic techniques on 24-hour recovery after otologic surgical procedures. Otolaryngol Head Neck Surg 1999;120(3):406-11. (In eng). DOI: 10.1016/s0194-5998(99)70284-6.

30. Kelly DF, Goodale DB, Williams J, et al. Propofol in the treatment of moderate and severe head injury: a randomized, prospective double-blinded pilot trial. J Neurosurg 1999;90(6):1042-52. (In eng). DOI: 10.3171/jns.1999.90.6.1042.

31. McCollam JS, O'Neil MG, Norcross ED, Byrne TK, Reeves ST. Continuous infusions of lorazepam, midazolam, and propofol for sedation of the critically ill surgery trauma patient: a prospective, randomized comparison. Crit Care Med 1999;27(11):2454-8. (In eng). DOI: 10.1097/00003246-199911000-00022.

32*. Serin S, Elibol O, Sungurtekin H, Gonullu M. Comparison of halothane/thiopental and propofol anesthesia for strabismus surgery. Ophthalmologica 1999;213(4):224-7. (In eng). DOI: 10.1159/000027426.

33*. Chung F, Mulier JP, Scholz J, et al. A comparison of anaesthesia using remifentanil combined with either isoflurane, enflurane or propofol in patients undergoing gynaecological laparoscopy, varicose vein or arthroscopic surgery. Acta Anaesthesiol Scand 2000;44(7):790-8. (In eng). DOI: 10.1034/j.1399-6576.2000.440704.x.

34. Higgins TL, Murray M, Kett DH, et al. Trace element homeostasis during continuous sedation with propofol containing EDTA versus other sedatives in critically ill patients. Intensive Care Med 2000;26 Suppl 4:S413-21. (In eng). DOI: 10.1007/pl00003785.

35*. Juvin P, Vadam C, Malek L, Dupont H, Marmuse JP, Desmonts JM. Postoperative recovery after desflurane, propofol, or isoflurane anesthesia among morbidly obese patients: a prospective, randomized study. Anesth Analg 2000;91(3):714-9. (In eng). DOI: 10.1097/00000539-200009000-00041.

36. Picard V, Dumont L, Pellegrini M. Quality of recovery in children: sevoflurane versus propofol. Acta Anaesthesiol Scand 2000;44(3):307-10. (In eng). DOI: 10.1034/j.1399-6576.2000.440315.x.

37. Zhou TJ, Tang J, White PF, et al. Reversal of rapacuronium block during propofol versus sevoflurane anesthesia. Anesth Analg 2000;90(3):689-93. (In eng). DOI: 10.1097/00000539-200003000-00033.

38. AstraZeneca LP. Dear health care provider letter. March 26, 2001.

39. Godet G, Watremez C, El Kettani C, Soriano C, Coriat P. A comparison of sevoflurane, target-controlled infusion propofol, and propofol/isoflurane anesthesia in patients undergoing carotid surgery: a quality of anesthesia and recovery profile. Anesth Analg 2001;93(3):560-5. (In eng). DOI: 10.1097/00000539-200109000-00007.

40. Hall RI, Sandham D, Cardinal P, et al. Propofol vs midazolam for ICU sedation : a Canadian multicenter randomized trial. Chest 2001;119(4):1151-9. (In eng). DOI: 10.1378/chest.119.4.1151.

41. Kress JP, Pohlman AS, Hall JB. Sedation and analgesia in the intensive care unit. Am J Respir Crit Care Med 2002;166(8):1024-8. (In eng). DOI: 10.1164/rccm.200204-270CC.

42. Story DA, Poustie S, Liu G, McNicol PL. Changes in plasma creatinine concentration after cardiac anesthesia with isoflurane, propofol, or sevoflurane: a randomized clinical trial. Anesthesiology 2001;95(4):842-8. (In eng). DOI: 10.1097/00000542-200110000-00010.

43. Venn RM, Grounds RM. Comparison between dexmedetomidine and propofol for sedation in the intensive care unit: patient and clinician perceptions. Br J Anaesth 2001;87(5):684-90. (In eng). DOI: 10.1093/bja/87.5.684.

44*. De Hert SG, ten Broecke PW, Mertens E, et al. Sevoflurane but not propofol preserves myocardial function in coronary surgery patients. Anesthesiology 2002;97(1):42-9. (In eng). DOI: 10.1097/00000542-200207000-00007.

45*. Dolk A, Cannerfelt R, Anderson RE, Jakobsson J. Inhalation anaesthesia is cost-effective for ambulatory surgery: a clinical comparison with propofol during elective knee arthroscopy. Eur J Anaesthesiol 2002;19(2):88-92. (In eng). DOI: 10.1017/s0265021502000157.

46*. Muñoz HR, Núñez GE, de la Fuente JE, Campos MG. The effect of nitrous oxide on jugular bulb oxygen saturation during remifentanil plus target-controlled infusion propofol or sevoflurane in patients with brain tumors. Anesth Analg 2002;94(2):389-92, table of contents. (In eng). DOI: 10.1097/00000539-200202000-00030.

47*. Song D, Chung F, Wong J, Yogendran S. The assessment of postural stability after ambulatory anesthesia: a comparison of desflurane with propofol. Anesth Analg 2002;94(1):60-4, table of contents. (In eng). DOI: 10.1097/00000539-200201000-00011.

48*. Albera R, Ciuffolotti R, Di Cicco M, et al. Double-blind, randomized, multicenter study comparing the effect of betahistine and flunarizine on the dizziness handicap in patients with recurrent vestibular vertigo. Acta Otolaryngol 2003;123(5):588-93. (In eng). DOI: 10.1080/00016480310001475.

49. Conzen PF, Fischer S, Detter C, Peter K. Sevoflurane provides greater protection of the myocardium than propofol in patients undergoing off-pump coronary artery bypass surgery. Anesthesiology 2003;99(4):826-33. (In eng). DOI: 10.1097/00000542-200310000-00013.

50. De Hert SG, Cromheecke S, ten Broecke PW, et al. Effects of propofol, desflurane, and sevoflurane on recovery of myocardial function after coronary surgery in elderly high-risk patients. Anesthesiology 2003;99(2):314-23. (In eng). DOI: 10.1097/00000542-200308000-00013.

51*. Elliott RA, Payne K, Moore JK, et al. Clinical and economic choices in anaesthesia for day surgery: a prospective randomised controlled trial. Anaesthesia 2003;58(5):412-21. (In eng). DOI: 10.1046/j.1365-2044.2003.03125.x.

52*. Jellish WS, Sheikh T, Baker WH, Louie EK, Slogoff S. Hemodynamic stability, myocardial ischemia, and perioperative outcome after carotid surgery with remifentanil/propofol or isoflurane/fentanyl anesthesia. J Neurosurg Anesthesiol 2003;15(3):176-84. (In eng). DOI: 10.1097/00008506-200307000-00004.

53. Chen Z. The effects of isoflurane and propofol on intraoperative neurophysiological monitoring during spinal surgery. J Clin Monit Comput 2004;18(4):303-8. (In eng). DOI: 10.1007/s10877-005-5097-5.

54. Cohen IT, Finkel JC, Hannallah RS, Goodale DB. Clinical and biochemical effects of propofol EDTA vs sevoflurane in healthy infants and young children. Paediatr Anaesth 2004;14(2):135-42. (In eng). DOI: 10.1111/j.1460-9592.2004.01160.x.

55. De Hert SG, Van der Linden PJ, Cromheecke S, et al. Cardioprotective properties of sevoflurane in patients undergoing coronary surgery with cardiopulmonary bypass are related to the modalities of its administration. Anesthesiology 2004;101(2):299-310. (In eng). DOI: 10.1097/00000542-200408000-00009.

56. Kendall JB, Russell GN, Scawn ND, Akrofi M, Cowan CM, Fox MA. A prospective, randomised, single-blind pilot study to determine the effect of anaesthetic technique on troponin T release after off-pump coronary artery surgery. Anaesthesia 2004;59(6):545-9. (In eng). DOI: 10.1111/j.1365-2044.2004.03713.x.

57. Parker FC, Story DA, Poustie S, Liu G, McNicol L. Time to tracheal extubation after coronary artery surgery with isoflurane, sevoflurane, or target-controlled propofol anesthesia: a prospective, randomized, controlled trial. J Cardiothorac Vasc Anesth 2004;18(5):613-9. (In eng). DOI: 10.1053/j.jvca.2004.07.004.

58*. Arar C, Kaya G, Karamanlioğlu B, Pamukçu Z, Turan N. Effects of sevoflurane, isoflurane and propofol infusions on post-operative recovery criteria in geriatric patients. J Int Med Res 2005;33(1):55-60. (In eng). DOI: 10.1177/147323000503300104.

59*. Bein B, Renner J, Caliebe D, et al. Sevoflurane but not propofol preserves myocardial function during minimally invasive direct coronary artery bypass surgery. Anesth Analg 2005;100(3):610-616. (In eng). DOI: 10.1213/01.Ane.0000145012.27484.A7.

60. Corbett SM, Rebuck JA, Greene CM, et al. Dexmedetomidine does not improve patient satisfaction when compared with propofol during mechanical ventilation. Crit Care Med 2005;33(5):940-5. (In eng). DOI: 10.1097/01.ccm.0000162565.18193.e5.

61. Graziola E, Elena G, Gobbo M, Mendez F, Colucci D, Puig N. [Stress, hemodynamic and immunological responses to inhaled and intravenous anesthetic techniques for video-assisted laparoscopic cholecystectomy]. Rev Esp Anestesiol Reanim 2005;52(4):208-16. (In spa).

62. Malagon I, Hogenbirk K, van Pelt J, Hazekamp MG, Bovill JG. Effect of three different anaesthetic agents on the postoperative production of cardiac troponin T in paediatric cardiac surgery. Br J Anaesth 2005;94(6):805-9. (In eng). DOI: 10.1093/bja/aei142.

63. Prins SA, Peeters MY, Houmes RJ, et al. Propofol 6% as sedative in children under 2 years of age following major craniofacial surgery. Br J Anaesth 2005;94(5):630-5. (In eng). DOI: 10.1093/bja/aei104.

64. Carson SS, Kress JP, Rodgers JE, et al. A randomized trial of intermittent lorazepam versus propofol with daily interruption in mechanically ventilated patients. Crit Care Med 2006;34(5):1326-32. (In eng). DOI: 10.1097/01.Ccm.0000215513.63207.7f.

65. Collini S, Pinto G, Lejeune L, et al. Neurosedation in dentistry of the disabled patient: the use of midazolam, propofol, and remifentanil. Minerva Stomatol 2006;55(3):99-113. (In eng ita).

66*. Cromheecke S, Pepermans V, Hendrickx E, et al. Cardioprotective properties of sevoflurane in patients undergoing aortic valve replacement with cardiopulmonary bypass. Anesth Analg 2006;103(2):289-96, table of contents. (In eng). DOI: 10.1213/01.ane.0000226097.22384.f4.

67. Guarracino F, Landoni G, Tritapepe L, et al. Myocardial damage prevented by volatile anesthetics: a multicenter randomized controlled study. J Cardiothorac Vasc Anesth 2006;20(4):477-83. (In eng). DOI: 10.1053/j.jvca.2006.05.012.

68. Hanss R, Bein B, Turowski P, et al. The influence of xenon on regulation of the autonomic nervous system in patients at high risk of perioperative cardiac complications. Br J Anaesth 2006;96(4):427-36. (In eng). DOI: 10.1093/bja/ael028.

69*. Kostopanagiotou G, Pandazi A, Matiatou S, et al. The impact of intraoperative propofol administration in the prevention of postoperative pruritus induced by epidural morphine. Eur J Anaesthesiol 2006;23(5):418-21. (In eng). DOI: 10.1017/s0265021505001912.

70. Law-Koune JD, Raynaud C, Liu N, Dubois C, Romano M, Fischler M. Sevoflurane-remifentanil versus propofol-remifentanil anesthesia at a similar bispectral level for off-pump coronary artery surgery: no evidence of reduced myocardial ischemia. J Cardiothorac Vasc Anesth 2006;20(4):484-92. (In eng). DOI: 10.1053/j.jvca.2005.08.001.

71*. Lo YL, Dan YF, Tan YE, et al. Intraoperative motor-evoked potential monitoring in scoliosis surgery: comparison of desflurane/nitrous oxide with propofol total intravenous anesthetic regimens. J Neurosurg Anesthesiol 2006;18(3):211-4. (In eng). DOI: 10.1097/01.ana.0000211007.94269.50.

72. Lorsomradee S, Cromheecke S, Lorsomradee S, De Hert SG. Effects of sevoflurane on biomechanical markers of hepatic and renal dysfunction after coronary artery surgery. J Cardiothorac Vasc Anesth 2006;20(5):684-90. (In eng). DOI: 10.1053/j.jvca.2006.02.035.

73. Rabie M, Negmi H, Hammad Y, Al Oufi H, Khalaf H. Living donor hepatectomy (LDH)--comparative study between two different anesthetic techniques. Middle East J Anaesthesiol 2006;18(4):743-56. (In eng).

74. Shirakami G, Teratani Y, Fukuda K. Nocturnal episodic hypoxemia after ambulatory breast cancer surgery: comparison of sevoflurane and propofol-fentanyl anesthesia. J Anesth 2006;20(2):78-85. (In eng). DOI: 10.1007/s00540-005-0371-8.

75. Walldén J, Thörn SE, Lövqvist A, Wattwil L, Wattwil M. The effect of anesthetic technique on early postoperative gastric emptying: comparison of propofol-remifentanil and opioid-free sevoflurane anesthesia. J Anesth 2006;20(4):261-7. (In eng). DOI: 10.1007/s00540-006-0436-3.

76. Ghori KA, Harmon DC, Elashaal A, et al. Effect of midazolam versus propofol sedation on markers of neurological injury and outcome after isolated severe head injury: a pilot study. Crit Care Resusc 2007;9(2):166-71. (In eng).

77*. Musialowicz T, Niskanen M, Yppärilä-Wolters H, Pöyhönen M, Pitkänen O, Hynynen M. Auditory-evoked potentials in bispectral index-guided anaesthesia for cardiac surgery. Eur J Anaesthesiol 2007;24(7):571-9. (In eng). DOI: 10.1017/s0265021507000403.

78. Tritapepe L, Landoni G, Guarracino F, et al. Cardiac protection by volatile anaesthetics: a multicentre randomized controlled study in patients undergoing coronary artery bypass grafting with cardiopulmonary bypass. Eur J Anaesthesiol 2007;24(4):323-31. (In eng). DOI: 10.1017/s0265021506001931.

79. Vanacker BF, Vermeyen KM, Struys MM, et al. Reversal of rocuronium-induced neuromuscular block with the novel drug sugammadex is equally effective under maintenance anesthesia with propofol or sevoflurane. Anesth Analg 2007;104(3):563-8. (In eng). DOI: 10.1213/01.ane.0000231829.29177.8e.

80*. Bhagat H, Dash HH, Bithal PK, Chouhan RS, Pandia MP. Planning for early emergence in neurosurgical patients: a randomized prospective trial of low-dose anesthetics. Anesth Analg 2008;107(4):1348-55. (In eng). DOI: 10.1213/ane.0b013e31817f9476.

81. Cavalca V, Colli S, Veglia F, et al. Anesthetic propofol enhances plasma gamma-tocopherol levels in patients undergoing cardiac surgery. Anesthesiology 2008;108(6):988-97. (In eng). DOI: 10.1097/ALN.0b013e318173efb4.

82. Cheng SS, Yeh J, Flood P. Anesthesia matters: patients anesthetized with propofol have less postoperative pain than those anesthetized with isoflurane. Anesth Analg 2008;106(1):264-9, table of contents. (In eng). DOI: 10.1213/01.ane.0000287653.77372.d9.

83. Huey-Ling L, Chun-Che S, Jen-Jen T, Shau-Ting L, Hsing IC. Comparison of the effect of protocol-directed sedation with propofol vs. midazolam by nurses in intensive care: efficacy, haemodynamic stability and patient satisfaction. J Clin Nurs 2008;17(11):1510-7. (In eng). DOI: 10.1111/j.1365-2702.2007.02128.x.

84. Ko JS, Gwak MS, Choi SJ, et al. The effects of desflurane and propofol-remifentanil on postoperative hepatic and renal functions after right hepatectomy in liver donors. Liver Transpl 2008;14(8):1150-8. (In eng). DOI: 10.1002/lt.21490.

85. Okuyucu S, Inanoglu K, Akkurt CO, Akoglu E, Dagli S. The effect of anesthetic agents on perioperative bleeding during tonsillectomy: propofol-based versus desflurane-based anesthesia. Otolaryngol Head Neck Surg 2008;138(2):158-61. (In eng). DOI: 10.1016/j.otohns.2007.10.032.

86. Hasani A, Ozgen S, Baftiu N. Emergence agitation in children after propofol versus halothane anesthesia. Med Sci Monit 2009;15(6):Cr302-6. (In eng).

87*. Ionescu D, Mărgărit S, Vlad L, et al. [TIVA-TCI (Total IntraVenous Anesthesia-Target Controlled Infusion) versus isoflurane anesthesia for laparoscopic cholecystectomy. Postoperative nausea and vomiting, and patient satisfaction]. Chirurgia (Bucur) 2009;104(2):167-72. (In rum).

88. Kazanci D, Unver S, Karadeniz U, et al. A comparison of the effects of desflurane, sevoflurane and propofol on QT, QTc, and P dispersion on ECG. Ann Card Anaesth 2009;12(2):107-12. (In eng). DOI: 10.4103/0971-9784.51361.

89*. Khurana P, Agarwal A, Verma R, Gupta P. Comparison of Midazolam and Propofol for BIS-Guided Sedation During Regional Anaesthesia. Indian J Anaesth 2009;53(6):662-6. (In eng).

90. Maldonado JR, Wysong A, van der Starre PJ, Block T, Miller C, Reitz BA. Dexmedetomidine and the reduction of postoperative delirium after cardiac surgery. Psychosomatics 2009;50(3):206-17. (In eng). DOI: 10.1176/appi.psy.50.3.206.

91. Memiş D, Kargi M, Sut N. Effects of propofol and dexmedetomidine on indocyanine green elimination assessed with LIMON to patients with early septic shock: a pilot study. J Crit Care 2009;24(4):603-8. (In eng). DOI: 10.1016/j.jcrc.2008.10.005.

92. Tasdogan M, Memis D, Sut N, Yuksel M. Results of a pilot study on the effects of propofol and dexmedetomidine on inflammatory responses and intraabdominal pressure in severe sepsis. J Clin Anesth 2009;21(6):394-400. (In eng). DOI: 10.1016/j.jclinane.2008.10.010.

93. Yildirim V, Doganci S, Aydin A, Bolcal C, Demirkilic U, Cosar A. Cardioprotective effects of sevoflurane, isoflurane, and propofol in coronary surgery patients: a randomized controlled study. Heart Surg Forum 2009;12(1):E1-9. (In eng). DOI: 10.1532/hsf98.20081137.

94. Flier S, Post J, Concepcion AN, Kappen TH, Kalkman CJ, Buhre WF. Influence of propofol-opioid vs isoflurane-opioid anaesthesia on postoperative troponin release in patients undergoing coronary artery bypass grafting. Br J Anaesth 2010;105(2):122-30. (In eng). DOI: 10.1093/bja/aeq111.

95. Song JC, Sun YM, Yang LQ, Zhang MZ, Lu ZJ, Yu WF. A comparison of liver function after hepatectomy with inflow occlusion between sevoflurane and propofol anesthesia. Anesth Analg 2010;111(4):1036-41. (In eng). DOI: 10.1213/ANE.0b013e3181effda8.

96. Strøm T, Martinussen T, Toft P. A protocol of no sedation for critically ill patients receiving mechanical ventilation: a randomised trial. Lancet 2010;375(9713):475-80. (In eng). DOI: 10.1016/s0140-6736(09)62072-9.

97. Van der Linden PJ, Dierick A, Wilmin S, Bellens B, De Hert SG. A randomized controlled trial comparing an intraoperative goal-directed strategy with routine clinical practice in patients undergoing peripheral arterial surgery. Eur J Anaesthesiol 2010;27(9):788-93. (In eng). DOI: 10.1097/EJA.0b013e32833cb2dd.

98. Ballester M, Llorens J, Garcia-de-la-Asuncion J, et al. Myocardial oxidative stress protection by sevoflurane vs. propofol: a randomised controlled study in patients undergoing off-pump coronary artery bypass graft surgery. Eur J Anaesthesiol 2011;28(12):874-81. (In eng). DOI: 10.1097/EJA.0b013e32834bea2a.

99. Bignami E, Landoni G, Gerli C, et al. Sevoflurane vs. propofol in patients with coronary disease undergoing mitral surgery: a randomised study. Acta Anaesthesiol Scand 2012;56(4):482-90. (In eng). DOI: 10.1111/j.1399-6576.2011.02570.x.

100. Huang Z, Zhong X, Irwin MG, et al. Synergy of isoflurane preconditioning and propofol postconditioning reduces myocardial reperfusion injury in patients. Clin Sci (Lond) 2011;121(2):57-69. (In eng). DOI: 10.1042/cs20100435.

101. Rossetti AO, Milligan TA, Vulliémoz S, Michaelides C, Bertschi M, Lee JW. A randomized trial for the treatment of refractory status epilepticus. Neurocrit Care 2011;14(1):4-10. (In eng). DOI: 10.1007/s12028-010-9445-z.

102*. Royse CF, Andrews DT, Newman SN, et al. The influence of propofol or desflurane on postoperative cognitive dysfunction in patients undergoing coronary artery bypass surgery. Anaesthesia 2011;66(6):455-64. (In eng). DOI: 10.1111/j.1365-2044.2011.06704.x.

103. Schilling T, Kozian A, Senturk M, et al. Effects of volatile and intravenous anesthesia on the alveolar and systemic inflammatory response in thoracic surgical patients. Anesthesiology 2011;115(1):65-74. (In eng). DOI: 10.1097/ALN.0b013e318214b9de.

104. Schoen J, Husemann L, Tiemeyer C, et al. Cognitive function after sevoflurane- vs propofol-based anaesthesia for on-pump cardiac surgery: a randomized controlled trial. Br J Anaesth 2011;106(6):840-50. (In eng). DOI: 10.1093/bja/aer091.

105. Tempe DK, Dutta D, Garg M, Minhas H, Tomar A, Virmani S. Myocardial protection with isoflurane during off-pump coronary artery bypass grafting: a randomized trial. J Cardiothorac Vasc Anesth 2011;25(1):59-65. (In eng). DOI: 10.1053/j.jvca.2010.03.002.

106*. Uri O, Behrbalk E, Haim A, Kaufman E, Halpern P. Procedural sedation with propofol for painful orthopaedic manipulation in the emergency department expedites patient management compared with a midazolam/ketamine regimen: a randomized prospective study. J Bone Joint Surg Am 2011;93(24):2255-62. (In eng). DOI: 10.2106/jbjs.J.01307.

107. Zangrillo A, Testa V, Aldrovandi V, et al. Volatile agents for cardiac protection in noncardiac surgery: a randomized controlled study. J Cardiothorac Vasc Anesth 2011;25(6):902-7. (In eng). DOI: 10.1053/j.jvca.2011.06.016.

108*. Zoremba M, Dette F, Hunecke T, Eberhart L, Braunecker S, Wulf H. A comparison of desflurane versus propofol: the effects on early postoperative lung function in overweight patients. Anesth Analg 2011;113(1):63-9. (In eng). DOI: 10.1213/ANE.0b013e3181fdf5d4.

109. Bindra A, Chouhan RS, Prabhakar H, Dash HH, Chandra PS, Tripathi M. Comparison of the effects of different anesthetic techniques on electrocorticography in patients undergoing epilepsy surgery - a bispectral index guided study. Seizure 2012;21(7):501-7. (In eng). DOI: 10.1016/j.seizure.2012.05.002.

110*. Bjelland TW, Dale O, Kaisen K, et al. Propofol and remifentanil versus midazolam and fentanyl for sedation during therapeutic hypothermia after cardiac arrest: a randomised trial. Intensive Care Med 2012;38(6):959-67. (In eng). DOI: 10.1007/s00134-012-2540-1.

111*. Chi X, Chen Y, Liao M, Cao F, Tian Y, Wang X. Comparative cost analysis of three different anesthesia methods in gynecological laparoscopic surgery. Front Med 2012;6(3):311-6. (In eng). DOI: 10.1007/s11684-012-0205-7.

112*. De La Mora-González JF, Robles-Cervantes JA, Mora-Martínez JM, et al. Hemodynamic effects of dexmedetomidine--fentanyl vs. nalbuphine--propofol in plastic surgery. Middle East J Anaesthesiol 2012;21(4):553-7. (In eng).

113. Jakob SM, Ruokonen E, Grounds RM, et al. Dexmedetomidine vs midazolam or propofol for sedation during prolonged mechanical ventilation: two randomized controlled trials. Jama 2012;307(11):1151-60. (In eng). DOI: 10.1001/jama.2012.304.

114. Jovic M, Stancic A, Nenadic D, et al. Mitochondrial molecular basis of sevoflurane and propofol cardioprotection in patients undergoing aortic valve replacement with cardiopulmonary bypass. Cell Physiol Biochem 2012;29(1-2):131-42. (In eng). DOI: 10.1159/000337594.

115*. Konstantopoulos K, Makris A, Moustaka A, Karmaniolou I, Konstantopoulos G, Mela A. Sevoflurane versus propofol anesthesia in patients undergoing lumbar spondylodesis: a randomized trial. J Surg Res 2013;179(1):72-7. (In eng). DOI: 10.1016/j.jss.2012.09.038.

116. Kottenberg E, Thielmann M, Bergmann L, et al. Protection by remote ischemic preconditioning during coronary artery bypass graft surgery with isoflurane but not propofol - a clinical trial. Acta Anaesthesiol Scand 2012;56(1):30-8. (In eng). DOI: 10.1111/j.1399-6576.2011.02585.x.

117*. Laviolle B, Basquin C, Aguillon D, et al. Effect of an anesthesia with propofol compared with desflurane on free radical production and liver function after partial hepatectomy. Fundam Clin Pharmacol 2012;26(6):735-42. (In eng). DOI: 10.1111/j.1472-8206.2011.00958.x.

118. Lurati Buse GA, Schumacher P, Seeberger E, et al. Randomized comparison of sevoflurane versus propofol to reduce perioperative myocardial ischemia in patients undergoing noncardiac surgery. Circulation 2012;126(23):2696-704. (In eng). DOI: 10.1161/circulationaha.112.126144.

119. Song JG, Shin JW, Lee EH, et al. Incidence of post-thoracotomy pain: a comparison between total intravenous anaesthesia and inhalation anaesthesia. Eur J Cardiothorac Surg 2012;41(5):1078-82. (In eng). DOI: 10.1093/ejcts/ezr133.

120. Soro M, Gallego L, Silva V, et al. Cardioprotective effect of sevoflurane and propofol during anaesthesia and the postoperative period in coronary bypass graft surgery: a double-blind randomised study. Eur J Anaesthesiol 2012;29(12):561-9. (In eng). DOI: 10.1097/EJA.0b013e3283560aea.

121. Tanguy M, Seguin P, Laviolle B, Bleichner JP, Morandi X, Malledant Y. Cerebral microdialysis effects of propofol versus midazolam in severe traumatic brain injury. J Neurotrauma 2012;29(6):1105-10. (In eng). DOI: 10.1089/neu.2011.1817.

122. Terao Y, Ichinomiya T, Higashijima U, et al. Comparison between propofol and dexmedetomidine in postoperative sedation after extensive cervical spine surgery. J Anesth 2012;26(2):179-86. (In eng). DOI: 10.1007/s00540-011-1300-7.

123*. Wu J, Yao S, Wu Z, et al. A comparison of anesthetic regimens using etomidate and propofol in patients undergoing first-trimester abortions: double-blind, randomized clinical trial of safety and efficacy. Contraception 2013;87(1):55-62. (In eng). DOI: 10.1016/j.contraception.2012.08.014.

124*. Möller Petrun A, Kamenik M. Bispectral index-guided induction of general anaesthesia in patients undergoing major abdominal surgery using propofol or etomidate: a double-blind, randomized, clinical trial. Br J Anaesth 2013;110(3):388-96. (In eng). DOI: 10.1093/bja/aes416.

125*. Nagao Y, Tatara T, Fujita K, Sugi T, Kotani J, Hirose M. Protein sparing during general anesthesia with a propofol solution containing medium-chain triglycerides for gastrectomy: comparison with sevoflurane anesthesia. J Anesth 2013;27(3):359-65. (In eng). DOI: 10.1007/s00540-012-1546-8.

126*. Baki ED, Aldemir M, Kokulu S, et al. Comparison of the effects of desflurane and propofol anesthesia on the inflammatory response and s100β protein during coronary artery bypass grafting. Inflammation 2013;36(6):1327-33. (In eng). DOI: 10.1007/s10753-013-9671-6.

127*. Braz MG, Braz LG, Braz JR, et al. Comparison of oxidative stress in ASA physical status I patients scheduled for minimally invasive surgery under balanced or intravenous anesthesia. Minerva Anestesiol 2013;79(9):1030-8. (In eng).

128*. Chaaban MR, Baroody FM, Gottlieb O, Naclerio RM. Blood loss during endoscopic sinus surgery with propofol or sevoflurane: a randomized clinical trial. JAMA Otolaryngol Head Neck Surg 2013;139(5):510-4. (In eng). DOI: 10.1001/jamaoto.2013.2885.

129*. Dahaba AA, Yin J, Xiao Z, et al. Different propofol-remifentanil or sevoflurane-remifentanil bispectral index levels for electrocorticographic spike identification during epilepsy surgery. Anesthesiology 2013;119(3):582-92. (In eng). DOI: 10.1097/ALN.0b013e3182976036.

130*. Kalimeris K, Kouni S, Kostopanagiotou G, et al. Cognitive function and oxidative stress after carotid endarterectomy: comparison of propofol to sevoflurane anesthesia. J Cardiothorac Vasc Anesth 2013;27(6):1246-52. (In eng). DOI: 10.1053/j.jvca.2012.12.009.

131*. Kowalczyk M, Fijałkowska A, Nestorowicz A. New generation pulse oximetry in the assessment of peripheral perfusion during general anaesthesia - a comparison between propofol and desflurane. Anaesthesiol Intensive Ther 2013;45(3):138-44. (In eng). DOI: 10.5603/ait.2013.0029.

132. Lindholm EE, Aune E, Norén CB, et al. The anesthesia in abdominal aortic surgery (ABSENT) study: a prospective, randomized, controlled trial comparing troponin T release with fentanyl-sevoflurane and propofol-remifentanil anesthesia in major vascular surgery. Anesthesiology 2013;119(4):802-12. (In eng). DOI: 10.1097/ALN.0b013e31829bd883.

133. Mazoti MA, Braz MG, de Assis Golim M, et al. Comparison of inflammatory cytokine profiles in plasma of patients undergoing otorhinological surgery with propofol or isoflurane anesthesia. Inflamm Res 2013;62(10):879-85. (In eng). DOI: 10.1007/s00011-013-0643-y.

134*. Mencke T, Zitzmann A, Machmueller S, et al. Anesthesia with Propofol versus Sevoflurane: Does the Longer Neuromuscular Block under Sevoflurane Anesthesia Reduce Laryngeal Injuries? Anesthesiol Res Pract 2013;2013:723168. (In eng). DOI: 10.1155/2013/723168.

135. Oztürk I, Serin S, Gürses E. Biochemical markers in total intravenous anesthesia and propofol infusion syndrome: a preliminary study. Eur Rev Med Pharmacol Sci 2013;17(24):3385-90. (In eng).

136. Sofra M, Fei PC, Fabrizi L, et al. Immunomodulatory effects of total intravenous and balanced inhalation anesthesia in patients with bladder cancer undergoing elective radical cystectomy: preliminary results. J Exp Clin Cancer Res 2013;32(1):6. (In eng). DOI: 10.1186/1756-9966-32-6.

137. Song JC, Zhang MZ, Wu QC, et al. Sevoflurane has no adverse effects on renal function in cirrhotic patients: a comparison with propofol. Acta Anaesthesiol Scand 2013;57(7):896-902. (In eng). DOI: 10.1111/aas.12085.

138*. Valencia L, Rodríguez-Pérez A, Kühlmorgen B, Santana RY. Does sevoflurane preserve regional cerebral oxygen saturation measured by near-infrared spectroscopy better than propofol? Ann Fr Anesth Reanim 2014;33(4):e59-65. (In eng). DOI: 10.1016/j.annfar.2013.12.020.

139. Xu WY, Wang N, Xu HT, et al. Effects of sevoflurane and propofol on right ventricular function and pulmonary circulation in patients undergone esophagectomy. Int J Clin Exp Pathol 2014;7(1):272-9. (In eng).

140*. Ziemann-Gimmel P, Goldfarb AA, Koppman J, Marema RT. Opioid-free total intravenous anaesthesia reduces postoperative nausea and vomiting in bariatric surgery beyond triple prophylaxis. Br J Anaesth 2014;112(5):906-11. (In eng). DOI: 10.1093/bja/aet551.

141. Chen J, Zhou JQ, Chen ZF, Huang Y, Jiang H. Efficacy and safety of dexmedetomidine versus propofol for the sedation of tube-retention after oral maxillofacial surgery. J Oral Maxillofac Surg 2014;72(2):285.e1-7. (In eng). DOI: 10.1016/j.joms.2013.10.006.

142. Erturk E, Topaloglu S, Dohman D, et al. The comparison of the effects of sevoflurane inhalation anesthesia and intravenous propofol anesthesia on oxidative stress in one lung ventilation. Biomed Res Int 2014;2014:360936. (In eng). DOI: 10.1155/2014/360936.

143. Grendelmeier P, Tamm M, Jahn K, Pflimlin E, Stolz D. Propofol versus midazolam in medical thoracoscopy: a randomized, noninferiority trial. Respiration 2014;88(2):126-36. (In eng). DOI: 10.1159/000362797.

144. Liang C, Ding M, Du F, Cang J, Xue Z. Sevoflurane/propofol coadministration provides better recovery than sevoflurane in combined general/epidural anesthesia: a randomized clinical trial. J Anesth 2014;28(5):721-6. (In eng). DOI: 10.1007/s00540-014-1803-0.

145. Margarit SC, Vasian HN, Balla E, Vesa S, Ionescu DC. The influence of total intravenous anaesthesia and isoflurane anaesthesia on plasma interleukin-6 and interleukin-10 concentrations after colorectal surgery for cancer: a randomised controlled trial. Eur J Anaesthesiol 2014;31(12):678-84. (In eng). DOI: 10.1097/eja.0000000000000057.

146*. Mroziński P, Lango R, Biedrzycka A, Kowalik MM, Pawlaczyk R, Rogowski J. Comparison of haemodynamics and myocardial injury markers under desflurane vs. propofol anaesthesia for off-pump coronary surgery. A prospective randomised trial. Anaesthesiol Intensive Ther 2014;46(1):4-13. (In eng). DOI: 10.5603/ait.2014.0002.

147*. Ortiz J, Chang LC, Tolpin DA, Minard CG, Scott BG, Rivers JM. Randomized, controlled trial comparing the effects of anesthesia with propofol, isoflurane, desflurane and sevoflurane on pain after laparoscopic cholecystectomy. Braz J Anesthesiol 2014;64(3):145-51. (In eng). DOI: 10.1016/j.bjane.2013.03.011.

148*. Parida S, Badhe AS. Comparison of cognitive, ambulatory, and psychomotor recovery profiles after day care anesthesia with propofol and sevoflurane. J Anesth 2014;28(6):833-8. (In eng). DOI: 10.1007/s00540-014-1827-5.

149. Rozec B, Floch H, Berlivet P, Michel P, Blanloeil Y. Propofol versus thiopental by target controlled infusion in patients undergoing craniotomy. Minerva Anestesiol 2014;80(7):761-8. (In eng).

150. Shah PN, Dongre V, Patil V, Pandya S, Mungantiwar A, Choulwar A. Comparison of post-operative ICU sedation between dexmedetomidine and propofol in Indian population. Indian J Crit Care Med 2014;18(5):291-6. (In eng). DOI: 10.4103/0972-5229.132485.

151. Yoo YC, Shim JK, Song Y, Yang SY, Kwak YL. Anesthetics influence the incidence of acute kidney injury following valvular heart surgery. Kidney Int 2014;86(2):414-22. (In eng). DOI: 10.1038/ki.2013.532.

152*. Yu JB, Dong SA, Gong LR, et al. Effect of electroacupuncture at Zusanli (ST36) and Sanyinjiao (SP6) acupoints on adrenocortical function in etomidate anesthesia patients. Med Sci Monit 2014;20:406-12. (In eng). DOI: 10.12659/msm.890111.

153. Zhou Y, Jin X, Kang Y, Liang G, Liu T, Deng N. Midazolam and propofol used alone or sequentially for long-term sedation in critically ill, mechanically ventilated patients: a prospective, randomized study. Crit Care 2014;18(3):R122. (In eng). DOI: 10.1186/cc13922.

154. Bastola P, Bhagat H, Wig J. Comparative evaluation of propofol, sevoflurane and desflurane for neuroanaesthesia: A prospective randomised study in patients undergoing elective supratentorial craniotomy. Indian J Anaesth 2015;59(5):287-94. (In eng). DOI: 10.4103/0019-5049.156868.

155. Dabir S, Mohammad-Taheri Z, Parsa T, Abbasi-Nazari M, Radpay B, Radmand G. Effects of propofol versus isoflurane on liver function after open thoracotomy. Asian Cardiovasc Thorac Ann 2015;23(3):292-8. (In eng). DOI: 10.1177/0218492314551972.

156*. Goswami U, Babbar S, Tiwari S. Comparative evaluation of the effects of propofol and sevoflurane on cognitive function and memory in patients undergoing laparoscopic cholecystectomy: A randomised prospective study. Indian J Anaesth 2015;59(3):150-5. (In eng). DOI: 10.4103/0019-5049.153036.

157. Jia L, Dong R, Zhang F, et al. Propofol Provides More Effective Protection for Circulating Lymphocytes Than Sevoflurane in Patients Undergoing Off-Pump Coronary Artery Bypass Graft Surgery. J Cardiothorac Vasc Anesth 2015;29(5):1172-9. (In eng). DOI: 10.1053/j.jvca.2015.01.008.

158. Karaman Y, Abud B, Tekgul ZT, Cakmak M, Yildiz M, Gonullu M. Effects of dexmedetomidine and propofol on sedation in patients after coronary artery bypass graft surgery in a fast-track recovery room setting. J Anesth 2015;29(4):522-8. (In eng). DOI: 10.1007/s00540-015-1975-2.

159. Kim YS, Lim BG, Kim H, Kong MH, Lee IO. Effects of propofol or desflurane on post-operative spirometry in elderly after knee surgery: a double-blind randomised study. Acta Anaesthesiol Scand 2015;59(6):788-95. (In eng). DOI: 10.1111/aas.12494.

160. Lehavi A, Sandler O, Mahajna A, Weissman A, Katz YS. Comparison of Rhabdomyolysis Markers in Patients Undergoing Bariatric Surgery with Propofol and Inhalation-based Anesthesia. Obes Surg 2015;25(10):1923-7. (In eng). DOI: 10.1007/s11695-015-1626-6.

161. Ammar AS, Mahmoud KM. Comparative effect of propofol versus sevoflurane on renal ischemia/reperfusion injury after elective open abdominal aortic aneurysm repair. Saudi J Anaesth 2016;10(3):301-7. (In eng). DOI: 10.4103/1658-354x.174907.

162*. Beck-Schimmer B, Bonvini JM, Braun J, et al. Which Anesthesia Regimen Is Best to Reduce Morbidity and Mortality in Lung Surgery?: A Multicenter Randomized Controlled Trial. Anesthesiology 2016;125(2):313-21. (In eng). DOI: 10.1097/aln.0000000000001164.

163*. Bhakta P, Ghosh BR, Singh U, et al. Incidence of postoperative nausea and vomiting following gynecological laparoscopy: A comparison of standard anesthetic technique and propofol infusion. Acta Anaesthesiol Taiwan 2016;54(4):108-113. (In eng). DOI: 10.1016/j.aat.2016.10.002.

164*. Biedrzycka A, Kowalik M, Pawlaczyk R, et al. Aortic cross-clamping phase of cardiopulmonary bypass is related to decreased microvascular reactivity after short-term ischaemia of the thenar muscle both under intravenous and volatile anaesthesia: a randomized trial. Interact Cardiovasc Thorac Surg 2016;23(5):770-778. (In eng). DOI: 10.1093/icvts/ivw232.

165. Conti G, Ranieri VM, Costa R, et al. Effects of dexmedetomidine and propofol on patient-ventilator interaction in difficult-to-wean, mechanically ventilated patients: a prospective, open-label, randomised, multicentre study. Crit Care 2016;20(1):206. (In eng). DOI: 10.1186/s13054-016-1386-2.

166. Djaiani G, Silverton N, Fedorko L, et al. Dexmedetomidine versus Propofol Sedation Reduces Delirium after Cardiac Surgery: A Randomized Controlled Trial. Anesthesiology 2016;124(2):362-8. (In eng). DOI: 10.1097/aln.0000000000000951.

167. Doe A, Kumagai M, Tamura Y, Sakai A, Suzuki K. A comparative analysis of the effects of sevoflurane and propofol on cerebral oxygenation during steep Trendelenburg position and pneumoperitoneum for robotic-assisted laparoscopic prostatectomy. J Anesth 2016;30(6):949-955. (In eng). DOI: 10.1007/s00540-016-2241-y.

168. Erdem AF, Sahin YN, Dogan N, et al. Effects of sevoflurane and propofol on S100β and neuron-specific enolase protein levels during cardiopulmonary bypass. Niger J Clin Pract 2016;19(2):278-83. (In eng). DOI: 10.4103/1119-3077.164346.

169. Goettel N, Bharadwaj S, Venkatraghavan L, Mehta J, Bernstein M, Manninen PH. Dexmedetomidine vs propofol-remifentanil conscious sedation for awake craniotomy: a prospective randomized controlled trial. Br J Anaesth 2016;116(6):811-21. (In eng). DOI: 10.1093/bja/aew024.

170. Khalil M, Al-Agaty A, Asaad O, et al. A comparative study between propofol and dexmedetomidine as sedative agents during performing transcatheter aortic valve implantation. J Clin Anesth 2016;32:242-7. (In eng). DOI: 10.1016/j.jclinane.2016.03.014.

171. Likhvantsev VV, Landoni G, Levikov DI, Grebenchikov OA, Skripkin YV, Cherpakov RA. Sevoflurane Versus Total Intravenous Anesthesia for Isolated Coronary Artery Bypass Surgery With Cardiopulmonary Bypass: A Randomized Trial. J Cardiothorac Vasc Anesth 2016;30(5):1221-7. (In eng). DOI: 10.1053/j.jvca.2016.02.030.

172. Liu S, Gu X, Zhu L, et al. Effects of propofol and sevoflurane on perioperative immune response in patients undergoing laparoscopic radical hysterectomy for cervical cancer. Medicine (Baltimore) 2016;95(49):e5479. (In eng). DOI: 10.1097/md.0000000000005479.

173. Liu X, Zhang K, Wang W, Xie G, Fang X. Dexmedetomidine sedation reduces atrial fibrillation after cardiac surgery compared to propofol: a randomized controlled trial. Crit Care 2016;20(1):298. (In eng). DOI: 10.1186/s13054-016-1480-5.

174. Markovic-Bozic J, Karpe B, Potocnik I, Jerin A, Vranic A, Novak-Jankovic V. Effect of propofol and sevoflurane on the inflammatory response of patients undergoing craniotomy. BMC Anesthesiol 2016;16:18. (In eng). DOI: 10.1186/s12871-016-0182-5.

175. Moro ET, Leme FC, Noronha BR, Saraiva GF, de Matos Leite NV, Navarro LH. Quality of recovery from anesthesia of patients undergoing balanced or total intravenous general anesthesia. Prospective randomized clinical trial. J Clin Anesth 2016;35:369-375. (In eng). DOI: 10.1016/j.jclinane.2016.08.022.

176. Shah PJ, Dubey KP, Sahare KK, Agrawal A. Intravenous dexmedetomidine versus propofol for intraoperative moderate sedation during spinal anesthesia: A comparative study. J Anaesthesiol Clin Pharmacol 2016;32(2):245-9. (In eng). DOI: 10.4103/0970-9185.168172.

177. Cho YJ, Kim TK, Hong DM, Seo JH, Bahk JH, Jeon Y. Effect of desflurane-remifentanil vs. Propofol-remifentanil anesthesia on arterial oxygenation during one-lung ventilation for thoracoscopic surgery: a prospective randomized trial. BMC Anesthesiol 2017;17(1):9. (In eng). DOI: 10.1186/s12871-017-0302-x.

178. de la Gala F, Piñeiro P, Reyes A, et al. Postoperative pulmonary complications, pulmonary and systemic inflammatory responses after lung resection surgery with prolonged one-lung ventilation. Randomized controlled trial comparing intravenous and inhalational anaesthesia. Br J Anaesth 2017;119(4):655-663. (In eng). DOI: 10.1093/bja/aex230.

179. Guerrero Orriach JL, Galán Ortega M, Ramirez Fernandez A, et al. Cardioprotective efficacy of sevoflurane vs. propofol during induction and/or maintenance in patients undergoing coronary artery revascularization surgery without pump: A randomized trial. Int J Cardiol 2017;243:73-80. (In eng). DOI: 10.1016/j.ijcard.2017.04.105.

180. Hassan W, Nasir YM, Zaini RHM, Shukeri W. Target-controlled Infusion Propofol Versus Sevoflurane Anaesthesia for Emergency Traumatic Brain Surgery: Comparison of the Outcomes. Malays J Med Sci 2017;24(5):73-82. (In eng). DOI: 10.21315/mjms2017.24.5.8.

181. Hofland J, Ouattara A, Fellahi JL, et al. Effect of Xenon Anesthesia Compared to Sevoflurane and Total Intravenous Anesthesia for Coronary Artery Bypass Graft Surgery on Postoperative Cardiac Troponin Release: An International, Multicenter, Phase 3, Single-blinded, Randomized Noninferiority Trial. Anesthesiology 2017;127(6):918-933. (In eng). DOI: 10.1097/aln.0000000000001873.

182. Nieuwenhuijs-Moeke GJ, Nieuwenhuijs VB, Seelen MAJ, et al. Propofol-based anaesthesia versus sevoflurane-based anaesthesia for living donor kidney transplantation: results of the VAPOR-1 randomized controlled trial. Br J Anaesth 2017;118(5):720-732. (In eng). DOI: 10.1093/bja/aex057.

183. Tanaka P, Goodman S, Sommer BR, Maloney W, Huddleston J, Lemmens HJ. The effect of desflurane versus propofol anesthesia on postoperative delirium in elderly obese patients undergoing total knee replacement: A randomized, controlled, double-blinded clinical trial. J Clin Anesth 2017;39:17-22. (In eng). DOI: 10.1016/j.jclinane.2017.03.015.

184. Yang XL, Wang D, Zhang GY, Guo XL. Comparison of the myocardial protective effect of sevoflurane versus propofol in patients undergoing heart valve replacement surgery with cardiopulmonary bypass. BMC Anesthesiol 2017;17(1):37. (In eng). DOI: 10.1186/s12871-017-0326-2.

185. Zhang Y, Lin W, Shen S, Wang H, Feng X, Sun J. Randomized comparison of sevoflurane versus propofol-remifentanil on the cardioprotective effects in elderly patients with coronary heart disease. BMC Anesthesiol 2017;17(1):104. (In eng). DOI: 10.1186/s12871-017-0397-0.

186. Eshghpour M, Samieirad S, Attar AS, Kermani H, Seddigh S. Propofol Versus Remifentanil: Which One Is More Effective in Reducing Blood Loss During Orthognathic Surgery? A Randomized Clinical Trial. J Oral Maxillofac Surg 2018;76(9):1882.e1-1882.e7. (In eng). DOI: 10.1016/j.joms.2018.05.012.

187. Ji FH, Wang D, Zhang J, Liu HY, Peng K. Effects of propofol anesthesia versus sevoflurane anesthesia on postoperative pain after radical gastrectomy: a randomized controlled trial. J Pain Res 2018;11:1247-1254. (In eng). DOI: 10.2147/jpr.S164889.

188. Kuzkov VV, Obraztsov MY, Ivashchenko OY, Ivashchenko NY, Gorenkov VM, Kirov MY. Total Intravenous Versus Volatile Induction and Maintenance of Anesthesia in Elective Carotid Endarterectomy: Effects on Cerebral Oxygenation and Cognitive Functions. J Cardiothorac Vasc Anesth 2018;32(4):1701-1708. (In eng). DOI: 10.1053/j.jvca.2017.12.049.

189. Little M, Tran V, Chiarella A, Wright ED. Total intravenous anesthesia vs inhaled anesthetic for intraoperative visualization during endoscopic sinus surgery: a double blind randomized controlled trial. Int Forum Allergy Rhinol 2018;8(10):1123-1126. (In eng). DOI: 10.1002/alr.22129.

190. Mei B, Meng G, Xu G, et al. Intraoperative Sedation With Dexmedetomidine is Superior to Propofol for Elderly Patients Undergoing Hip Arthroplasty: A Prospective Randomized Controlled Study. Clin J Pain 2018;34(9):811-817. (In eng). DOI: 10.1097/ajp.0000000000000605.

191. Moscarelli M, Terrasini N, Nunziata A, et al. A Trial of Two Anesthetic Regimes for Minimally Invasive Mitral Valve Repair. J Cardiothorac Vasc Anesth 2018;32(6):2562-2569. (In eng). DOI: 10.1053/j.jvca.2018.01.028.

192. Oh CS, Kim K, Kang WS, et al. Comparison of the expression of cluster of differentiation (CD)39 and CD73 between propofol- and sevoflurane-based anaesthesia during open heart surgery. Sci Rep 2018;8(1):10197. (In eng). DOI: 10.1038/s41598-018-28505-8.

193. Sheikh TA, Dar BA, Akhter N, Ahmad N. A Comparative Study Evaluating Effects of Intravenous Sedation by Dexmedetomidine and Propofol on Patient Hemodynamics and Postoperative Outcomes in Cardiac Surgery. Anesth Essays Res 2018;12(2):555-560. (In eng). DOI: 10.4103/aer.AER_46_18.

194. Mark S, Ebtesam I, Kenneth N. Comparison of dexmedetomidine and propofol in mechanically ventilated patients with sepsis: A pilot study. The Southwest Respiratory and Critical Care Chronicles 2018;6(22). DOI: 10.12746/swrccc.v6i22.444.

195. Wąsowicz M, Jerath A, Luksun W, et al. Comparison of propofol-based versus volatile-based anaesthesia and postoperative sedation in cardiac surgical patients: a prospective, randomized, study. Anaesthesiol Intensive Ther 2018;50(3):200-209. (In eng). DOI: 10.5603/AIT.a2018.0012.

196. Wojarska-Tręda E, Olejnik K, Stojcev Z, Białka S, Misiołek H. Choosing the optimal method of anaesthesia in anterior resection of the rectum procedures - assessment of the stress reaction based on selected hormonal parameters. Endokrynol Pol 2018;69(4) (In eng). DOI: 10.5603/EP.a2018.0038.

197. Zhang Y, Shan GJ, Zhang YX, et al. Propofol compared with sevoflurane general anaesthesia is associated with decreased delayed neurocognitive recovery in older adults. Br J Anaesth 2018;121(3):595-604. (In eng). DOI: 10.1016/j.bja.2018.05.059.

198. Aditianingsih D, Sukmono B, Agung TA, Kartolo WY, Adiwongso ES, Mochtar CA. Comparison of the Effects of Target-Controlled Infusion of Propofol and Sevoflurane as Maintenance of Anesthesia on Hemodynamic Profile in Kidney Transplantation. Anesthesiol Res Pract 2019;2019:5629371. (In eng). DOI: 10.1155/2019/5629371.

199. Hahm TS, Jeong H, Ahn HJ. Systemic Oxygen Delivery during One-Lung Ventilation: Comparison between Propofol and Sevoflurane Anaesthesia in a Randomised Controlled Trial. J Clin Med 2019;8(9) (In eng). DOI: 10.3390/jcm8091438.

200. Jo JY, Jung KW, Kim HJ, et al. Effect of Total Intravenous Anesthesia vs Volatile Induction With Maintenance Anesthesia on Emergence Agitation After Nasal Surgery: A Randomized Clinical Trial. JAMA Otolaryngol Head Neck Surg 2019;145(2):117-123. (In eng). DOI: 10.1001/jamaoto.2018.3097.

201. Kim D, Jeong JS, Park H, et al. Postoperative pain control after the use of dexmedetomidine and propofol to sedate patients undergoing ankle surgery under spinal anesthesia: a randomized controlled trial. J Pain Res 2019;12:1479-1487. (In eng). DOI: 10.2147/jpr.S195745.

202. Lin WL, Lee MS, Wong CS, et al. Effects of intraoperative propofol-based total intravenous anesthesia on postoperative pain in spine surgery: Comparison with desflurane anesthesia - a randomised trial. Medicine (Baltimore) 2019;98(13):e15074. (In eng). DOI: 10.1097/md.0000000000015074.

203. Roh GU, Song Y, Park J, Ki YM, Han DW. Effects of propofol on the inflammatory response during robot-assisted laparoscopic radical prostatectomy: a prospective randomized controlled study. Sci Rep 2019;9(1):5242. (In eng). DOI: 10.1038/s41598-019-41708-x.

204*. Sahoo AK, Panda N, Sabharwal P, et al. Effect of Anesthetic Agents on Cognitive Function and Peripheral Inflammatory Biomarkers in Young Patients Undergoing Surgery for Spine Disorders. Asian J Neurosurg 2019;14(4):1095-1105. (In eng). DOI: 10.4103/ajns.AJNS_173_19.

205. Shi Y, Wang W. Application of different anesthetic methods in coronary artery bypass grafting and the effect on postoperative outcome. Exp Ther Med 2019;17(1):695-700. (In eng). DOI: 10.3892/etm.2018.6993.

206. Velayutham P, Cherian VT, Rajshekhar V, Babu KS. The effects of propofol and isoflurane on intraoperative motor evoked potentials during spinal cord tumour removal surgery - A prospective randomised trial. Indian J Anaesth 2019;63(2):92-99. (In eng). DOI: 10.4103/ija.IJA_421_18.

207. Wang W, Liu Y, Liu Y, Liu F, Ma Y. Comparison of Cognitive Impairments After Intensive Care Unit Sedation Using Dexmedetomidine and Propofol Among Older Patients. J Clin Pharmacol 2019;59(6):821-828. (In eng). DOI: 10.1002/jcph.1372.

208. Wu B, Hu H, Cai A, Ren C, Liu S. The safety and efficacy of dexmedetomidine versus propofol for patients undergoing endovascular therapy for acute stroke: A prospective randomized control trial. Medicine (Baltimore) 2019;98(21):e15709. (In eng). DOI: 10.1097/md.0000000000015709.

209. Wu ZF, Lin WL, Lee MS, et al. Propofol vs desflurane on the cytokine, matrix metalloproteinase-9, and heme oxygenase-1 response during living donor liver transplantation: A pilot study. Medicine (Baltimore) 2019;98(48):e18244. (In eng). DOI: 10.1097/md.0000000000018244.

210. Doi M, Morita K, Takeda J, Sakamoto A, Yamakage M, Suzuki T. Efficacy and safety of remimazolam versus propofol for general anesthesia: a multicenter, single-blind, randomized, parallel-group, phase IIb/III trial. J Anesth 2020;34(4):543-553. (In eng). DOI: 10.1007/s00540-020-02788-6.

211*. Efremov SM, Kozireva VS, Moroz GB, et al. The immunosuppressive effects of volatile versus intravenous anesthesia combined with epidural analgesia on kidney cancer: a pilot randomized controlled trial. Korean J Anesthesiol 2020;73(6):525-533. (In eng). DOI: 10.4097/kja.19461.

212*. Gollapudy S, Gashkoff DA, Poetker DM, Loehrl TA, Riess ML. Surgical Field Visualization during Functional Endoscopic Sinus Surgery: Comparison of Propofol- vs Desflurane-Based Anesthesia. Otolaryngol Head Neck Surg 2020;163(4):835-842. (In eng). DOI: 10.1177/0194599820921863.

213. Guinot PG, Ellouze O, Grosjean S, et al. Anaesthesia and ICU sedation with sevoflurane do not reduce myocardial injury in patients undergoing cardiac surgery: A randomized prospective study. Medicine (Baltimore) 2020;99(50):e23253. (In eng). DOI: 10.1097/md.0000000000023253.

214. Guo L, Lin F, Dai H, et al. Impact of Sevoflurane Versus Propofol Anesthesia on Post-Operative Cognitive Dysfunction in Elderly Cancer Patients: A Double-Blinded Randomized Controlled Trial. Med Sci Monit 2020;26:e919293. (In eng). DOI: 10.12659/msm.919293.

215. Haldar R, Kannaujia AK, Verma R, et al. Randomized Trial to Compare Plasma Glucose Trends in Patients Undergoing Surgery for Supratentorial Gliomas under Maintenance of Sevoflurane, Desflurane, and Propofol. Asian J Neurosurg 2020;15(3):579-586. (In eng). DOI: 10.4103/ajns.AJNS_235_20.

216*. Hovaguimian F, Braun J, Z'Graggen B R, et al. Anesthesia and Circulating Tumor Cells in Primary Breast Cancer Patients: A Randomized Controlled Trial. Anesthesiology 2020;133(3):548-558. (In eng). DOI: 10.1097/aln.0000000000003409.

217. Li X, Zhang B, Yu L, Yang J, Tan H. Influence of Sevoflurane-Based Anesthesia versus Total Intravenous Anesthesia on Intraoperative Neuromonitoring during Thyroidectomy. Otolaryngol Head Neck Surg 2020;162(6):853-859. (In eng). DOI: 10.1177/0194599820912030.

218. Liu J, Shi K, Hong J, et al. Dexmedetomidine protects against acute kidney injury in patients with septic shock. Ann Palliat Med 2020;9(2):224-230. (In eng). DOI: 10.21037/apm.2020.02.08.

219*. Mei B, Xu G, Han W, et al. The Benefit of Dexmedetomidine on Postoperative Cognitive Function Is Unrelated to the Modulation on Peripheral Inflammation: A Single-center, Prospective, Randomized Study. Clin J Pain 2020;36(2):88-95. (In eng). DOI: 10.1097/ajp.0000000000000779.

220. Mei X, Zheng HL, Li C, et al. The Effects of Propofol and Sevoflurane on Postoperative Delirium in Older Patients: A Randomized Clinical Trial Study. J Alzheimers Dis 2020;76(4):1627-1636. (In eng). DOI: 10.3233/jad-200322.

221*. Pandit A, Singh V, Bharati SJ, Mishra S, Deo SV, Bhatnagar S. A pilot randomised clinical trial comparing desflurane anaesthesia vs total intravenous anaesthesia, for changes in haemodynamic, inflammatory and coagulation parameters in patients undergoing hyperthermic intraperitoneal chemotherapy. Indian J Anaesth 2020;64(8):688-695. (In eng). DOI: 10.4103/ija.IJA_34_20.

222. Park J, Kim M, Park YH, et al. Comparison of the effects of intravenous propofol and inhalational desflurane on the quality of early recovery after hand-assisted laparoscopic donor nephrectomy: a prospective, randomised controlled trial. BMJ Open 2020;10(12):e039881. (In eng). DOI: 10.1136/bmjopen-2020-039881.

223. Shin S, Kim SH, Park KK, Kim SJ, Bae JC, Choi YS. Effects of Anesthesia Techniques on Outcomes after Hip Fracture Surgery in Elderly Patients: A Prospective, Randomized, Controlled Trial. J Clin Med 2020;9(6) (In eng). DOI: 10.3390/jcm9061605.

224. Weng Y, Yuan S, Li H, Yu W. Comparison of Cardioprotective Effects of Propofol versus Sevoflurane in Pediatric Living Donor Liver Transplantation. Ann Transplant 2020;25:e923398. (In eng). DOI: 10.12659/aot.923398.

225. Chitnis S, Mullane D, Brohan J, et al. Dexmedetomidine Use in Intensive Care Unit Sedation and Postoperative Recovery in Elderly Patients Post-Cardiac Surgery (DIRECT). J Cardiothorac Vasc Anesth 2022;36(3):880-892. (In eng). DOI: 10.1053/j.jvca.2021.09.024.

226*. Dubowitz JA, Cata JP, De Silva AP, et al. Volatile anaesthesia and peri-operative outcomes related to cancer: a feasibility and pilot study for a large randomised control trial. Anaesthesia 2021;76(9):1198-1206. (In eng). DOI: 10.1111/anae.15354.

227. Fang FQ, Sun JH, Wu QL, et al. Protective effect of sevoflurane on vascular endothelial glycocalyx in patients undergoing heart valve surgery: A randomised controlled trial. Eur J Anaesthesiol 2021;38(5):477-486. (In eng). DOI: 10.1097/eja.0000000000001429.

228. Hughes CG, Mailloux PT, Devlin JW, et al. Dexmedetomidine or Propofol for Sedation in Mechanically Ventilated Adults with Sepsis. N Engl J Med 2021;384(15):1424-1436. (In eng). DOI: 10.1056/NEJMoa2024922.

229. Jo JY, Kim YJ, Choi SS, Park J, Park H, Hahm KD. A Prospective Randomized Comparison of Postoperative Pain and Complications after Thyroidectomy under Different Anesthetic Techniques: Volatile Anesthesia versus Total Intravenous Anesthesia. Pain Res Manag 2021;2021:8876906. (In eng). DOI: 10.1155/2021/8876906.

230. Joe YE, Kang CM, Lee HM, Kim KJ, Hwang HK, Lee JR. Quality of Recovery of Patients Who Underwent Curative Pancreatectomy: Comparison of Total Intravenous Anesthesia Versus Inhalation Anesthesia Using the QOR-40 Questionnaire. World J Surg 2021;45(8):2581-2590. (In eng). DOI: 10.1007/s00268-021-06117-0.

231*. Kim NY, Kim KJ, Lee KY, et al. Effect of volatile and total intravenous anesthesia on syndecan-1 shedding after minimally invasive gastrectomy: a randomized trial. Sci Rep 2021;11(1):1511. (In eng). DOI: 10.1038/s41598-021-81012-1.

232. Kim SH, Ju HM, Choi CH, Park HR, Shin S. Inhalational versus intravenous maintenance of anesthesia for quality of recovery in patients undergoing corrective lower limb osteotomy: A randomized controlled trial. PLoS One 2021;16(2):e0247089. (In eng). DOI: 10.1371/journal.pone.0247089.

233. Li XF, Hu JR, Wu Y, Chen Y, Zhang MQ, Yu H. Comparative Effect of Propofol and Volatile Anesthetics on Postoperative Pulmonary Complications After Lung Resection Surgery: A Randomized Clinical Trial. Anesth Analg 2021;133(4):949-957. (In eng). DOI: 10.1213/ane.0000000000005334.

234. Li Y, Chen D, Wang H, et al. Intravenous versus Volatile Anesthetic Effects on Postoperative Cognition in Elderly Patients Undergoing Laparoscopic Abdominal Surgery. Anesthesiology 2021;134(3):381-394. (In eng). DOI: 10.1097/aln.0000000000003680.

235. Liu Y, Ma Y, Liu Y, Wang W, Liu F. Propofol shows less negative effects on cognitive performances than dexmedetomidine in elderly intensive care unit patients. Neurol Sci 2021;42(9):3767-3774. (In eng). DOI: 10.1007/s10072-020-04994-2.

236. Niu Z, Gao X, Shi Z, et al. Effect of total intravenous anesthesia or inhalation anesthesia on postoperative quality of recovery in patients undergoing total laparoscopic hysterectomy: A randomized controlled trial. J Clin Anesth 2021;73:110374. (In eng). DOI: 10.1016/j.jclinane.2021.110374.

237. Siripoonyothai S, Sindhvananda W. Comparison of postoperative delirium within 24 hours between ketamine and propofol infusion during cardiopulmonary bypass machine: A randomized controlled trial. Ann Card Anaesth 2021;24(3):294-301. (In eng). DOI: 10.4103/aca.ACA_85_20.

238. Winings NA, Daley BJ, Bollig RW, et al. Dexmedetomidine versus propofol for prolonged sedation in critically ill trauma and surgical patients. Surgeon 2021;19(3):129-134. (In eng). DOI: 10.1016/j.surge.2020.04.003.

239. Yu H, Xu Z, Dai SH, et al. The Effect of Propofol Versus Volatile Anesthetics on Persistent Pain After Cardiac Surgery: A Randomized Controlled Trial. J Cardiothorac Vasc Anesth 2021;35(8):2438-2446. (In eng). DOI: 10.1053/j.jvca.2020.10.025.

240*. Sato S, Edanaga M, Kondo M, Yamakage M. Effect of desflurane on changes in regional cerebral oxygenation in patients undergoing one-lung ventilation is equivalent to the effect of propofol. Respir Physiol Neurobiol 2022;296:103798. (In eng). DOI: 10.1016/j.resp.2021.103798.

241. Choi EK, Kim S, Kim DY. Effects of propofol-remifentanil versus sevoflurane-remifentanil on acute postoperative pain after total shoulder arthroplasty: a randomized trial. J Yeungnam Med Sci 2022 (In eng). DOI: 10.12701/jyms.2022.00129.

242*. Franzén S, Semenas E, Taavo M, Mårtensson J, Larsson A, Frithiof R. Renal function during sevoflurane or total intravenous propofol anaesthesia: a single-centre parallel randomised controlled study. Br J Anaesth 2022;128(5):838-848. (In eng). DOI: 10.1016/j.bja.2022.02.030.

243. Kawagoe I, Hayashida M, Satoh D, Mitaka C. Comparison of desflurane and propofol in the speed and the quality of emergence from anesthesia in patients undergoing lung cancer surgery-a prospective, randomized study. Transl Cancer Res 2022;11(4):736-744. (In eng). DOI: 10.21037/tcr-21-2635.

244. Kawanishi R, Kakuta N, Sakai Y, et al. Desflurane improves lung collapse more than propofol during one-lung ventilation and reduces operation time in lobectomy by video-assisted thoracic surgery: a randomized controlled trial. BMC Anesthesiol 2022;22(1):125. (In eng). DOI: 10.1186/s12871-022-01669-7.

245. Kim JE, Koh SY, Jun IJ. Comparison of the Effects of Propofol and Sevoflurane Anesthesia on Optic Nerve Sheath Diameter in Robot-Assisted Laparoscopic Gynecology Surgery: A Randomized Controlled Trial. J Clin Med 2022;11(8) (In eng). DOI: 10.3390/jcm11082161.

246. Kim SH, Lee JG, Ju HM, Choi S, Yang H, Koo BN. Propofol prevents further prolongation of QT interval during liver transplantation. Sci Rep 2022;12(1):4636. (In eng). DOI: 10.1038/s41598-022-08592-4.

247. Liu Y, Yu X, Zhu D, et al. Safety and efficacy of ciprofol vs. propofol for sedation in intensive care unit patients with mechanical ventilation: a multi-center, open label, randomized, phase 2 trial. Chin Med J (Engl) 2022;135(9):1043-1051. (In eng). DOI: 10.1097/cm9.0000000000001912.

248. Lu Z, Zheng H, Chen Z, et al. Effect of Etomidate vs Propofol for Total Intravenous Anesthesia on Major Postoperative Complications in Older Patients: A Randomized Clinical Trial. JAMA Surg 2022;157(10):888-895. (In eng). DOI: 10.1001/jamasurg.2022.3338.

249. Mao Y, Guo J, Yuan J, Zhao E, Yang J. Quality of Recovery After General Anesthesia with Remimazolam in Patients' Undergoing Urologic Surgery: A Randomized Controlled Trial Comparing Remimazolam with Propofol. Drug Des Devel Ther 2022;16:1199-1209. (In eng). DOI: 10.2147/dddt.S359496.

250. Wang J, Cui S, Kong L, Ma B, Gu J. Robustness of Propofol and Sevoflurane on the Perioperative Immune Function of Patients Undergoing Laparoscopic Radical Nephrectomy. J Oncol 2022;2022:1662007. (In eng). DOI: 10.1155/2022/1662007.

251. Zhang J, Wang X, Zhang Q, Wang Z, Zhu S. Application effects of remimazolam and propofol on elderly patients undergoing hip replacement. BMC Anesthesiol 2022;22(1):118. (In eng). DOI: 10.1186/s12871-022-01641-5.

252. Zhou Y, Yang J, Wang B, et al. Sequential use of midazolam and dexmedetomidine for long-term sedation may reduce weaning time in selected critically ill, mechanically ventilated patients: a randomized controlled study. Crit Care 2022;26(1):122. (In eng). DOI: 10.1186/s13054-022-03967-5.
